# Characterizing the neighborhood risk environment in multisite clinic-based cohort studies: A practical geocoding and data linkages protocol for protected health information

**DOI:** 10.1371/journal.pone.0278672

**Published:** 2022-12-29

**Authors:** Ariann Nassel, Marta G. Wilson-Barthes, Chanelle J. Howe, Sonia Napravnik, Michael J. Mugavero, Deana Agil, Akilah J. Dulin

**Affiliations:** 1 Lister Hill Center for Health Policy, School of Public Health, University of Alabama at Birmingham, Birmingham, Alabama, United States of America; 2 Center for Epidemiologic Research, Department of Epidemiology, Brown University School of Public Health, Providence, Rhode Island, United States of America; 3 Division of Infectious Diseases, Department of Medicine, School of Medicine, Department of Epidemiology, Gillings School of Global Public Health, University of North Carolina at Chapel Hill, Chapel Hill, North Carolina, United States of America; 4 Division of Infectious Diseases, Department of Medicine, Center for AIDS Research, University of Alabama at Birmingham, Birmingham, Alabama, United States of America; 5 Center for Health Promotion and Health Equity, Department of Behavioral and Social Sciences, Brown University School of Public Health, Providence, Rhode Island, United States of America; University of North Carolina at Chapel Hill Davis Library: The University of North Carolina at Chapel Hill, UNITED STATES

## Abstract

**Background:**

Maintaining patient privacy when geocoding and linking residential address information with neighborhood-level data can create challenges during research. Challenges may arise when study staff have limited training in geocoding and linking data, or when non-study staff with appropriate expertise have limited availability, are unfamiliar with a study’s population or objectives, or are not affordable for the study team. Opportunities for data breaches may also arise when working with non-study staff who are not on-site. We detail a free, user-friendly protocol for constructing indices of the neighborhood risk environment during multisite, clinic-based cohort studies that rely on participants’ protected health information. This protocol can be implemented by study staff who do not have prior training in Geographic Information Systems (GIS) and can help minimize the operational costs of integrating geographic data into public health projects.

**Methods:**

This protocol demonstrates how to: (1) securely geocode patients’ residential addresses in a clinic setting and match geocoded addresses to census tracts using Geographic Information System software (Esri, Redlands, CA); (2) ascertain contextual variables of the risk environment from the American Community Survey and ArcGIS Business Analyst (Esri, Redlands, CA); (3) use geoidentifiers to link neighborhood risk data to census tracts containing geocoded addresses; and (4) assign randomly generated identifiers to census tracts and strip census tracts of their geoidentifiers to maintain patient confidentiality.

**Results:**

Completion of this protocol generates three neighborhood risk indices (i.e., Neighborhood Disadvantage Index, Murder Rate Index, and Assault Rate Index) for patients’ coded census tract locations.

**Conclusions:**

This protocol can be used by research personnel without prior GIS experience to easily create objective indices of the neighborhood risk environment while upholding patient confidentiality. Future studies can adapt this protocol to fit their specific patient populations and analytic objectives.

## Introduction

In the United States (US), disadvantaged and socially disordered neighborhoods–those characterized by structural racism that leads to scarcity of resources, poverty, low high school completion rates, racial minority segregation, and/or violence–can have direct and indirect negative effects on health [1–5]. For example, individuals residing in disadvantaged and disordered neighborhoods are more likely to experience multiple stressors [6, 7] and increased risk of chronic disease such as heart disease, hypertension, and HIV [3, 8–12] compared to residents of more advantaged and less disordered neighborhoods. Despite the well-established and growing literature surrounding neighborhood effects on health [13, 14], the methods that drive this research vary widely and continue to change over time [15–19]. Thus, there is motivation to develop new, accessible and reproducible tools that perform consistently across studies and can be adapted to diverse research objectives.

Geocoding (assigning a latitude and longitude coordinate to a postal address) is one available tool that is increasingly used in public health [20, 21] to help researchers characterize neighborhood environments and assess how individual-level health outcomes vary due to differences in these environments. However, challenges can arise when using geocoding in research studies that rely on participants’ protected health information (PHI), which includes residential address information [22]. Some of the current geocoding approaches rely on sharing de-identified health data outside of the setting in which the data were originally generated so that external mapping experts can conduct in-depth analysis [23]. Alongside potentially incurring additional costs to a project, this “out of house” data sharing approach can create opportunities for data breaches when residential address locations can potentially be identified from published maps via techniques such as reverse geocoding [24], spatial re-engineering [25] and digital scanning [25]. Other geocoding approaches rely on online, cloud-based services [26] such as Google Maps, ArcGIS Online, Census Geocoder, or OpenStreetMap [27, 28], which can create additional opportunities to unlawfully access PHI if cloud-based geocoding is conducted behind an identifiable Internet Protocol (IP) address.

Protecting geocoded patient address data can present additional challenges during multisite clinic-based cohort studies. Multisite studies must frequently choose between performing geocoding and data linkages “in-house” to protect participants’ PHI or involving the external expertise of a geoscientist with specific knowledge of geographic information systems (GIS). For many institutions, the former “in-house” approach will be limited to the GIS knowledge of research personnel [23, 29]. For the latter approach, involving an external geospatial collaborator can require lengthy approvals from the governing Institutional Review Board (IRB) which can delay timely data collection [23]. An external GIS specialist may also be unfamiliar with a site’s specific patient population(s), which can introduce errors during data merging and analyses. Lastly, ensuring comparable levels of geocoding accuracy can be difficult if or when the same geocoding techniques are not uniformly applied across sites.

To address these challenges, we present a publicly available geocoding protocol for characterizing the neighborhood risk environment that can be implemented during multisite clinical cohort studies working with PHI. The protocol can be understood and implemented by staff without prior GIS experience to geocode patients’ residential addresses, link these addresses to crime and socioeconomic data, and create anonymized coded census tracts to construct objective indices of the neighborhood risk environment. The process of anonymizing census tracts via this protocol can enable analyses to be performed at the individual-level while ensuring a patient’s PHI is not linked to census-designated geoidentifiers. This protocol is informed by our prior experience [30] geocoding and linking patient data from two HIV clinic-based cohorts in the United States [31–33] and offers a standardized, user-friendly tool that can be adapted for future studies.

## Materials and methods

**Fig 1:**
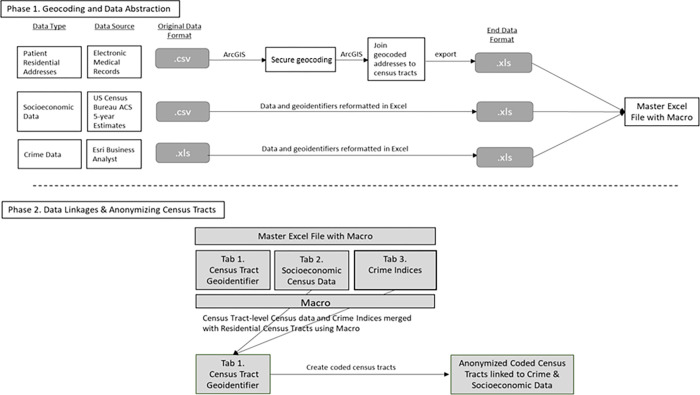
Protocol flow diagram. Visual depiction of each phase of the geocoding and data linkages protocol. ACS: American Community Survey.

This protocol follows two main phases based on our work [30]. Phase 1 includes (1) geocoding patients’ residential addresses and joining geocoded addresses to census tracts, and (2) abstracting indicators of neighborhood crime and socioeconomic disadvantage at the census tract level. Phase 2 includes (1) linking the aforementioned patient- and neighborhood-level data by census tract, and (2) creating coded census tracts to anonymize the census tract within which a patient lives. All data in this protocol are illustrative and do not include any actual patient information. Fig 1 summarizes the geocoding and data linkages process.

The full geocoding and data linkages protocol is provided in S1 File and publicly available on the protocols.io platform at dx.doi.org/10.17504/protocols.io.b3dvqi66 [PROTOCOL DOI].

### Phase 1: Geocoding and data abstraction

#### 1A. Geocoding residential addresses and joining geocoded addresses to census tract boundaries

*Objective*: Geocode patients’ residential addresses and link the geocoded address locations to US census tracts

*Requirements*: Esri ArcGIS 10.5.1 or later; StreetMap Premium Software for Esri ArcGIS 10.5.1 or later (annual license required)

*Detailed instructions*: Part 3 of S1 File

To begin implementing this protocol, residential address information should be abstracted from patient medical records and saved locally (e.g., on a secure server protected by the clinic’s firewall) as an Excel Comma-Separated Values (CSV) file. Address information should contain, at minimum, the street address, city, state, and zip code for each patient record. Parts 1 and 2 of the protocol (S1 File) provide instruction for formatting residential address information and preparing to geocode.

Clinic-based research staff should use the latest available version of ArcGIS Desktop and StreetMap Premium (Esri, Redlands, CA) to geocode patients’ residential addresses within a computing environment that meets security requirements for storing PHI [21, 34]. If an address cannot initially be geocoded, a rematch should be run in ArcMap and a suggested address with a minimum match score of 85% should be selected [35, 36]. The match score indicates how closely a residential address matches the geospatial reference data in ArcGIS, where a score of 100% indicates a perfect match. Addresses outside of the study state or Post Office Boxes cannot be assumed to approximate a patient’s neighborhood environment and should not be geocoded.

The geographic coordinates for each patient’s residential address should then be joined to a data layer of census tract boundaries by performing a spatial join in ArcMap. Geocoded addresses linked to census tract boundaries should be abstracted from the ArcMap Attribute Table and reformatted in Excel to include the minimum data fields required to for census tract identification.

#### 1B. Abstracting crime indicators from Esri’s business analyst online

*Objective*: Ascertain neighborhood crime risk indicators for census tracts containing geocoded patient addresses

*Requirements*: Esri Business Analyst Online version 5.82 or later

*Detailed instructions*: Part 4 of S1 File

Indicators of neighborhood crime risk should be obtained at the census tract level using the latest available version of ArcGIS Business Analyst Online (Esri, Redlands, CA), a web-based program offering additional de-identified census-level data (e.g., crime, business, sociodemographic). In this example protocol, two neighborhood crime indices–a Murder Rate Index and an Assault Rate Index–are obtained from Business Analyst because of their known associations with HIV outcomes [37–40]. Business Analyst derives its data from Applied Geographic Solutions (AGS) CrimeRisk© indices (Applied Geographic Solutions, 2020B) [41, 42] which are based on FBI Uniform Crime Reports and local police departments. Other crime indices include, but are not limited to, Rape, Burglary, and Motor Vehicle Theft Indices and composite indices of Personal, Property, and Total Crime. Alternative crime indices can be selected from Business Analyst to reflect a study’s specific neighborhood environment.

Within Business Analyst, the geography should be set to census tracts and each census tract within the study state should be selected. Using the SmartMap Search feature, data for the relevant crime indicators should be selected and downloaded as a CSV file. Downloaded data will contain a numeric census tract identifier and data for the relevant index. This process will need to be repeated in stages because Business Analyst does not permit data abstraction for all census tracts at one time.

#### 1C. Abstracting socioeconomic indicators from data.census.gov

*Objective*: Ascertain neighborhood socioeconomic data for census tracts containing geocoded patient addresses

*Requirements*: American Community Survey Five-Year Estimates from the US Census Bureau

*Detailed instructions*: Part 5 of S1 File

Census tract level-indicators of socioeconomic disadvantage should be obtained using the US Census Bureau’s American Community Survey (ACS) 5-year estimates that most closely align with the study period (i.e., the most recent 5-year estimates for prospective studies or earlier 5-year estimates for retrospective studies) [43]. Compared to single-year and three-year estimates, ACS 5-year estimates provide increased statistical reliability of the data particularly for smaller geographic areas and subpopulations [44]. In this example protocol, three socioeconomic indicators–unemployment, high school education, and poverty–are obtained from ACS based on their known associations with HIV outcomes [37–40]. Alternative socioeconomic indicators can be selected from the more than 20,000 variables available in ACS [43].

Using the Advanced Search option in data.census.gov, the relevant Table ID number should be entered and all census tracts within the study state should be selected and downloaded as a CSV file. Identifying the Table that contains study-specific variables can be done by typing the subject area of interest into the Explore Census Data search box at data.census.gov. Downloaded data will contain a numeric census tract identifier, a census tract label, and data for relevant socioeconomic indicator(s). In this example protocol, only percentage estimates of each indicator are preserved for data linkages but other formats (e.g., total or median estimates) can be selected from the downloaded data.

### Phase 2: Data linkages & anonymizing census tracts

#### 2A. Linking geocoded addresses and abstracted neighborhood risk data by census tract

*Objective*: Link abstracted socioeconomic and crime risk data to each census tract containing a geocoded patient address

*Requirements*: Macro-enabled Excel File (S2 File)

*Detailed instructions*: Part 6 of S1 File

In this step, four CSV files from Phase I should be linked using the Macro-enabled Excel file (version 2019) developed for this protocol. (S2 File) The first file will contain geocoded patient address information, the second file will contain census tract-level crime risk data, the third file will contain census tract-level poverty and unemployment data (or other study-specific socioeconomic data), and the fourth file will contain census-tract level education data (or other study-specific socioeconomic data). The geoidentifiers and associated data in these four files should be copied into the relevant sheets in the Excel file and the customized Macros–“ACSMatcher” and “CrimeMatcher”–should be run to link all data by census tract. Census tracts containing geocoded addresses may include missing data for some or all neighborhood risk indicators depending on the data available in ACS and Business Analyst at the time of abstraction. Though not required for this protocol, users wishing to edit or reference the coding of the ACSMatcher and/or CrimeMatcher Macros can do so by clicking “Macros”, “View Macros” and then “Edit” in S2 File.

#### 2B. Creating coded census tracts

*Objective*: Anonymize census tracts that are linked to neighborhood risk data and contain geocoded patient addresses

*Requirements*: Macro-enabled Excel File (S2 File)

*Detailed instructions*: Part 7 of S1 File

**Fig 2:**
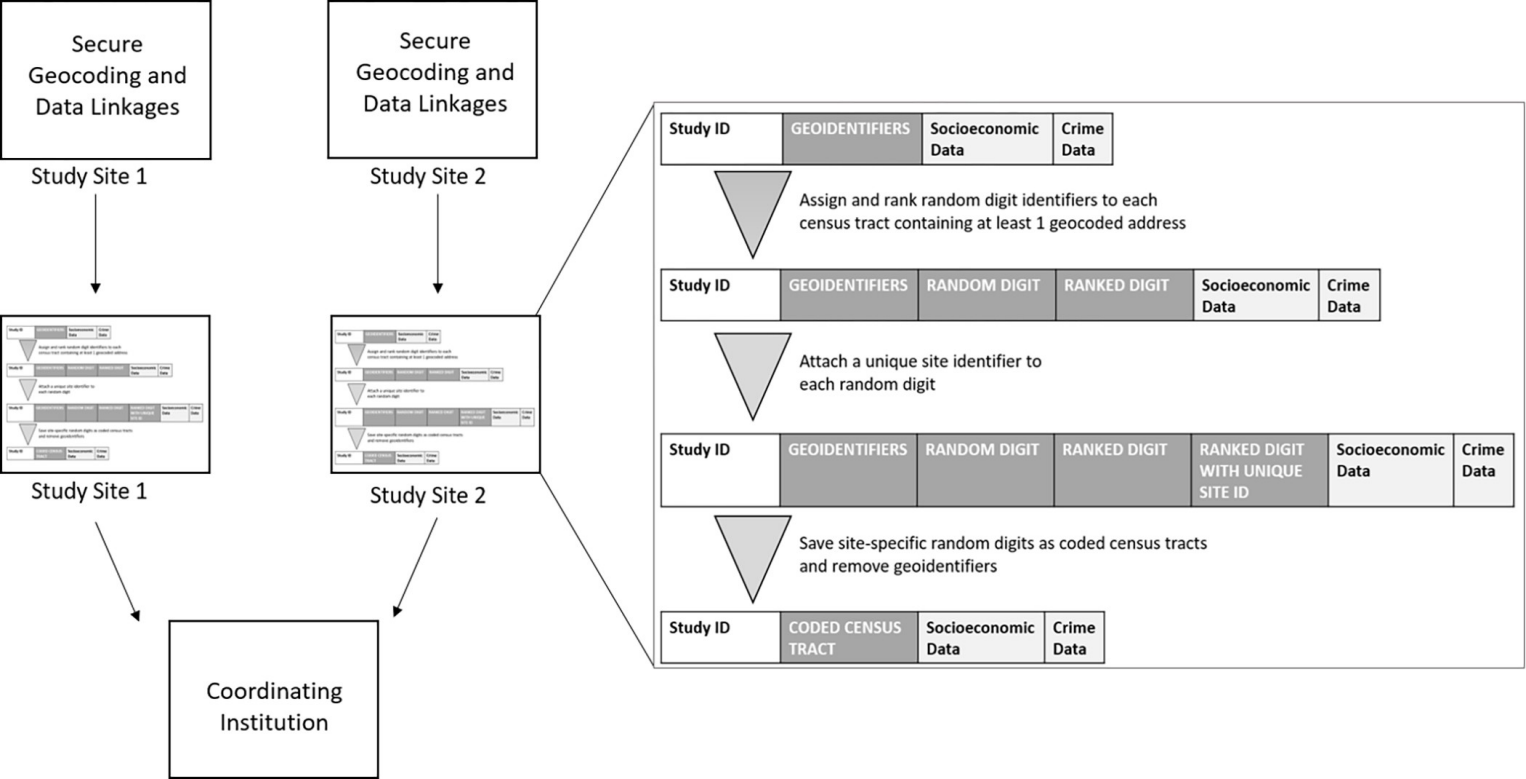
Process for creating coded census tracts during multisite study. Process for stripping census tracts containing geocoded patient address information of their census-designated identifiers and assigning a randomly generated number to create anonymized “coded” census tracts. The design of Fig 2 was adapted from a figure published by Brokamp et al., 2018 in the open-access journal *J Am Med Inform Assoc* [51].

To minimize the risk of patients being identified from their census tract information, census tracts that have been linked to socioeconomic and crime risk data will need to be stripped of their census-designated geoidentifiers and assigned a randomly generated number to create anonymized “coded” census tracts. First, for each study record in the Excel file, a random digit should be generated using Excel’s “RAND” function. Second, each randomly generated digit should then be ranked using Excel’s “RANK” function to convert random digits to whole integers. Third, a unique site identifier should be attached to each ranked digit using Excel’s “CONCATENATE” function. Assigning a site-specific identifier to the coded census tract prevents duplication of coded census tracts across study sites. Last, each record should be stripped of its original census tract identifiers so that only the coded census tract is preserved. Since multiple patients may reside within the same census tract, Excel’s “Conditional Formatting” function should be used to ensure duplicate census tracts within each clinic site are assigned the same coded census tract identifier. Fig 2 diagrams the process for creating coded census tracts.

Creating coded census tracts ensures that (1) a single residential address cannot be identified during analysis that occurs outside of the clinic setting where the data were originally generated, and (2) the data layer cannot be mapped or visually displayed.

The Excel file containing a study ID, coded census tract, and the linked socioeconomic and crime risk data for each record can then be sent from each clinic site to the coordinating institution for analysis using IRB-approved (if necessary) data sharing platforms. The coordinating institution can use the study ID assigned to each participant’s record to merge coded census tract data from multiple sites.

## Results

Successfully implementing this protocol will yield indices of neighborhood crime and generate an index of neighborhood disadvantage at the census tract level (Table 1).

**Table 1:** Resulting indices of the neighborhood risk environment in example multisite study.

	EXAMPLE MIN, MAX RANGE OF INDEX DATA	EXAMPLE CATEGORIZATION OF RISK LEVELS		
		Median Split	Tertiles	Quartiles
Neighborhood Disadvantage Index^a^	-2.36, 9.42	0 = Low risk 1 = High risk	1 = Low risk 2 = Moderate risk 3 = High risk	1 = Low risk 2 = Moderate-low risk 3 = Medium-high risk 4 = High risk
Murder Rate Index^b^	16, 1109	0 = Low risk 1 = High risk	1 = Low risk 2 = Moderate risk 3 = High risk	1 = Low risk 2 = Moderate-low risk 3 = Medium-high risk 4 = High risk
Assault Rate Index^b^	6, 899	0 = Low risk 1 = High risk	1 = Low risk 2 = Moderate risk 3 = High risk	1 = Low risk 2 = Moderate-low risk 3 = Medium-high risk 4 = High risk

Index data are illustrative and do not include any actual patient information.

^a^ The range of values for the Neighborhood Disadvantage Index represents the sum of the Z-scores for each socioeconomic variable (e.g., poverty, education, unemployment) that are normed to the national (US) level.

^b^ At the US level, a fixed value of 100 is assigned to the murder and assault rate indices such that a value > 100 for a given census tract denotes an increased relative risk of murder or assault, and a value < 100 denotes a lower relative risk of murder or assault compared to the national average.

The Neighborhood Disadvantage Index represents a summation of the census-tract level z-scores for each socioeconomic variable that have been normed to the national level. For example, a Z-score of 1 indicates one standard deviation greater disadvantage compared to the US. The coordinating institution can construct the Neighborhood Disadvantage Index once all site data have been merged. (S3 File provides step-by-step instructions for how to construct the Neighborhood Disadvantage Index.)

Crime indices are directly abstracted from Esri’s Business Analyst. These indices provide an indication of the relative risk of a crime occurring measured against the overall risk for the US. When a study participant’s coded census tract contains a value greater than 100 for the relevant crime index (e.g., for the Murder Rate Index as in this example protocol), this denotes an increased relative risk of murder or assault compared to the US. Conversely, a value less than 100 denotes a lower relative crime risk compared to the national level.

Depending on the size of the study population, continuous index data can be transformed into risk categories (e.g., binary splits, tertiles) by using cut-points appropriate for the sample distribution [45].

## Discussion

As population health and health equity research increasingly focus on health determinants beyond the individual-level, practical and implementable tools that characterize the neighborhood environment while protecting individual confidentiality are paramount. This protocol offers a free, publicly available and user-friendly resource for generating measures of the neighborhood risk environment during multisite studies that rely on PHI. We effectively implemented this protocol in our prior work to identify multilevel resilience resources among African American/Black (AA/B) adults living with HIV [30]. We found in this prior work that despite the majority of study participants residing in more economically disadvantaged and higher crime neighborhoods compared to the US, AA/B adults living with HIV still identified resilience resources that helped them engage in care.

Our protocol offers four key improvements over current approaches to geocoding and data linkages [23, 26–28]. First, an ongoing concern during clinic-based studies is the confidentiality of PHI. A main advantage of this protocol is that it should satisfy any site-specific security or IRB requirements to obtain census tract-level data because all geocoding and data linkages occur at the cohort site, which eliminates the need for PHI to leave the clinic-cohort site. Even for census tracts containing only one geocoded address, our process for creating coded census tracts ensures that a patient’s exact residential location cannot be known or visually displayed while allowing census tracts to be distinguishable across multiple sites. A second challenge during multisite studies is ensuring that geocoding and data linkages are performed systematically within and between sites. The Macros developed for this protocol provide an automated process for linking neighborhood-level indicators to census tract identifiers regardless of the number of participants enrolled at a given site or number of sites performing the linkages. Use of the Macros can help reduce human error and cognitive burden. Third, this protocol draws from the cadre of established methods [20, 21] for geocoding and linking residential data with neighborhood information. Using simple, validated methods rather than more complex geocoding approaches increases the likelihood that research personnel without prior GIS training will be able to implement this protocol with minimal oversight. Strengthening the geoanalytic skills of on-site study staff who are directly involved in a research project negates the need to solicit GIS expertise from non-study staff, which can help conserve time and resources and prevent exposing PHI to a third party. Last, a key advantage of this protocol is the flexibility of the variable/indicator selection process. As previously mentioned, the example contextual markers of neighborhood disadvantage and crime were informed by our prior experience [30] working with HIV clinical cohorts and evidence demonstrating associations between these markers and HIV outcomes [37–40]. However, the same data abstraction process can be used to select indicators of crime and neighborhood disadvantage that are study-specific.

The current protocol utilizes a Murder Rate Index and an Assault Rate Index derived from the AGC CrimeRisk© indices. Other studies have used these indices, including to examine associations between neighborhood crime and sexual risk-taking behaviors among Black men living with HIV [46] and to assess for interactions between neighborhood crime, childhood trauma and longer-term mental health outcomes [47]. This protocol also utilizes Census Bureau ACS 5-year estimates. Prior large-scale studies have utilized ACS 5-year estimates, including to investigate county-level disparities in COVID-19 cases and deaths among racial/ethnic minority groups across the US [48]. These and other examples [49, 50] demonstrate that this protocol’s design is relevant for the current field of neighborhood health effects research and can be applied readily in future work.

Alongside this protocol, other efforts have aimed to develop standardized protocols for contextualizing neighborhood environments in research. The Decentralized Geomarker Assessment for Multi-Site Studies (DeGAUSS) software is one example tool that can facilitate reproducible geocoding while maintaining patients’ confidentiality [51]. The software is free and does not expose PHI to an intermediary party. However, to the best of our knowledge, DeGAUSS was tested during an initial proof-of-concept study but has yet to be extended to research requiring the anonymization of census tracts. The Women’s Interagency HIV Study (WIHS) [52] has also developed several versions of its geocoding protocol for PHI [53, 54] to help evaluate relationships between neighborhood-level poverty and disease control among women living with or at risk for chronic illness [55]. The WIHS protocol relies on Federal Information Processing Standards (FIPS) codes to define geographies across 10 cohort sites. FIPS codes have been published in census products for over 30 years and are widely used in public health geocoding [56]. In comparison, the current protocol uses census-derived identifiers because not all statistical geographic areas are covered by FIPS codes and a census-derived identifier can encompass both FIPS and Census Bureau codes [56]. The geocoding protocol developed for the Jackson Heart Study (JHS) [57] has also demonstrated considerable accuracy as a geocoding tool, retrospectively geocoding nearly 99% of the JHS cohort after address data were not obtained at baseline. The JHS protocol relied on georeferencing participants to census block groups while the current protocol utilizes census tracts which contain a greater number of residents, thereby minimizing the risk of identifying enrolled patients [58].

A limitation of this protocol is its reliance on the Esri StreetMap Premium annual license. The license can incur additional study costs if the product is not readily available at the institution or if geocoding activities span more than one year. Researchers choosing to include crime risk data in the neighborhood risk indices could also incur costs if additional Business Analyst credits (i.e., the currency used by ArcGIS) need to be purchased to facilitate the download of large amounts of data. However, the number of credits consumed by downloading data from Business Analyst for this protocol is quite small (e.g., 10 credits needed for a single download compared to the 6,000–10,000 credits included with an annual license [59]). Affiliates of any institution that licenses Esri software should have access to all data (including census-tract level crime risk data) within ArcGIS Business Analyst Online for no additional fee. Another limitation is that patient address information is derived from medical records rather than direct self-report. Thus, the percentage of records that can be matched to census tract boundaries will be predetermined by the data available in patients’ medical records. This protocol is also limited to measuring the physical neighborhood environment using objective data from the US census and other sources (e.g., Business Analyst), even though self-report can more accurately characterize neighborhood exposures in some instances [60, 61]. Also, while the anonymization of census tracts serves to safeguard PHI, it prohibits patients’ locations from being mapped or visually displayed. For studies aiming to map patients’ census tract locations or perform spatial analyses of disease dynamics, other methods may be more appropriate [62–64]. Finally, while this protocol has been successfully implemented by research personnel with minimal or no prior GIS experience [30, 65], other project teams may wish to identify a GIS expert prior to study start who is available to answer protocol implementation questions if or when they arise.

### Conclusions

This step-by-step geocoding and data linkage protocol addresses some of the common challenges of working with PHI and offers an adaptable resource to help assess neighborhood-level health impacts during multisite studies. Such resources can support population health research and multilevel interventions that aim to mitigate adverse neighborhood effects on health.

## Supporting information

S1 File Geocoding and data linkages protocol for protected health information (v2021). (PDF)

S2 File Macro-enabled excel file. Macro-enabled Excel file that can be used to (1) Link census tracts containing patient geocoded addresses to indicators of neighborhood crime and socioeconomic disadvantage using the census tract geoidentifier, and (2) Assign randomly generated identification numbers to census tracts and strip them of geoidentifiers to maintain patient confidentiality.
(XLSM)

S3 File Instructions for neighborhood disadvantage index construction. (PDF)

## List of abbreviations

AA/B: African American/Black
ACS: American Community Survey
AGS: Applied Geographic Solutions
BAO: Business Analyst Online
CSV: Comma-Separated Values
GIS: Geographic Information System
HIV: Human Immunodeficiency Virus
IRB: Institutional Review Board
PHI: Protected Heath Information
