# Supplementary material for: Characterizing the neighborhood risk environment in multisite clinic-based cohort studies: A practical geocoding and data linkages protocol for protected health information
Source: PLoS One. 2022 Dec 29;17(12):e0278672. doi: 10.1371/journal.pone.0278672 (PMC9799318; doi:10.1371/journal.pone.0278672)
Supplement: S1 File — (PDF) [file pone.0278672.s001.pdf]

Supplementary material for the manuscript:

Characterizing the Neighborhood Risk Environment in Multisite Clinic-Based Cohort Studies: A Practical Geocoding and Data Linkages Protocol for Protected Health Information

Ariann Nassel[1¶], Marta G. Wilson-Barthes[2¶*], Chanelle J. Howe[2], Sonia Napravnik[3], Michael J. Mugavero[4], Deana Agil[3], Akilah J. Dulin[5]

[¶] Ariann Nassel and Marta G. Wilson-Barthes are co-first authors; both first authors contributed equally to this manuscript.

[*] Corresponding author
Email: marta_wilson-barthes@brown.edu (MWB)

[1] Lister Hill Center for Health Policy, School of Public Health, University of Alabama at Birmingham, Birmingham, Alabama, United States of America.
[2] Center for Epidemiologic Research, Department of Epidemiology, Brown University School of Public Health, Providence, Rhode Island, United States of America.
[3] Division of Infectious Diseases, Department of Medicine, School of Medicine, Department of Epidemiology, Gillings School of Global Public Health, University of North Carolina at Chapel Hill, Chapel Hill, North Carolina, United States of America.
[4] Division of Infectious Diseases, Department of Medicine, Center for AIDS Research, University of Alabama at Birmingham, Birmingham, Alabama, United States of America.
[5] Center for Health Promotion and Health Equity, Department of Behavioral and Social Sciences, Brown University School of Public Health, Providence, Rhode Island, United States of America.

Geocoding and Data Linkages Protocol for Protected Health Information

2021 Version

Table of Contents

Introduction 4

Part 1: Preparing to Geocode Residential Addresses, Link addresses to Census Tracts, Abstract Socioeconomic and Crime Data, and Link All Data in the Master Data File 6

Part 2: Preparing the File Containing Patient Residential Addresses 7

Part 2.1 Formatting the address file when study data are collected at a single time point 7

Part 2.2 Formatting the address file when study data are collected at multiple time points 7

Part 3a: Geocoding Patient Residential Addresses 9

Step 1. Preparing to Geocode 10

Step 2. Geocoding Residential Addresses 13

Part 3b: Joining Geocoded Address Points to Census Boundaries 24

Step 1. Add the data layer containing the census tract boundaries to the MXD 24

Step 2: Joining Geocoded Address Locations to Census Tracts 27

Step 3. Obtaining and formatting the joined census tract data 30

Understanding your census tract data: 34

Step 4. Identifying the counties that contain addresses geocoded to census tracts 35

Part 4: Obtaining Crime Data 39

Step 1. Accessing Esri Business Analyst Online (BAO) 39

Step 2. Obtaining Crime Data from BAO 40

Step 3: Creating a single Excel file of crime data for all counties in your state 55

Step 4. Reformatting the Esri Census Tract Numbers 57

Part 5a: Obtaining Neighborhood Socioeconomic Data for Census Tracts 61

Step 1. Accessing the data.census.gov Website 61

Step 2. Obtaining Data for % Below the Poverty Line and % Unemployment 62

Step 3. Obtaining Data for % Less than a High School Education 65

Part 5b. Reformatting the files that contain socioeconomic data 69

Reformatting the file that contains data for % Poverty and % Unemployed 69

Reformatting the file that contains data for % less than a high school education 71

Part 6a. Adding abstracted and geocoded data into the Master Excel file 74

Step 1. Adding geocoded address data that are linked to census tracts to the Master file 74

Step 2. Adding data for % less than a high school education to the Master file 76

Step 3. Adding data for % poverty and % unemployed to the Master file 77

Step 4. Adding crime data to the Master file 78

Part 6b. Linking data by Census tractin the Master file 80

Part 7. Deleting Census Tract Identifiers and Creating Coded Identifiers 84

Introduction

GEOCODING METHODS OVERVIEW

Residential address information (i.e., addresses and dates of residence) will be abstracted from patient medical records by project personnel at the clinic-based cohort site. Project personnel will then link the abstracted residential address information to U.S. census tracts. The U.S. census tracts will in turn be linked to crime and socioeconomic indicators (i.e., % below poverty line, % less than a high school education, % unemployed, the murder rate index, and the assault rate index) to develop a neighborhood risk index.

Preparing residential addresses: When study data are collected at a single time point, the address at enrollment will be the address that is geocoded. When study data are collected at multiple time points, addresses will be geocoded patient data are ascertained during the follow-up period.

Geocoding residential addresses and joining addresses to Census Tracts: Each project site will complete geocoding using ArcGIS 10.5.1 (or higher) and StreetMap Premium Software® developed by Esri. Abstracted residential address information will be geocoded by assigning each address geographic coordinates that correspond to the geographic location of that address. Then, the geographic coordinates will be joined to a data layer of census tract boundaries to determine both the county and census tract that contain the participant's residential address location. Geocoded addresses will be matched to the U.S. census tracts in ArcGIS using the Spatial Join Tool. This enables the GIS software to calculate the number of residential locations that fall within the boundaries of each census tract. The number of residential locations that fall within each census tract will be retained within the GIS database by the clinic-based project personnel at the clinic site. However, project personnel will discard each abstracted individual residential address from the GIS database prior to dissemination to external investigators.

Linking geocoded addresses to neighborhood data: A number of contextual variables that are markers of neighborhood risk environments will be obtained from United States Census Bureau and Esri databases and linked to census tracts. The geoidentifier for each census tract that contains residential addresses will be used to match the corresponding neighborhood crime and socioeconomic data to each census tract. Linkage of addresses to census tracts will be done at the at the clinic-based cohort site. De-identified data and coded census tracts will be shared with external, collaborating sites. These shared/analyzed data will not contain addresses, census tract numbers or other identifiers.

DATA SECURITY

1. **SECURITY FOR PATIENT ADDRESSES**
   Patient addresses will reside at your site ONLY. Each site will need to adhere to protocols approved by their local IRB that outline procedures for proper storage of these sensitive data.

2. **SECURITY FOR CENSUS TRACTS**
A census tract is a unit of analysis at which neighborhoods commonly are defined and measured. A census tract contains 4,000 residents on average (range: 1,200 to 8,000). Census tracts are selected for this protocol because they can be used to approximate neighborhood environments and because census tracts contain a sufficient number of inhabitants, on average, to help minimize the risk of identifying enrolled patients.

De-identifying census tracts: Once the census tracts have been linked to the socioeconomic and crime data, clinic-based project personnel will 1) assign the census tracts a randomly generated number and will strip the census tracts of their census designated number and 2) discard the residential addresses that were abstracted from enrolled patient medical records. This stripping will ensure that the exact census tract location cannot be known and that the data layer cannot be mapped or visually displayed. This method of de-identifying each census tract will also ensure that there is no way for a single residential address to be identified even with the planned linkages to socioeconomic and crime data. The census tract that has been assigned a randomly generated number is referred to as the coded census tract.

3. **SECURITY FOR TRANSFERING DATA TO EXTERNAL SITES**
The file containing only the coded census tract information, the socioeconomic data, the crime data, the date of residence, and the REDCap ID (or other study-specific ID) for each enrolled participant will be uploaded following local IRB approved procedures for uploading and encrypting sensitive files.

Part 1: Preparing to Geocode Residential Addresses, Link addresses to Census Tracts, Abstract Socioeconomic and Crime Data, and Link All Data in the Master Data File

This GIS Protocol provides instructions to:

1) Prepare the file containing patient residential addresses (as described in **Part 2** of this protocol),
2) Geocode patient residential addresses (as described in **Part 3a** of this protocol),
3) Join geocoded residential addresses with census tract boundaries (as described in **Part 3b** of this protocol),
4) Abstract and format crime data (as described in **Part 4** of this protocol),
5) Abstract and format socioeconomic data (as described in **Parts 5a and 5b** of this protocol),
6) Merge the socioeconomic data, crime data, and geocoded address data that are joined to US Census tracts in the Master Excel file (described in **Part 6** of this protocol), and
7) Strip US Census tracts of their original identifiers and assign a randomly generated coded identifier (described in **Part 7** of this protocol)

Prior to abstracting any patient residential addresses (as described in **Part 2** of this protocol), each clinic site will need to have access to the following software and online databases:

- ArcGIS 10.5.1 or later
- Esri StreetMap Premium for your state (annual license required)
- Esri Business Analyst Online Version 5.82 or later
- United States Census Bureau data.census.gov

Each clinic cohort site will also need to save the Master Excel file (Provided in Appendix 2 of the corresponding manuscript) to their Desktop. This file contains macros and will be used to link all data during **Part 6** of this protocol.

Part 2: Preparing the File Containing Patient Residential Addresses

Objective: To abstract patient residential addresses from patients' medical records. Completing this objective will yield an Excel file of patient addresses that will be used to conduct the geocoding described in Part 3.

Prior to geocoding the residential addresses, each clinic site will need to abstract the address from each patient's medical record. Each site should follow its IRB approved protocol for abstracting and storing residential addresses.

Reminders:

- For patients who have a Post Office (P.O.) Box as an address, you will need to keep the Study ID (e.g., REDCap_ID in this example) but delete all of the corresponding address information for that Study ID. P.O. boxes are not representative of a patient's residential address and should not be geocoded.
- For patients who have an address outside of the study state, you will need to keep the Study ID (e.g., REDCap_ID in this example) but delete all of the corresponding address information for that ID. Residential addresses outside of study state will not be able to be linked to census tracts using the StreetMap Premium license and should not be geocoded.
- Please make sure to retain the Study ID for all patients in the Excel file.

This Excel file should be saved as both a **.xlsx** file and a **.csv** file. The **.csv** file will be used for geocoding the residential addresses in ArcMap GIS 10.5.1. These files should be saved in a new folder titled "Residential Addresses" that is saved on the Desktop on the password-protected computer at the clinic site.

Part 2.1 Formatting the address file when study data are collected at a single time point

When study data are collected solely at a single time point during your study, the address at enrollment will be the address that is geocoded. Each site should create an Excel file with the following column headings that should be formatted as shown in the below faux file:

**REDCap_ID**	**Address**	**City**	**State**	**Zip**	**County**
172-1	174 North Country Ct.	Miller Place	NC	11766	Suffolk
172-2	11 Shore Rd.	Mt. Sinai	AL	02341	Nassau
172-3	52 Water Ave.	Commack	AL	21203	Oakland
172-4	~~P.O. Box 123~~	~~Columbus~~	~~NC~~	~~44912~~	~~Franklin~~
172-5	~~931 Whitmarsh St.~~	~~Victoria~~	~~VA~~	~~09815~~	~~Dundalk~~

Part 2.2 Formatting the address file when study data are collected at multiple time points

When study data are collected at multiple time points during your study, the address at enrollment and during follow up (e.g., when data are collected during each study visit) will be the addresses that will be geocoded. You will need to geocode the residential address for each enrollment and follow-up visit that a given patient attends, even if the patient does not move residential addresses during the follow-up period. Each site should create an Excel file with the following column headings that should be formatted as shown in the below faux file:

REDCap_ID	Address	City	State	Zip	County
172-1	174 North Country Ct.	Miller Place	NC	11766	Suffolk
172-1	174 North Country Ct.	Miller Place	NC	11766	Suffolk
172-1	174 North Country Ct.	Miller Place	NC	11766	Suffolk
172-1	174 North Country Ct.	Miller Place	NC	11766	Suffolk
172-2	11 Shore Rd.	Mt. Sinai	AL	02341	Nassau
172-2	11 Shore Rd.	Mt. Sinai	AL	02341	Nassau
172-2	107 Princeton Rd.	Sequoia	AL	02091	Riverdale
172-3	52 Water Ave.	Commack	AL	21203	Oakland
172-3	301 Sundale St.	Bedford	AL	37203	Arundel
172-3	301 Sundale St.	Bedford	AL	37203	Arundel
172-4	~~P.O. Box 123~~	~~Columbus~~	~~NC~~	~~44912~~	~~Franklin~~
172-4	~~P.O. Box 123~~	~~Columbus~~	~~NC~~	~~44912~~	~~Franklin~~
172-4	~~P.O. Box 123~~	~~Columbus~~	~~NC~~	~~44912~~	~~Franklin~~
172-5	~~931 Whitmarsh St.~~	~~Victoria~~	~~VA~~	~~09815~~	~~Dundalk~~

This was the last activity needed to abstract residential addresses. The next step, Part 3a, will instruct you on how to geocode patients' residential address.

Part 3a: Geocoding Patient Residential Addresses

Program: StreetMap Premium Software in ArcGIS version 10.5.1 or later.

Objective: Residential addresses will be geocoded using StreetMap Premium Software in ArcGIS version 10.5.1 or later. Abstracted patient residential address information will be geocoded to assign each address a set of geographic coordinates. Completing this objective will yield coordinates that you will use to join addresses with US census tracts in Part 3b.

Step 1. Preparing to Geocode

Opening ArcMap and Naming and Saving the ArcMap Document that will Contain Geocoded Patient Residential Addresses

1. Open ArcMap by double clicking on the ArcMap icon that is pinned to the taskbar on the bottom of your Desktop.

   The ArcMap icon will look like this: 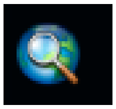

2. When you open ArcMap, the **ArcMap - Getting Started** dialogue box will appear in the center of your screen.

   Click on **New Maps.**

   An option to work with a Blank Map will be offered.

   Double click on the **Blank Map** icon under **My Templates.**

   Click **OK.**

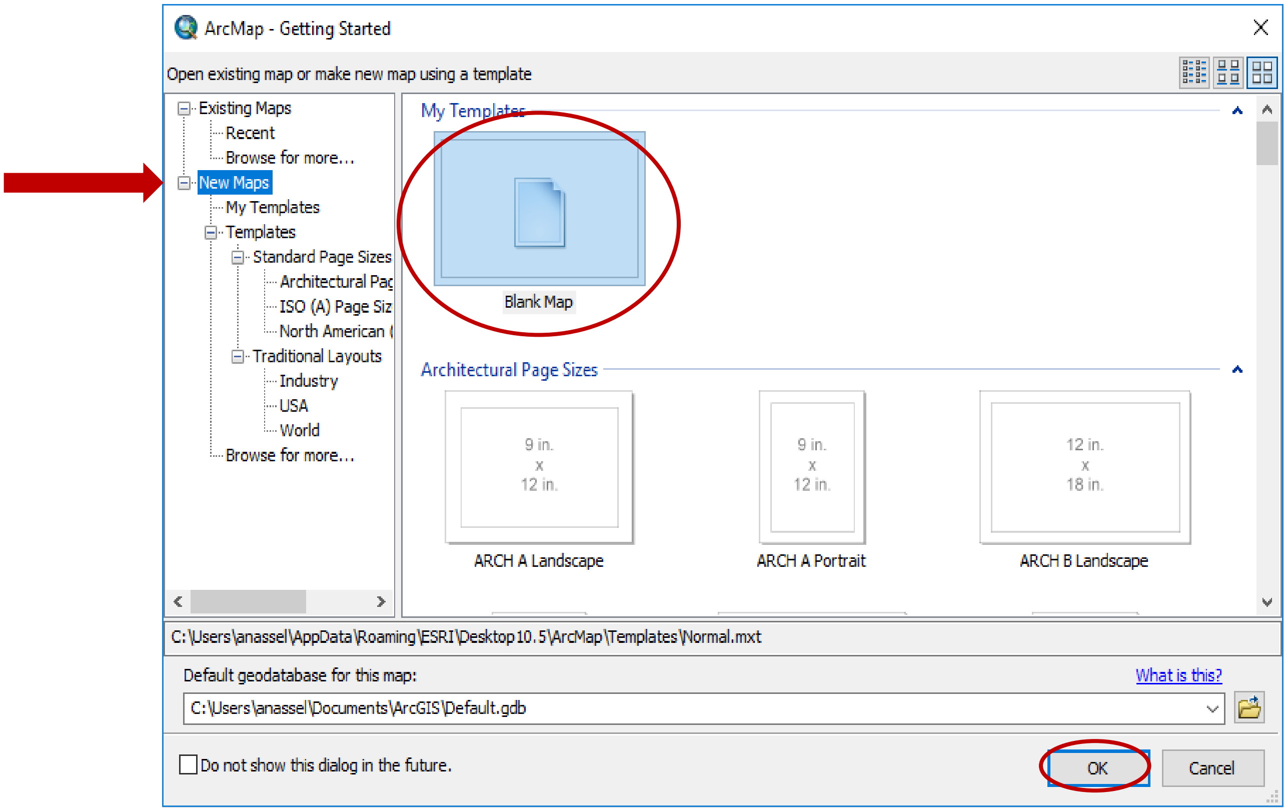

3. A blank **Untitled – ArcMap** screen will open and your screen should resemble the following image:

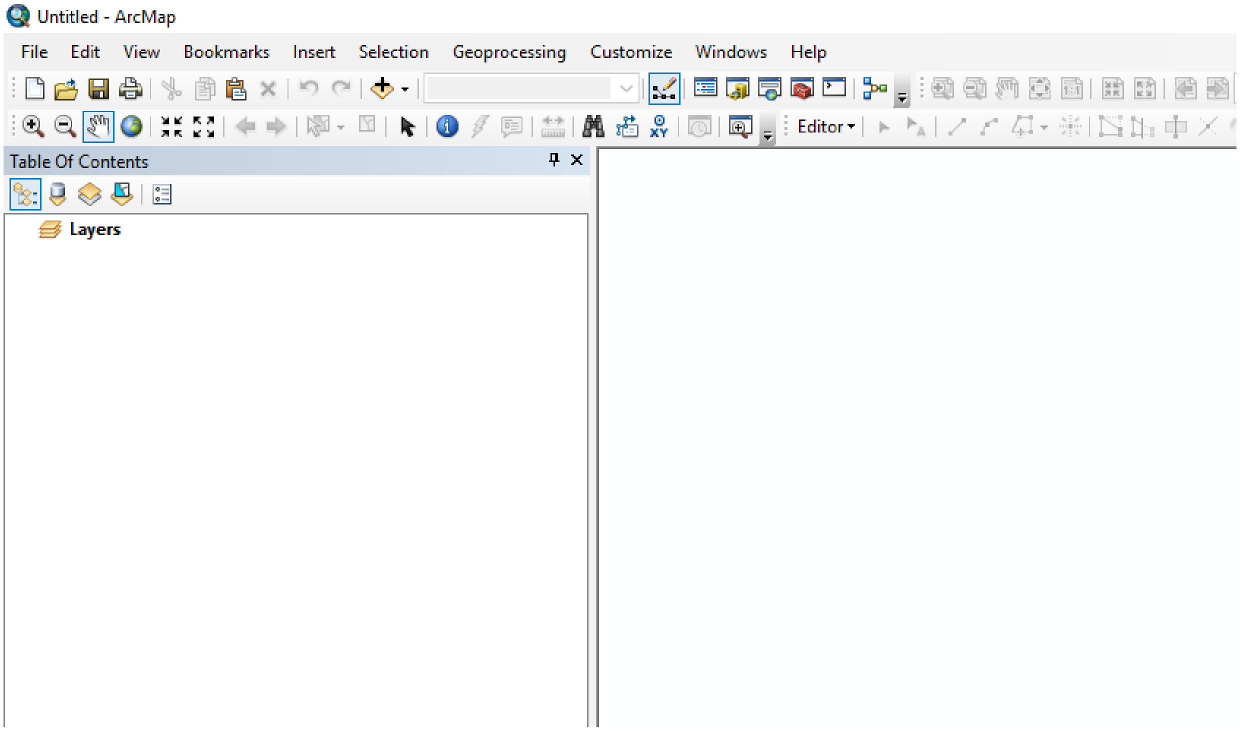

4. To name and save the new blank map that you created and that will eventually contain the geocoded residential addresses, open the **File** tab at the top of your screen and select **Save As.**

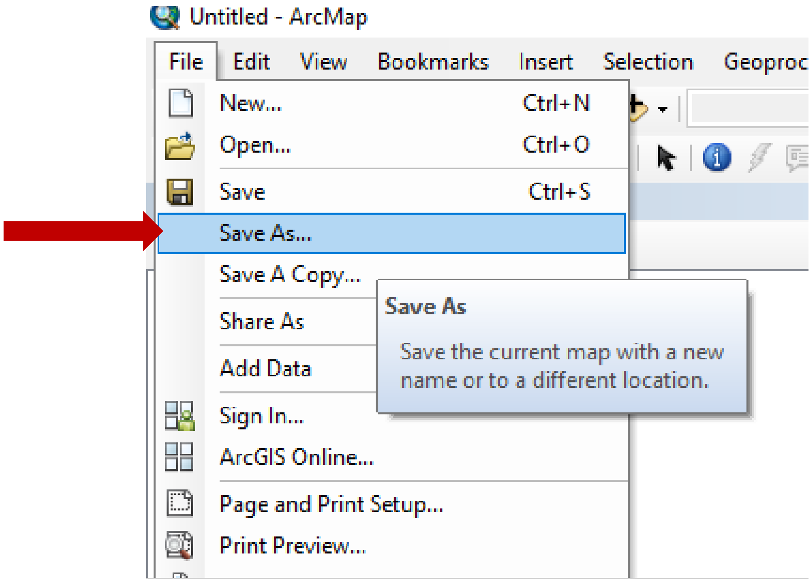

5. The **Save As** dialog box will open.

   Name the blank map in a format that is specific to your study and state. The name of the blank map should have the extension “**.mxd**”.

   Click **Save.**

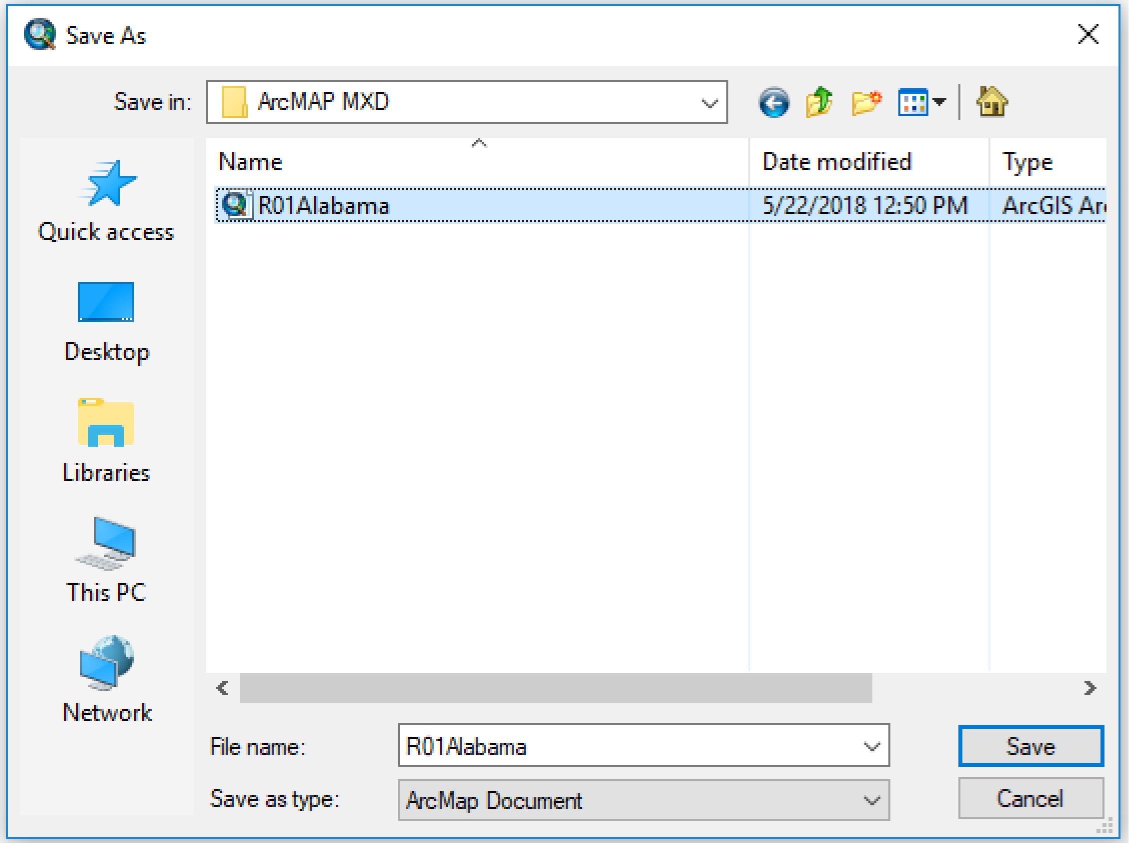

Step 2. Geocoding Residential Addresses

1. Once the **.mxd** file (e.g., R01Alabama.mxd) opens in ArcMap:

   Open the **File** tab and select **Add Data – Geocoding – Geocode Addresses**.

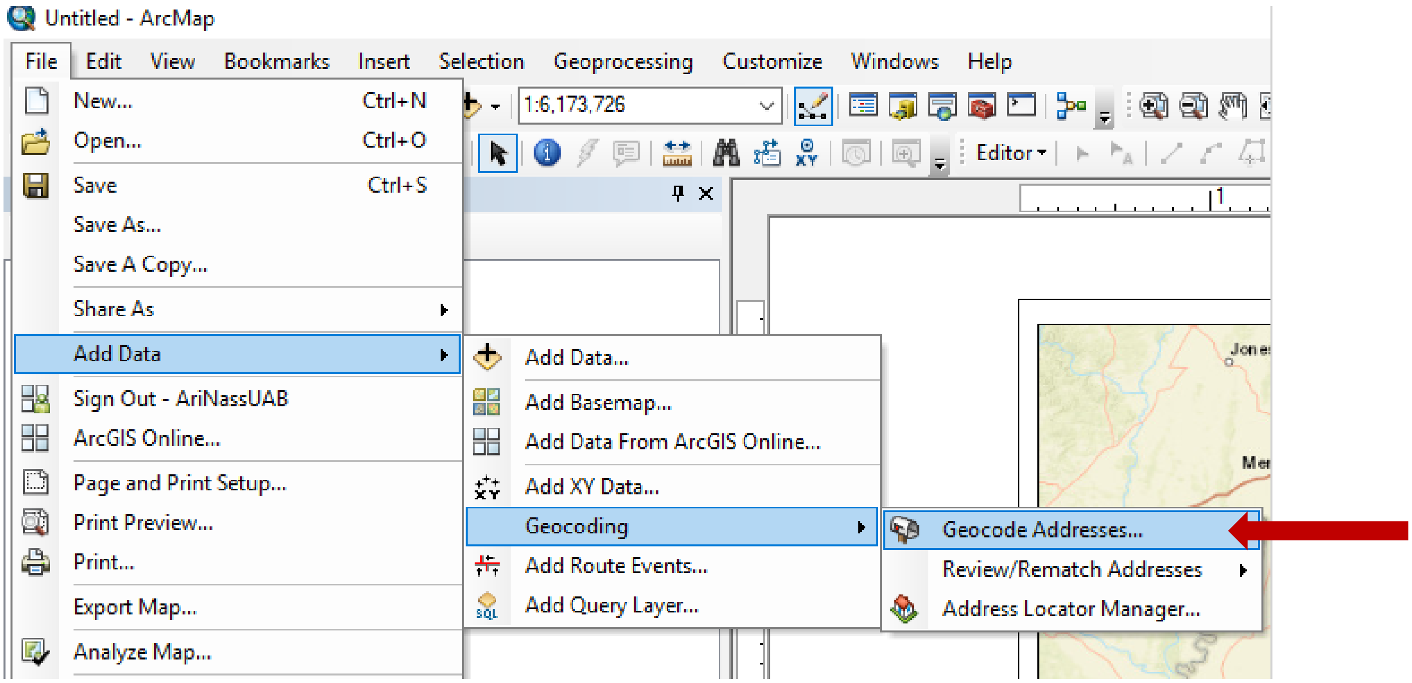

2. The **Choose an Address Locator to use** dialog box will open.

   Select **ArcGIS World Geocoding Service**.

   Click **Add.**

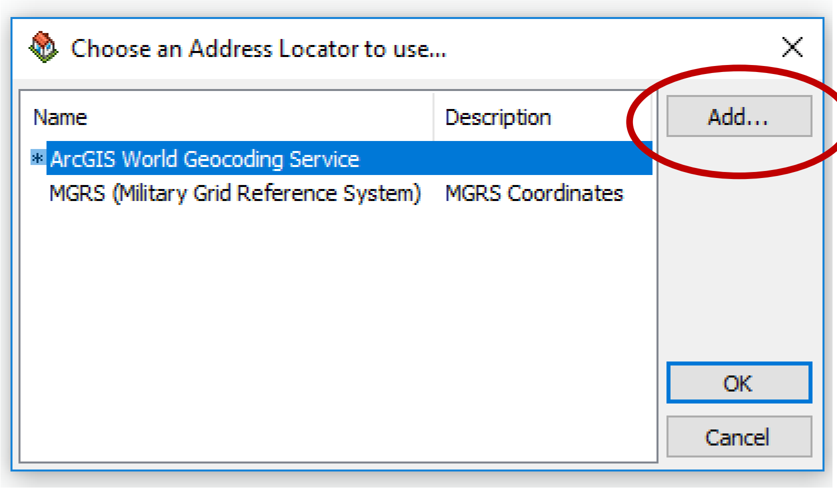

3. A list of available address locator boxes will open in the **Add Address Locator** dialog box.

   Select **USA_StreetAddress.**

   Click **Add.**

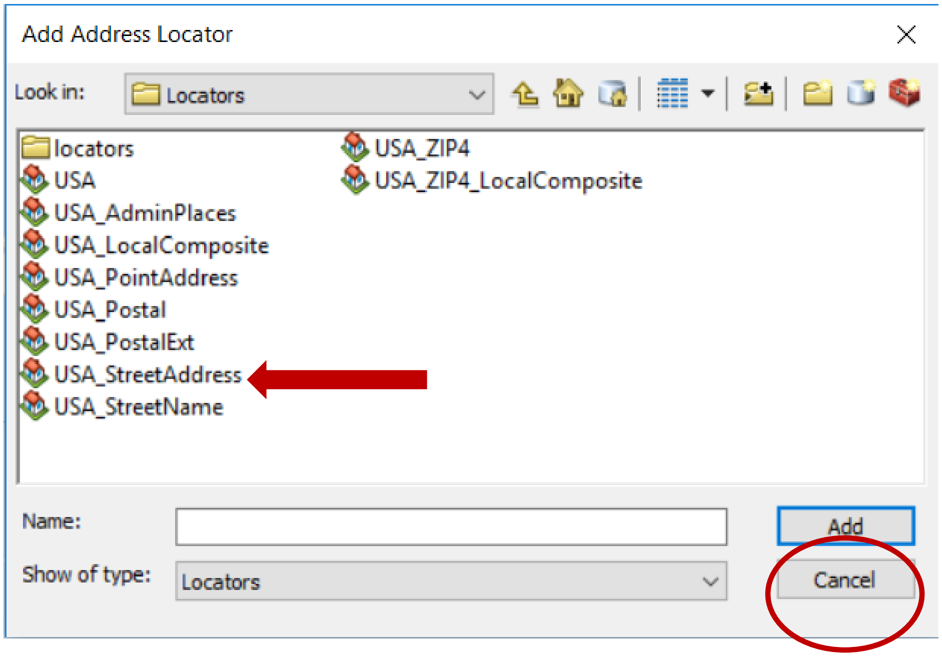

4. The **Geocode Addresses: USA_StreetAddress** dialog box will open.

   You will now add the **.csv** file that contains the residential patient addresses for all patients who are enrolled in your study. (See **Part 2: Preparing the File Containing Residential Addresses** of this protocol for information on the .csv file.)

   For this training, navigate to the folder that contains the **.csv** file of residential patient addresses.

   Click **OK.**

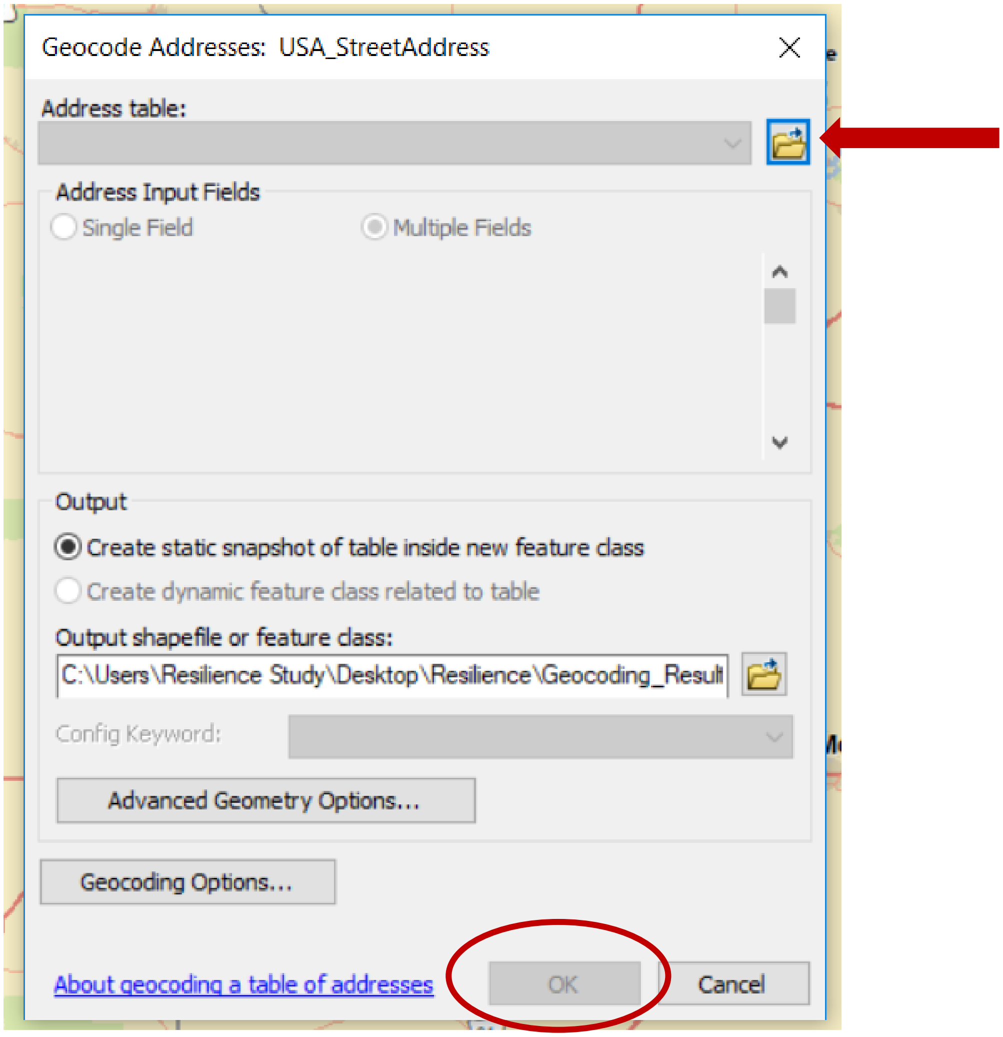

5. You will now have the option to choose the table (i.e., the .csv file) that contains the patient residential addresses.

   Note: Esri refers to .xls files and .csv files as "tables".

   Click once to highlight the .csv file that contains the patient residential addresses.

   Click **Add.**

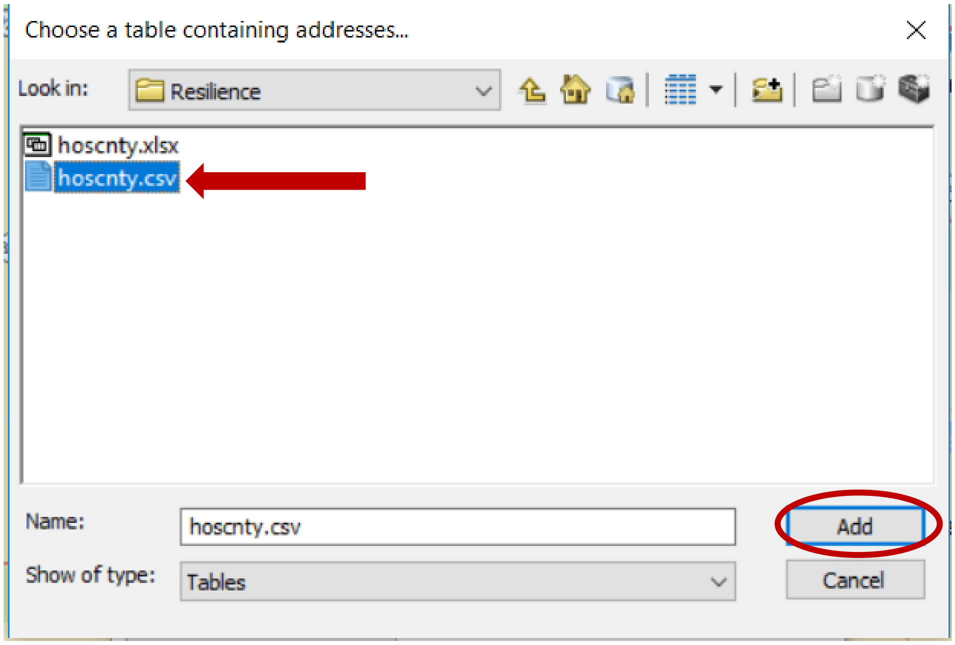

6. After adding the .csv file that contains the patients' residential addresses, the **Geocode Addresses: USA_StreetAddress** dialog box will reopen.

   Use the drop down menus to set each of the following variables to the correct field:

   The **Street or Intersection** field should be set to address.
   The **City or Place name** field should be set to city.
   The **State** field needs to be set to state.

   Your screen should match the below screenshot (for the fields indicated by the red arrows.)

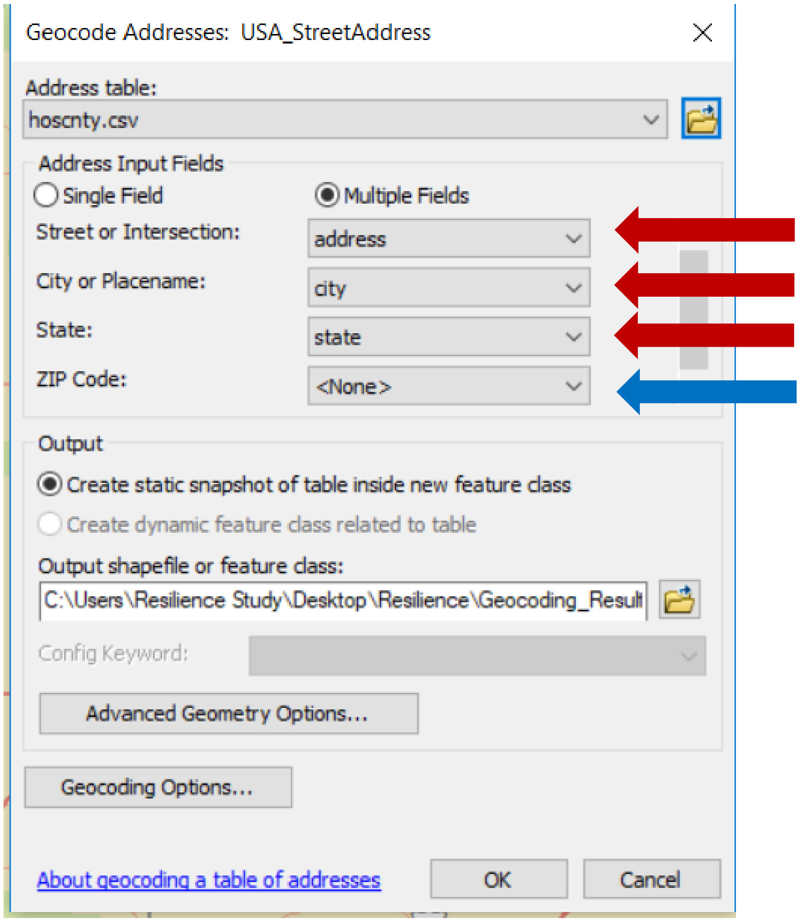

   The **ZIP Code** field (the blue arrow in the above screenshot points to this field) will most likely not be linked to the zip code variable. To link the **Zip Code** field to the proper variable, click on the dropdown menu and select **zipcode** from the drop down menu.

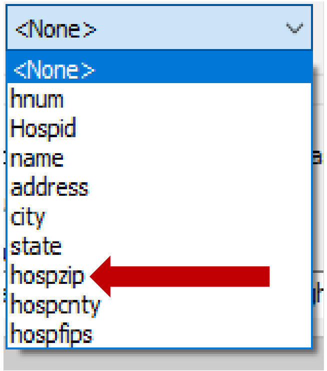

Zipcode will be added to the **ZIP Code** box, which ensures that ArcMap uses "zipcodes" from the .csv file that contains the patient residential addresses during the geocoding process.

7. Navigate to the folder where the output from the geocoding process will be stored and that is on your clinic desktop computer.

   In the **Output shapefile or feature class field:** click on the folder icon and navigate to the relevant output folder. This is the folder where the newly geocoded data file will be saved. After navigating to the folder, append the name of the file to this link so that the name clearly specifies that the file contains geocoded patient residential addresses (e.g., R01Alabalama_Geocoded).

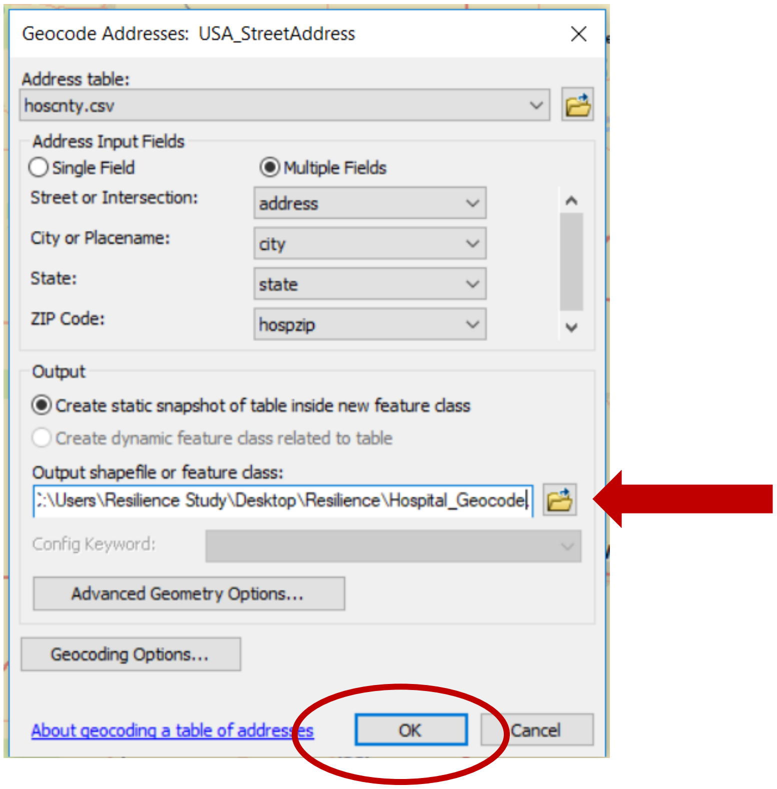

Click **OK.**

8. When you click **OK,** ArcMap will geocode the patient residential addresses that are in the .csv file. When the geocoding process is complete, a dialog box will open that states how many of the addresses "**Matched**", "**Tied**", or "**Unmatched**" (i.e., were not able to be geocoded).

    Note: It is common during geocoding that some addresses will be more difficult to geocode than others. To ensure that we are able to geocode as many addresses as possible, we can try re-geocoding the unmatched addresses.

To do this, click **Rematch**.

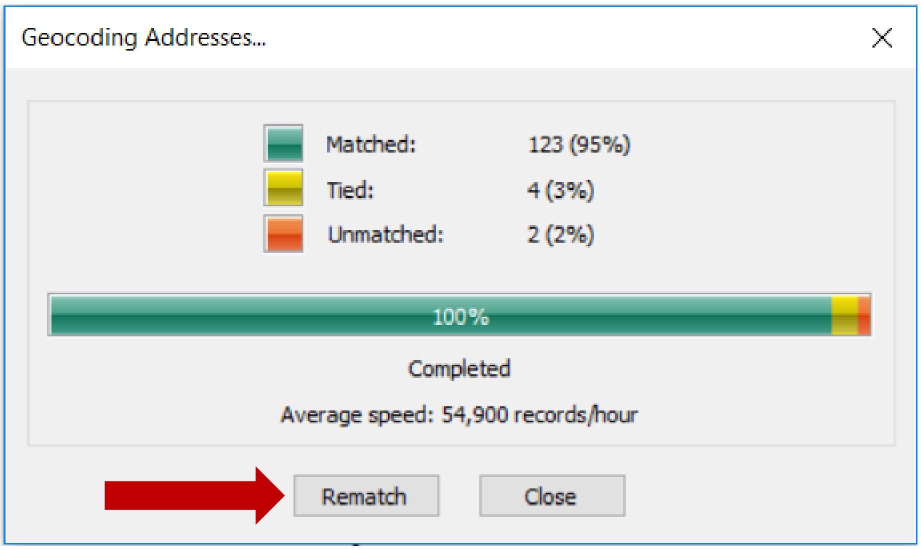

9. After you click **Rematch**, the **Interactive Rematch** dialog box will open.

   Click **Match** at the bottom of the dialog box to intiate the rematch.

   Click on each unmatched address under **Show results**, then at the bottom of the dialog box choose **Match** to initiate the rematch. You will be given a list of candidates in the middle dialog box that the software considers to be a possible match for the address that was not able to be geocoded. In the example below there are 4 candidates that are possible matches for the unmatched address that is (619 S 19th St M, Birmingham, AL. 35249).

   If there is a candidate with a Score of 85% or better, click on it and then click **Match**.

   If none of the candidates match, move to the next unmatched candidate and repeat to see if there is a matching candidate.

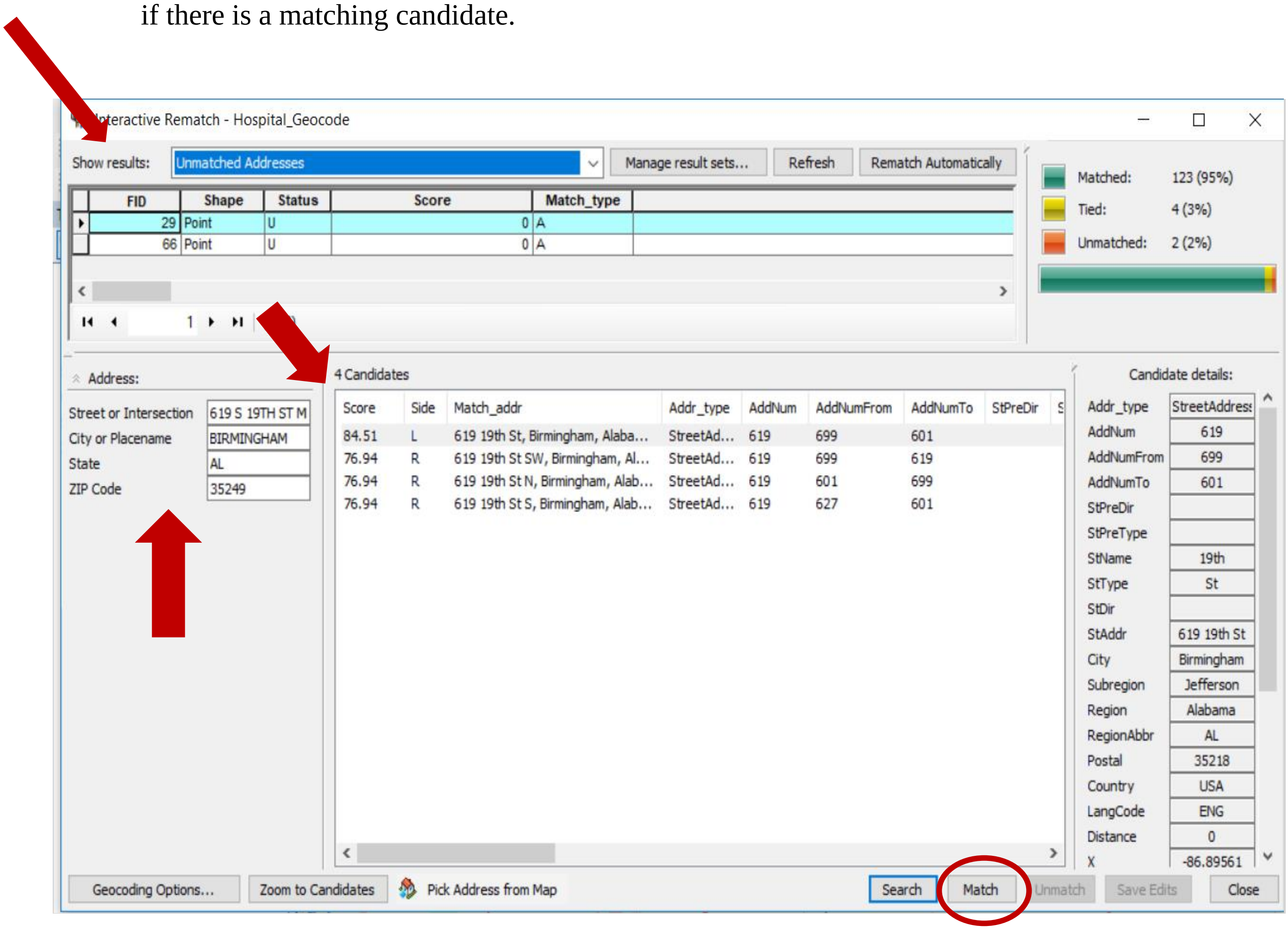

The following dialog box will appear to **Confirm Automatic Rematch Operations.**

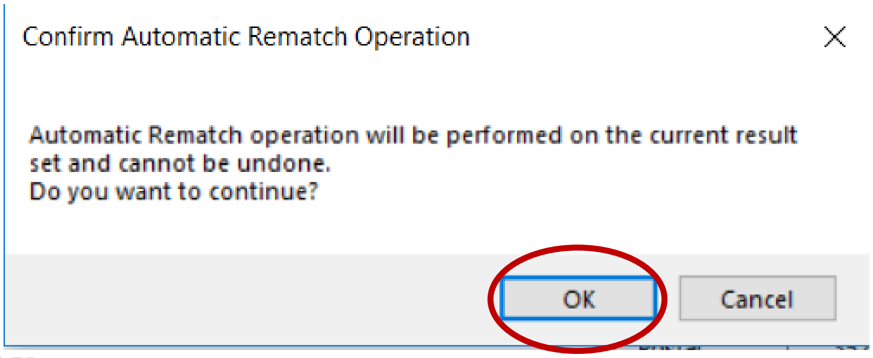

Click **OK.**

Some addresses may not be able to be geocoded. If this happens do not delete the Study ID. Each Study ID must be retained in the dataset and transferred to the coordinating institution. While an option is to use a search engine (e.g., GOOGLE Maps) to identify/verify street addresses, this approach is not secure and can create opportunities for non-research personnel to unlawfully access patients' protected health information (PHI). Please do not use this approach.

When completed click **Close**.

10. After the rematch is completed, you will see a layer of points on the map. Each of these points is the physical location of a patient's residential address.

11. To see where the addresses are located, add a basemap of streets, highways, and state boundaries.

    Click on the **Add Data** icon.

    Click on **Add Basemap**

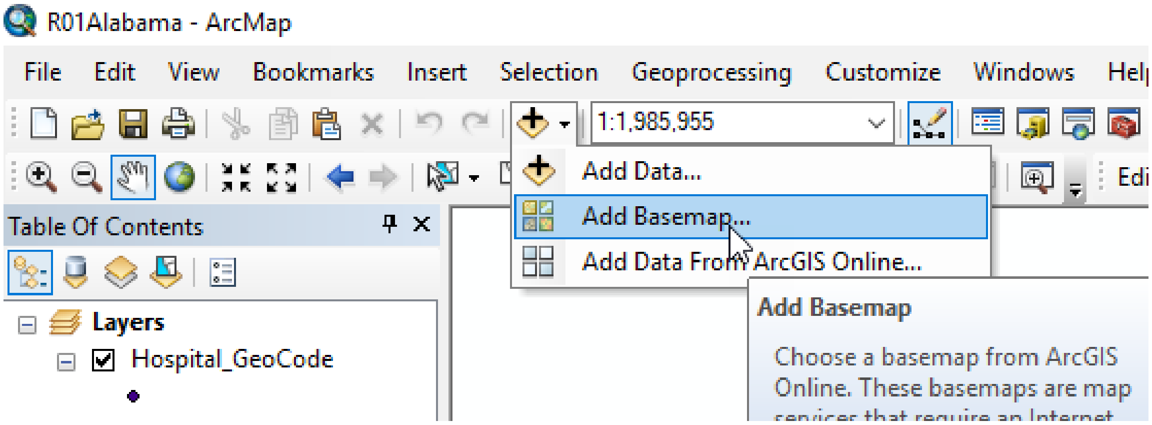

12. The **Add Basemap** dialog box will open with options for different basemaps.

    Double click on the **Streets** thumbnail. (This is the basemap for the entire map of streets and geographic boundaries in the United States.)

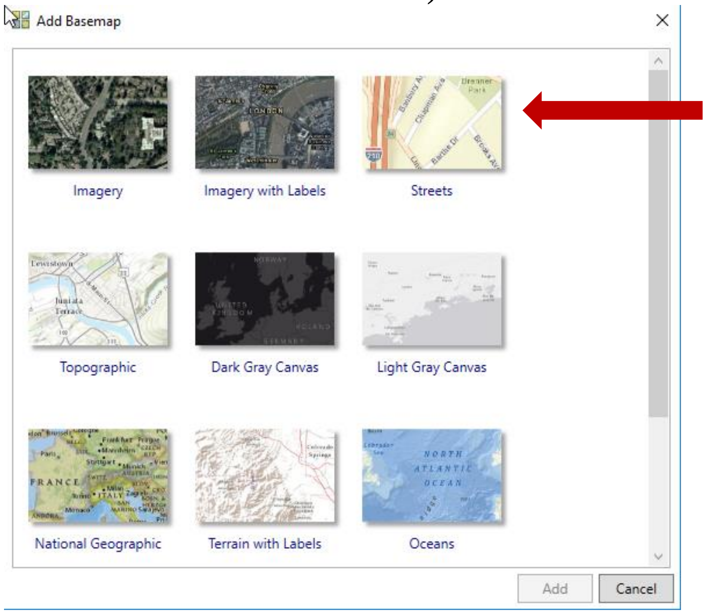

13. The street basemap will open in your layout window.

14. Zoom into your area of interest by double clicking on the state for which you are geocoding addresses. Your map will look similar to the screenshot below and you will see points on the map (black dots) that represent the patient residential address locations that were geocoded.

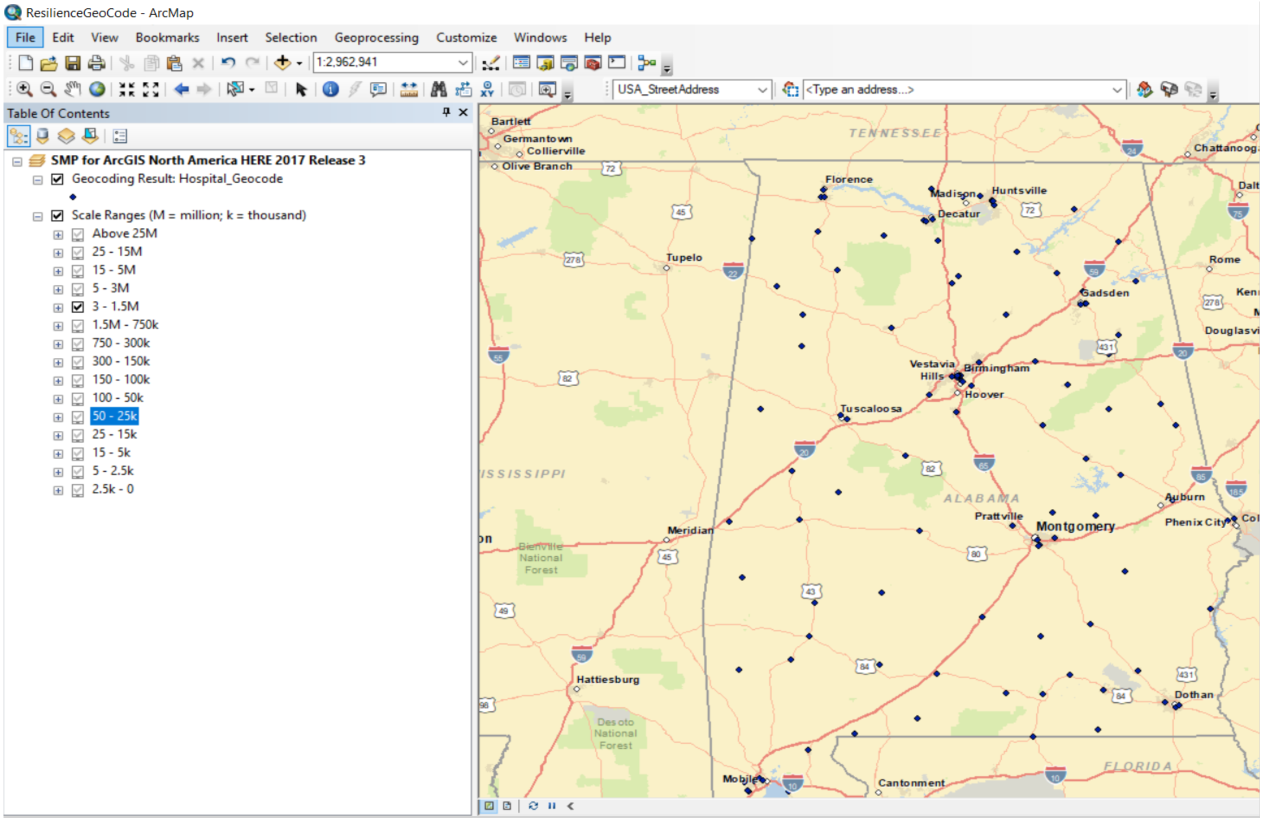

This was the last activity needed to geocode residential addresses. The next step, Part 3b, will instruct you on how to join geocoded address points to census boundaries.

Part 3b: Joining Geocoded Address Points to Census Boundaries

Program: StreetMap Premium Software purchased from Esri.

Objective: The next step in the protocol is to "**join**" the points that you have geocoded to the data layer with census tract boundaries. Completing this objective will yield census tracts that will be linked to US Census data in Part 4 and crime data in Part 5.

Step 1. Add the data layer containing the census tract boundaries to the MXD.

1. Click on the **File tab** that is located at the top left-hand corner of the screen.

   A drop down menu will open. Scroll down and hover your cursor over **Add Data.** This will open another drop down menu.

   Note: If a dialog box opens with the following warning: "Geographic Coordinate System Does not match", simply click **Close**.

   Click on **Add Data.**

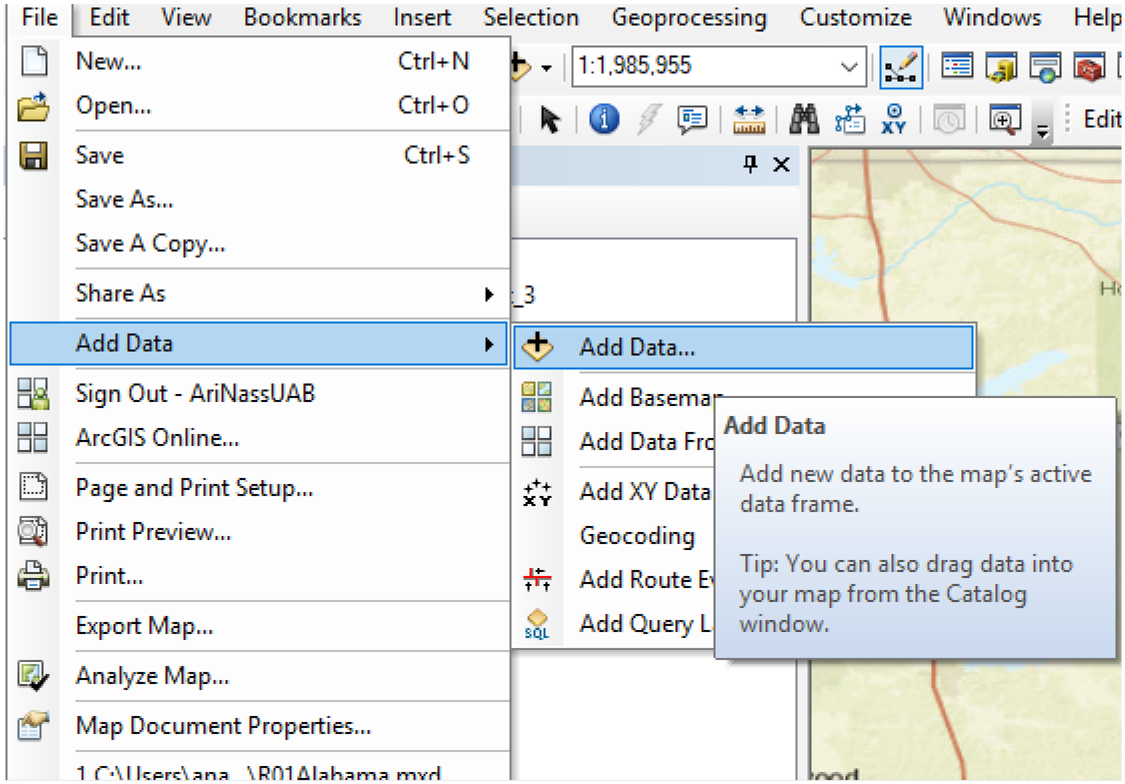

2. The **Add Data** dialog box will open.

   Use the Folder Connection icon to navigate to the relevant folder on your clinic desktop that contains the County and Tract Shapefiles for your state. These shapefiles will be available to you through the StreetMap Premium Esri extension.

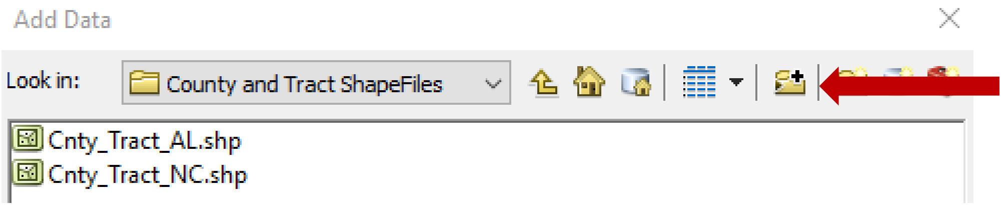

3. Inside the **census tract Shapefiles** folder, there is a shapefile for each state that contains all census tract boundaries in addition to the name of the county that each census tract is within.

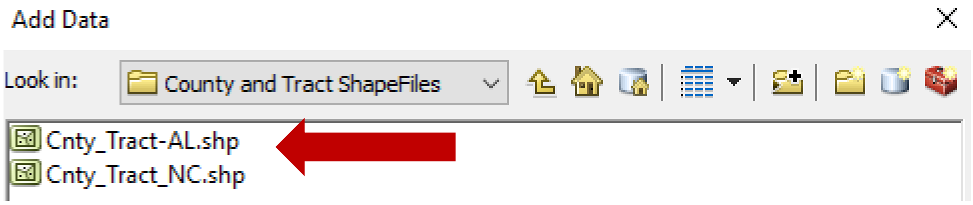

Note: The examples in this protocol use **Cnty_Tract_AL.shp**, which contains all counties and census tracts in Alabama

4. After adding the shapefile Cnty_Tract_AL.shp, the census tract boundaries will be displayed along with the layer of points that represent the geocoded addresses as displayed in the below example screenshot.

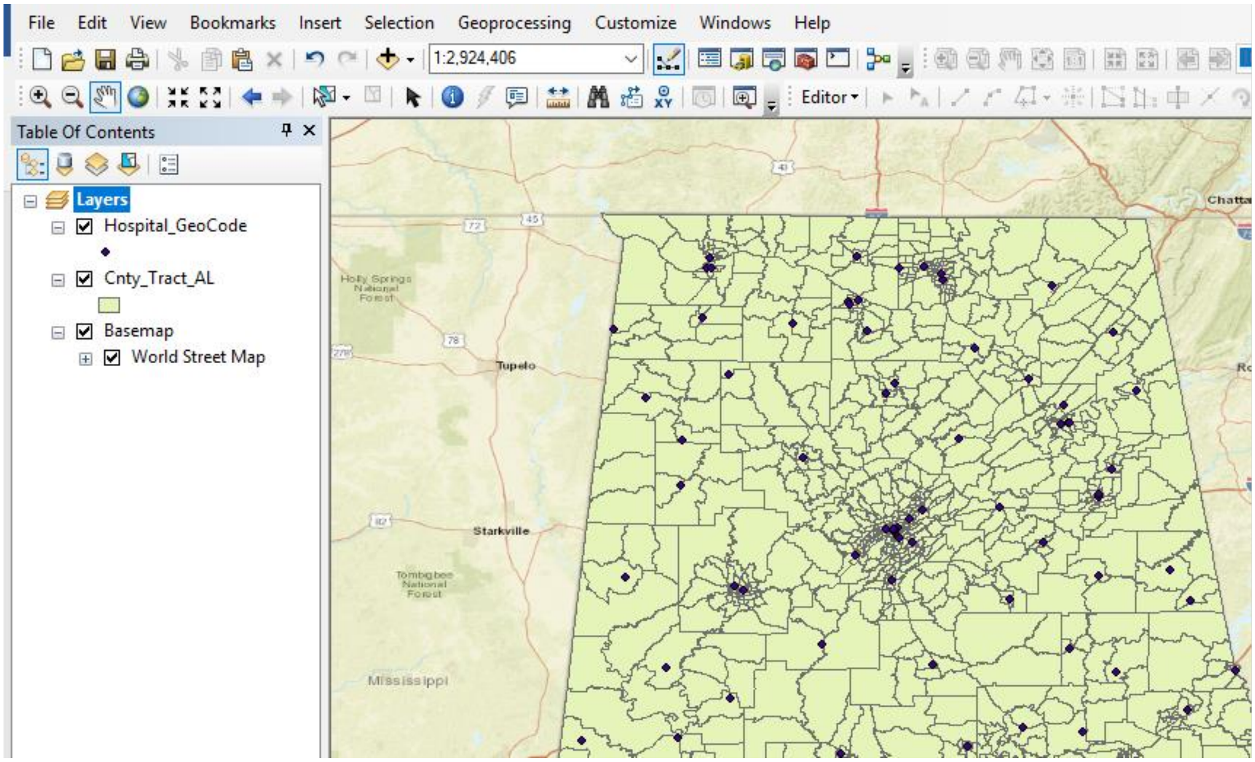

Step 2: Joining Geocoded Address Locations to Census Tracts

5. In the **Table of Contents** on the left side of your screen, locate the data layer that you just added that contains the census tract boundaries for your state. (In the below example screenshot, this data layer is called Hospital_GeoCode.)

   Right click on the layer name that contains the census tract boundaries.

   Scroll down to **Joins and Relates** and select **Join.**

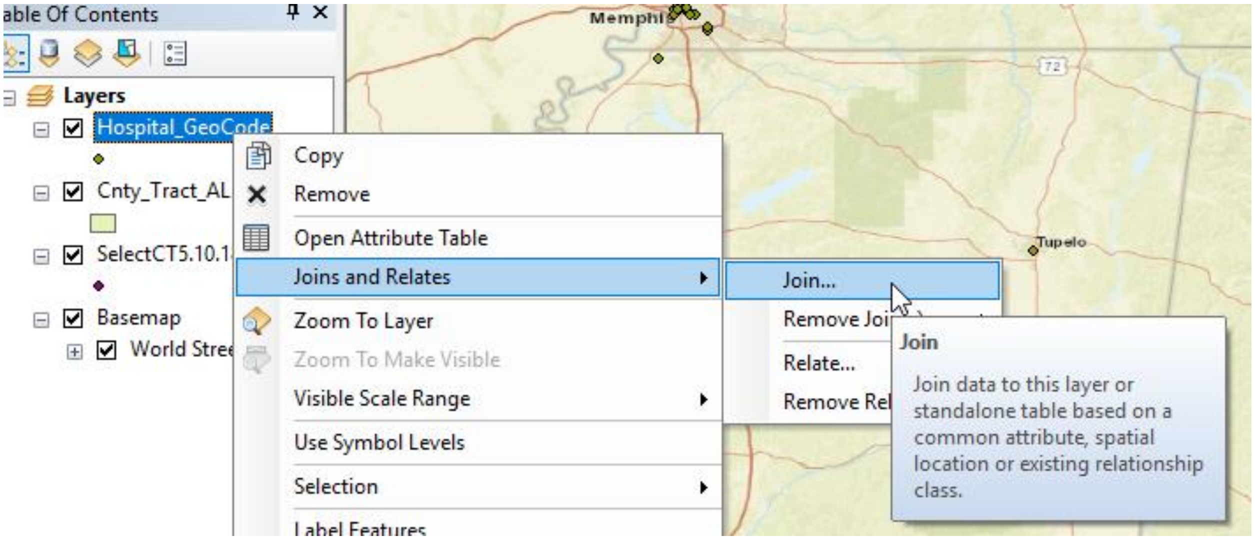

6. The Join Data dialog box will open.

You will see a dialog box that asks, **What do you want to join to this layer?**

- From the drop down menu, select **Join Data from another layer based on spatial location**.

Next, under **1. Choose the layer to join to this layer, or load spatial data from disk:**

- Click on the dropdown menu arrow and choose the data layer that contains the census tract boundaries for your entire state. (e.g., Cnty_Tract_AL).

Next, check that the dialog box that states **2. You are joining: Polygons to Points.** Make sure that **"it falls inside."** is toggled on.

Next, for **3. The result of the join will be saved into a new layer. Specify output shapefile or feature class for this new layer:**

- Click on the Folder Connection Icon and navigate to the output folder on your clinic desktop computer.

- Name the new file using the date the join was performed (e.g., **SelectCT.5.10.18**) by appending the name to the file location

- Click **OK.**

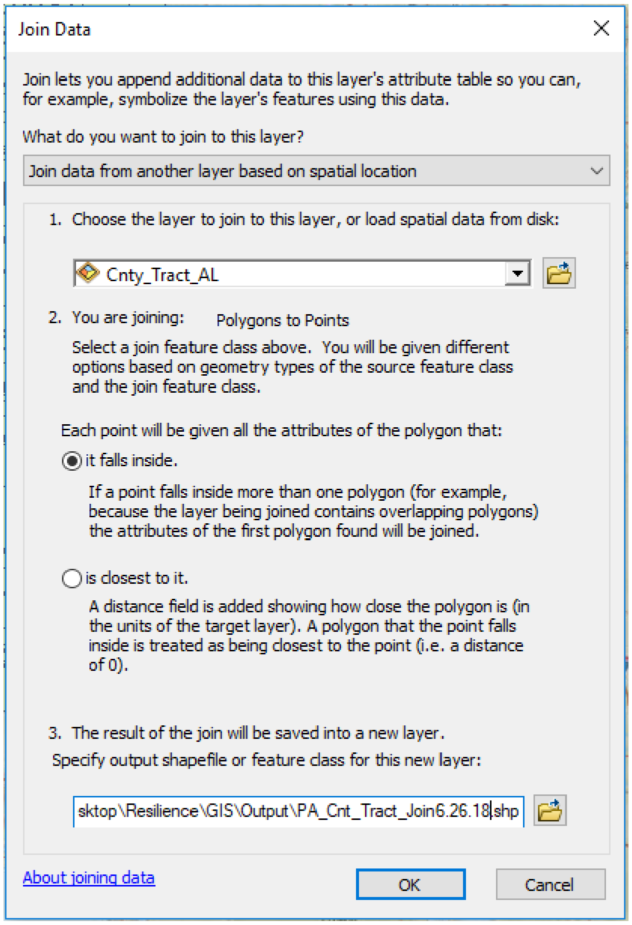

7. The newly joined layer will be added to the **Table of Contents** and will be visible in the data view screen.

   In the **Table of Contents**, the following three data layers will be shown. These three layers correspond to the data that are on your map**.**

**Data Layer File extensions**	**Data Layer File Extensions (Names used in GIS training)**	**Map Symbol**	**Description**
Resilience_GeoCode11.1.18	Hospital_GeoCode	Hospital_GeoCode	The geocoded layer of points that represent geocoded locations.
SelectCT.month.date.year	SelectCT.5.10.18	SelectCT.5.10.18	The data layer that contains **only** the census tracts that contains geocoded locations.
Cnty_Tract_Your State	Cnty_Tract_AL	Cnty_Tract_AL	The initial data layer that contains the census tract boundaries for the entire state.

Your screen will resemble the following image (or an approximate since you are using your clinic-specific data):

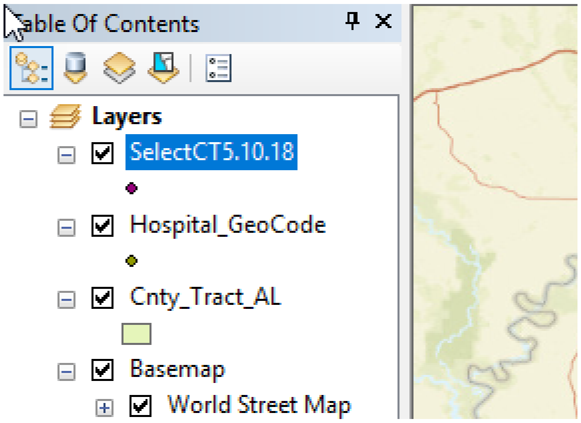

Step 3. Obtaining and formatting the joined census tract data

8. Right click on the Cnty_Tract_AL layer in the Table of Contents

   Left click on **Open Attribute Table**.

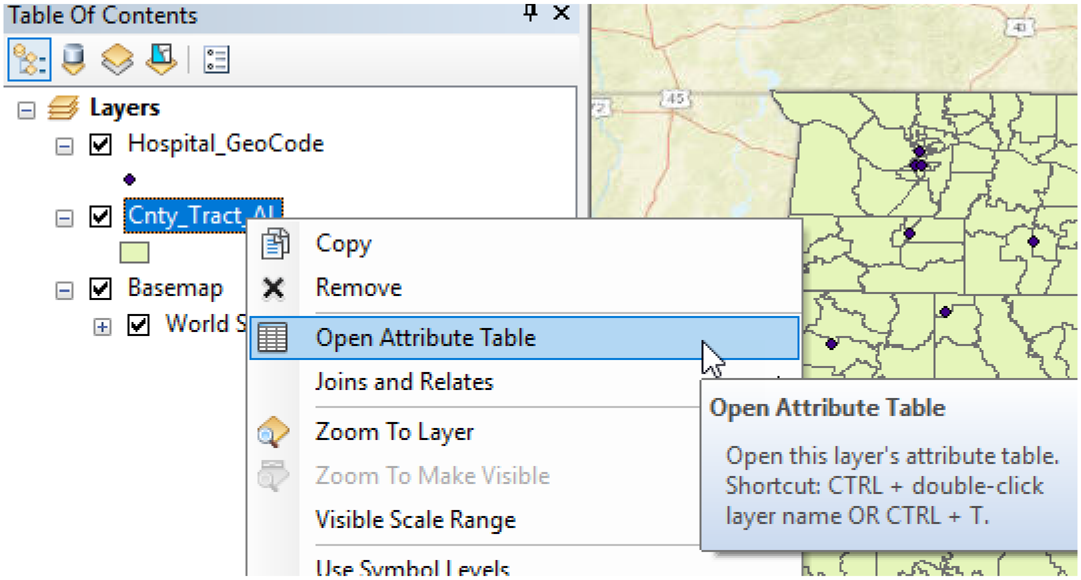

9. This will open a large table of data that resembles the screenshot below:

   Click on the small box in the upper right hand corner to **maximize** the table.

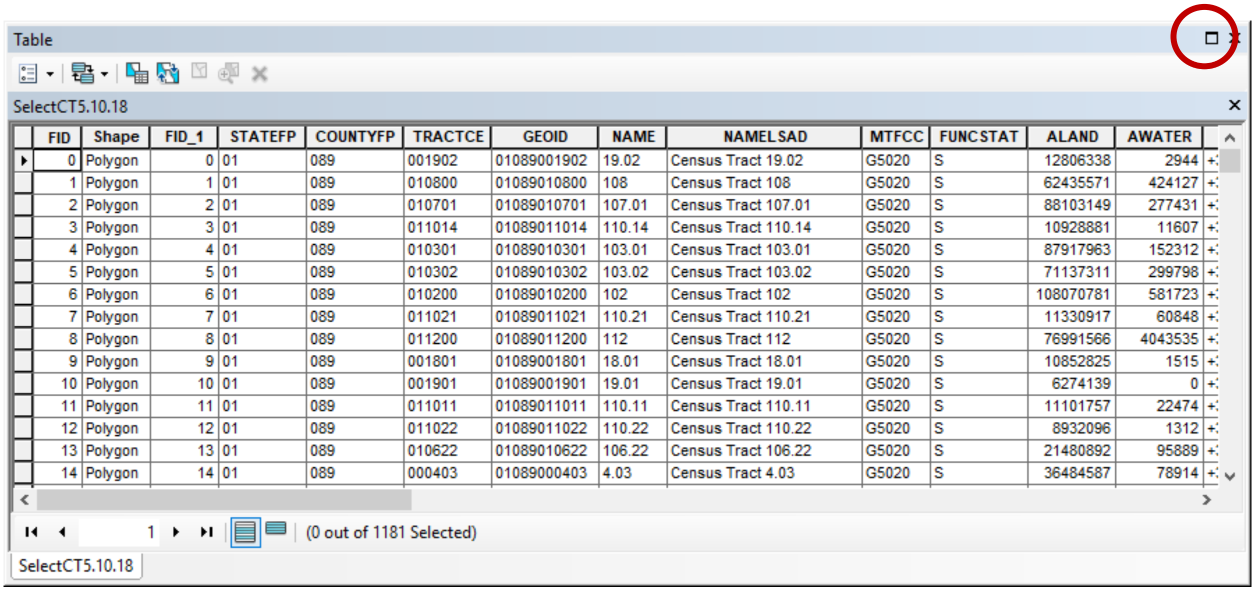

FID	Shape	FID_1	STATEFP	COUNTYFP	TRACTCE	GEOID	NAME	NAMELSAD	MTFCC	FUNCSTAT	ALAND	AWATER
0	Polygon	0	01	089	001902	01089001902	19.02	Census Tract 19.02	G5020	S	12806338	2944
1	Polygon	1	01	089	010800	01089010800	108	Census Tract 108	G5020	S	62435571	424127
2	Polygon	2	01	089	010701	01089010701	107.01	Census Tract 107.01	G5020	S	88103149	277431
3	Polygon	3	01	089	011014	01089011014	110.14	Census Tract 110.14	G5020	S	10928881	11607
4	Polygon	4	01	089	010301	01089010301	103.01	Census Tract 103.01	G5020	S	87917963	152312
5	Polygon	5	01	089	010302	01089010302	103.02	Census Tract 103.02	G5020	S	71137311	299798
6	Polygon	6	01	089	010200	01089010200	102	Census Tract 102	G5020	S	108070781	581723
7	Polygon	7	01	089	011021	01089011021	110.21	Census Tract 110.21	G5020	S	11330917	60848
8	Polygon	8	01	089	011200	01089011200	112	Census Tract 112	G5020	S	76991566	4043535
9	Polygon	9	01	089	001801	01089001801	18.01	Census Tract 18.01	G5020	S	10852825	1515
10	Polygon	10	01	089	001901	01089001901	19.01	Census Tract 19.01	G5020	S	6274139	0
11	Polygon	11	01	089	011011	01089011011	110.11	Census Tract 110.11	G5020	S	11101757	22474
12	Polygon	12	01	089	011022	01089011022	110.22	Census Tract 110.22	G5020	S	8932096	1312
13	Polygon	13	01	089	010622	01089010622	106.22	Census Tract 106.22	G5020	S	21480892	95889
14	Polygon	14	01	089	000403	01089000403	4.03	Census Tract 4.03	G5020	S	36484587	78914

10. You will copy and paste the data in this table to a new Excel worksheet.

    Open a new Excel document, name the file (e.g., **SelectedTracts5.10.18.xlxs**), and save the file to your clinic desktop computer.

    In the top left corner of the Attribute Table, left click the Table Options tab.

    Click **Select All**.

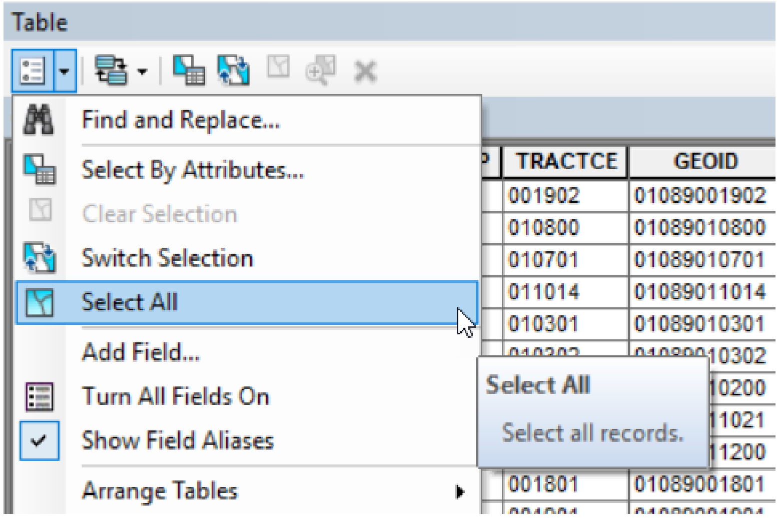

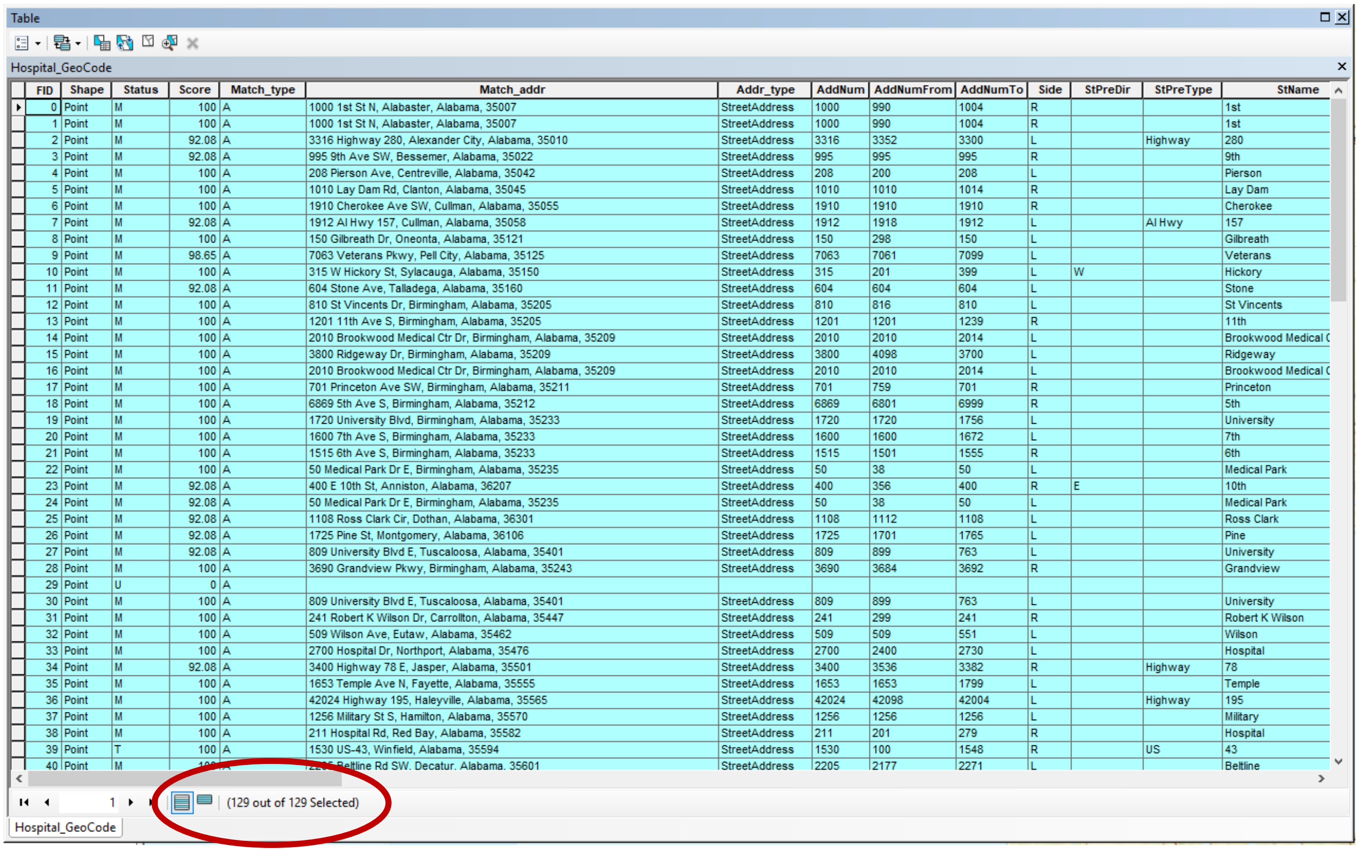

Your last row of data will be at the bottom of the Attribute Table. In this example, you will see that for Alabama 129 rows out of 129 rows of data have been selected.

11. You will now copy the selected rows of data into the **SelectedTracts5.10.18** Excel file that you just created.

    Hover the cursor over any row in the first column and right click.

    Click **Copy Selected**.

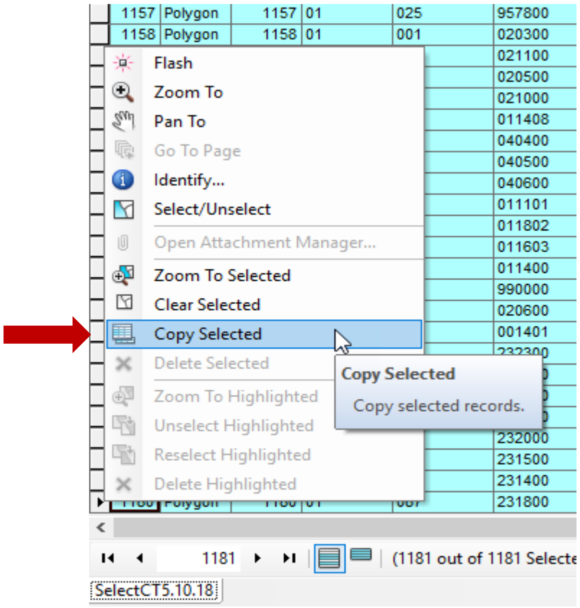

12. Paste the selected data rows into the blank **SelectedTracts5.10.18** Excel file.

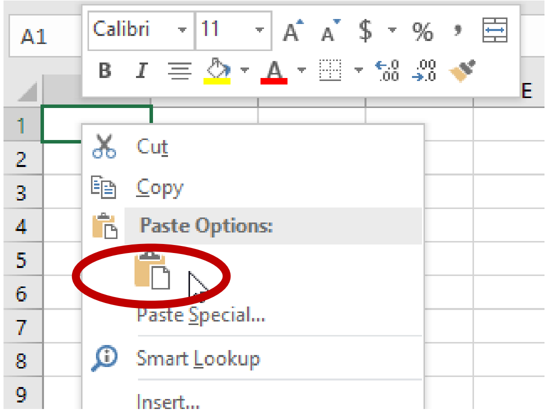

In this example, columns A through BY will contain data (e.g., 76 columns of data.)

13. The majority of the data that you pasted from the Attribute Table in ArcMap to the Excel file is not needed for this project. To make the file easier to work with you will delete the unnecessary data fields.

To make sure that you are deleting the correct data:

- **Highlight** the columns you see in the below table in Yellow in the excel file:

**Excel Column Letter:**	AM	AX	AY	AZ	BK	BM
**Excel Column Header:**	REDCap_ID	GEOID	NAME_1	NAMELSAD	GEOID10	NAMELSAD10
**Description of data included in column:**		Full Census tractIdentifier	Brief Census tractID	Census Tract	Census County ID	County

- **Delete** all columns that are not highlighted.

- After deleting the unneeded columns, 6 columns of data (Columns A – F) will remain.

For study data that are collected solely at a single time point, your screen should resemble the below example screenshot:

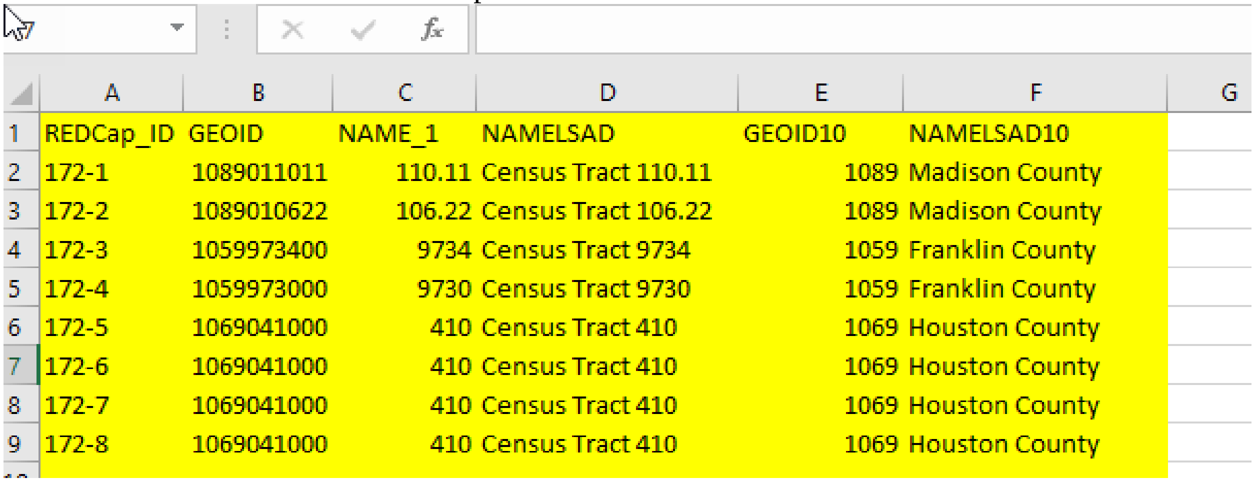

	A	B	C	D	E	F	G
1	REDCap_ID	GEOID	NAME_1	NAMELSAD	GEOID10	NAMELSAD10	
2	172-1	1089011011	110.11	Census Tract 110.11	1089	Madison County	
3	172-2	1089010622	106.22	Census Tract 106.22	1089	Madison County	
4	172-3	1059973400	9734	Census Tract 9734	1059	Franklin County	
5	172-4	1059973000	9730	Census Tract 9730	1059	Franklin County	
6	172-5	1069041000	410	Census Tract 410	1069	Houston County	
7	172-6	1069041000	410	Census Tract 410	1069	Houston County	
8	172-7	1069041000	410	Census Tract 410	1069	Houston County	
9	172-8	1069041000	410	Census Tract 410	1069	Houston County	

For study data that are collected at multiple time points, your screen should resemble the below example screenshot:

REDCAP_ID	GEOID	NAME_1	NAMELSAD	GEOID10	NAMELSAD10
172-1	1001020300	203	Census Tract 203	1001	Autauga County
172-2	1003010500	105	Census Tract 105	1003	Baldwin County
172-2	1003010500	105	Census Tract 105	1003	Baldwin County
172-3	1003010705	107.05	Census Tract 107.05	1003	Baldwin County
172-3	1003010705	107.05	Census Tract 107.05	1003	Baldwin County
172-3	1007010004	100.04	Census Tract 100.04	1007	Bibb County
172-4	1003010800	108	Census Tract 108	1003	Baldwin County
172-4	1003010800	108	Census Tract 108	1003	Baldwin County
172-4	1009050101	501.01	Census Tract 501.01	1009	Blount County
172-4	1009050101	501.01	Census Tract 501.01	1009	Blount County
172-5	1003011202	112.02	Census Tract 112.02	1003	Baldwin County

Understanding your census tract data:

Each row of data corresponds to each unique object in the shapefile. Each row of data contains

1) the participant's Study ID (e.g., REDCap_ID)
2) the long version of the Geoid (geoidentifier)
3) Census tract number (Name_1)
4) Restated census tract number (NAMELSAD)
5) Shortened version of the geoidentifer (GEOID10)
6) The County the Census tract is in (Namelsad10)

For Example, in the following screenshot,

Row 3 contains data for Study ID 172-2, Census tract106.22 in Madison County, Alabama.

REDCap_ID	GEOID	NAME_1	NAMELSAD	GEOID10	NAMELSAD10
172-1	1089011011	110.11	Census Tract 110.11	1089	Madison County
172-2	1089010622	106.22	Census Tract 106.22	1089	Madison County
172-3	1059973400	9734	Census Tract 9734	1059	Franklin County
172-4	1059973000	9730	Census Tract 9730	1059	Franklin County
172-5	1069041000	410	Census Tract 410	1069	Houston County
172-6	1069041000	410	Census Tract 410	1069	Houston County
172-7	1069041000	410	Census Tract 410	1069	Houston County
172-8	1069041000	410	Census Tract 410	1069	Houston County

In this example, your worksheet now has 6 columns and 129 rows.

However, the majority of the census tracts will not contain any geocoded address locations. Therefore, we will want to identify and save only those census tracts that contain at least 1 geocoded address.

Step 4. Identifying the counties that contain addresses geocoded to census tracts

In the next section**, Part 4** of this protocol, you will be abstracting crime data for specific counties.

Note: There is a high likelihood that not all counties in your state will have a patient residing in them. To save time you will only pull data for the counties that have patient addresses located in them. Data are abstracted for counties due to Esri formatting of Business Analyst data.

14. To determine which Counties contain at least one geocoded address location:

- **Highlight** rows A through F of your Excel file (named **SelectedTracts5.10.18** in this example).
- Right click on Column F and select **copy**.
- **Paste** columns A-F to a new worksheet in the same Excel file.
- In the new worksheet **highlight** Column F.
- Left click on Sort & Filter (located on the top right-hand corner of the Excel ribbon.
- Select **Sort A to Z.**

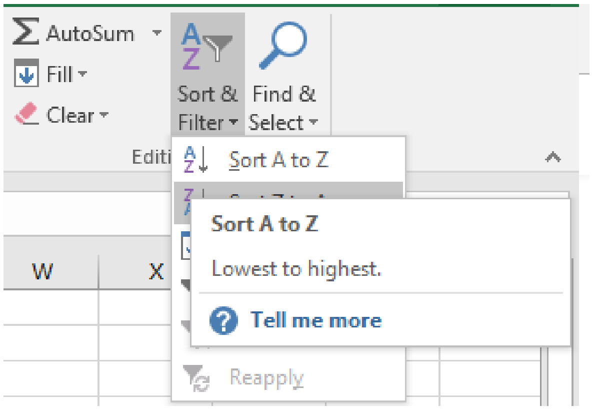

- The Sort Warning dialogue box will open.
- Leave the default choice of **Expand the selection.**
- Click **Sort.** This will sort the data alphabetically using the county name.

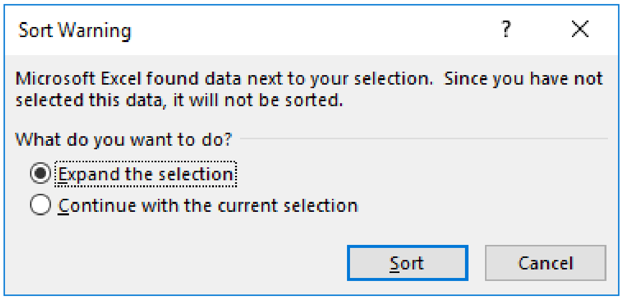

15. Your worksheet should now look similar to the screenshot below:

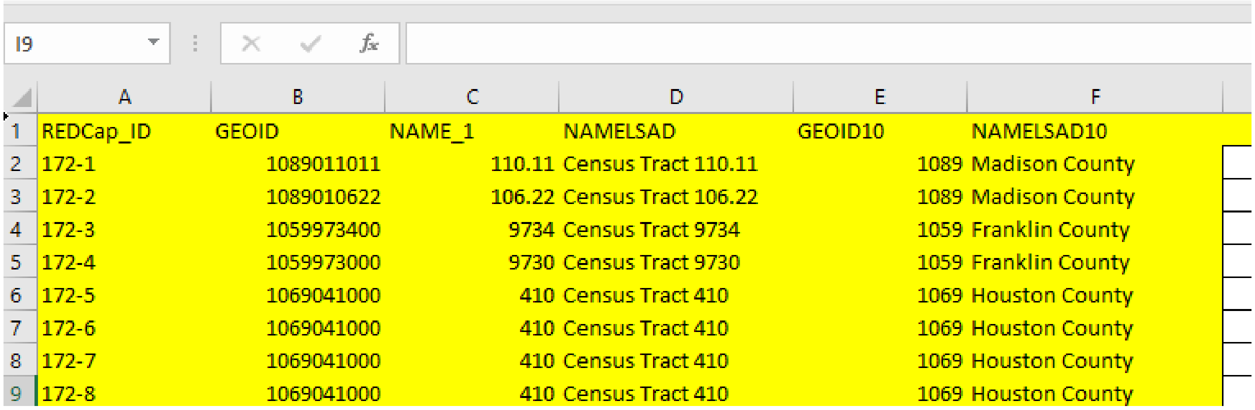

16. To create a list of the individual counties that contain at least geocoded address, you will use a Pivot Table.
    - Click on the H1 cell that contains a blank column.
    - In the top left hand corner of the Excel ribbon, select the **Insert** tab.
    - Double click the **Pivot Table** icon.

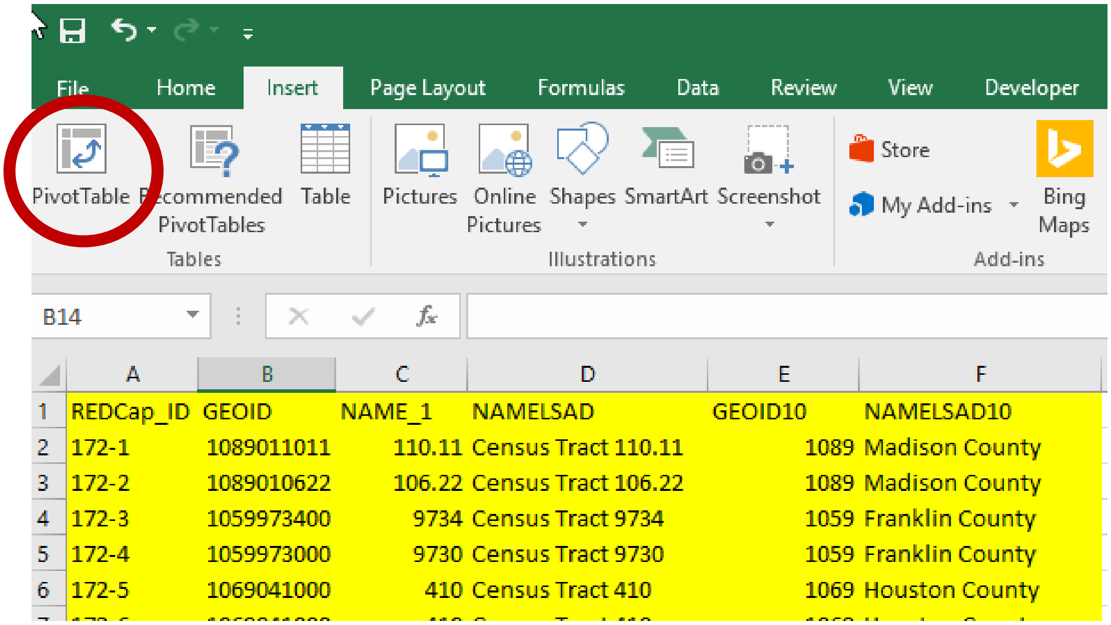

17. A **Create PivotTable** dialogue box will open.

To select the data that you want to be included in the pivot table:

- Left click on cell A1 and scroll across to Cell F and down to Cell F130. This will select all of the data that are in your worksheet.

- Click **OK.**

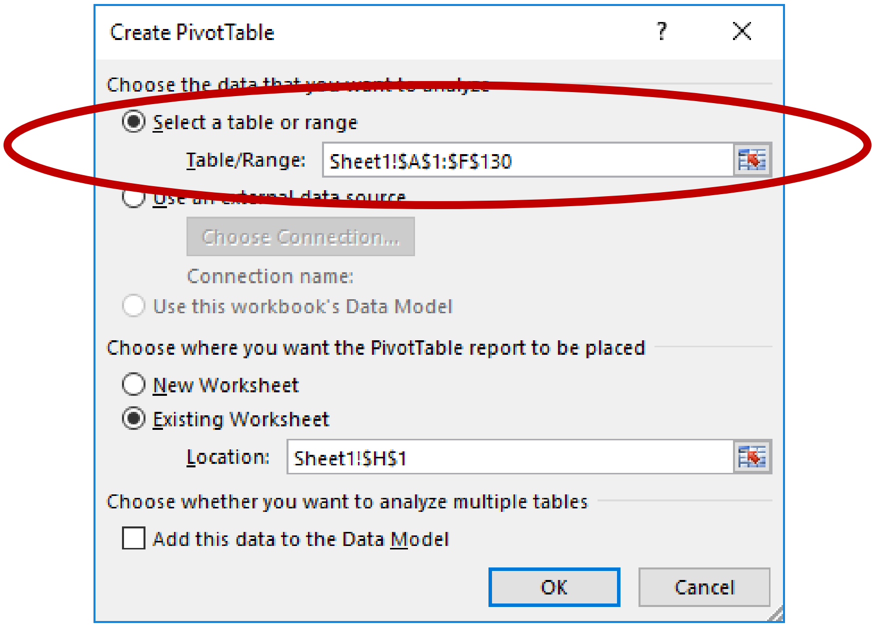

18. A List of Pivot Table Fields will open on the far right side of your worksheet

- Select the **NAMELSAD10** field.

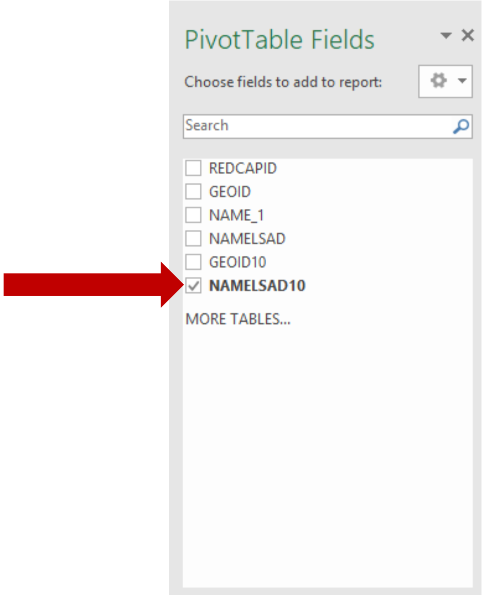

19. A list of the individual counties in the dataset will be displayed in Column H. Your screen should look similar to the following below screenshot:

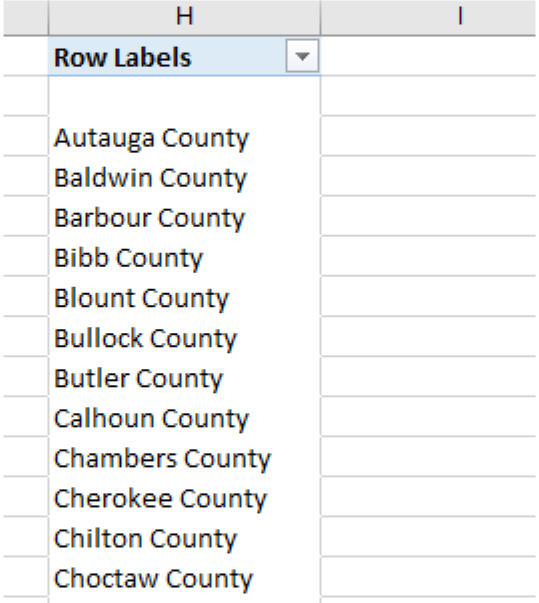

Note: **Print and/or save the list of counties into a separate word document so that you can refer to it later in the protocol.**

You will use this list when you abstract crime data as described in **Part 4** of this protocol. Each county listed will require a separate data pull.

- Rename the Excel file to indicate that the data were reformatted (e.g., **SelectedTracts5.10.18rf**).
- Save and close the document.

The data in this file will be merged into a final master Excel file as described in Part 6 of this protocol.

This was the last activity needed to identify the census tract and county that each residential address is within. This information will be used to link the individual census tracts with the crime data (Part 4) and socioeconomic data (Part 5).

Part 4: Obtaining Crime Data

Data Source: Esri Business Analyst Online Version 5.82 or later.

Objective: You will link the geographic information obtained from Part 3 with Esri Business Analyst crime data for patients. In this example, the crime data that will be abstracted from Esri Business Analyst include the 1) Murder Rate Index and 2) Assault Rate Index. Completing this activity will yield neighborhood-level crime data for census tracts in counties where patients reside.

Source(s):	Example Crime Variables
Esri Business Analyst Online	Murder Rate Index<br>Assault Rate Index

Step 1. Accessing Esri Business Analyst Online (BAO)

1. To access Esri Business Analyst, open a web browser and type **Esri Business Analyst Online** into the search box and hit enter. The browser will present results that will take you to the following login page.

   https://bao.arcgis.com/esriBAO/index.html#

   The **Log into Business Analyst** page will resemble the following.

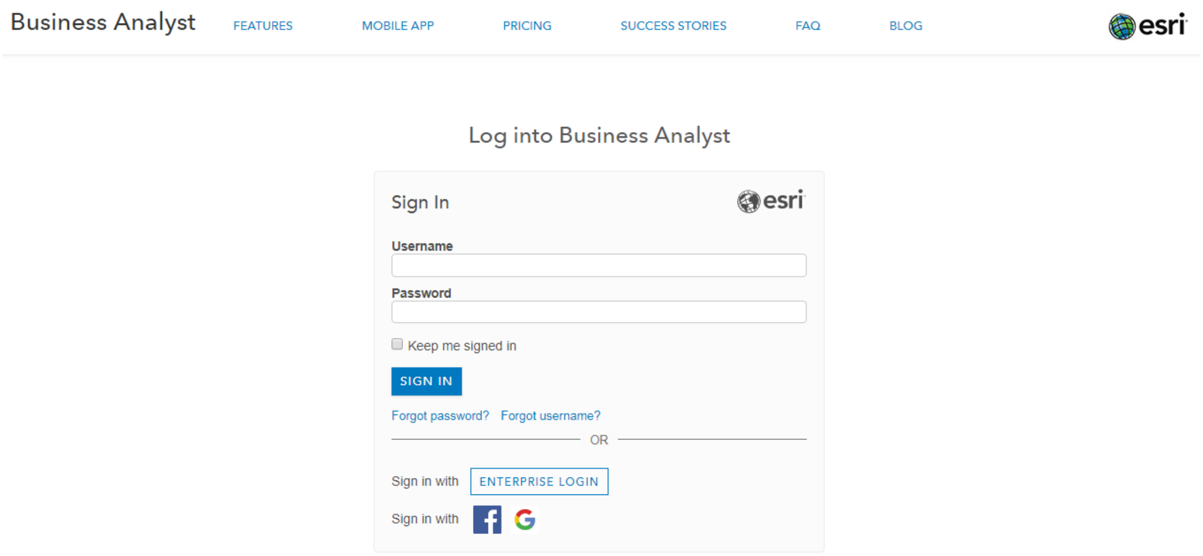

   Enter your **Username** and **Password**.

Step 2. Obtaining Crime Data from BAO

1. Once you are logged into Esri Business Analyst Online you will see the following screen with a green ribbon under Home.

   Note: The home page has a rolling banner so you may have a different shaded image on your screen.

   Click on **Get started Now**.

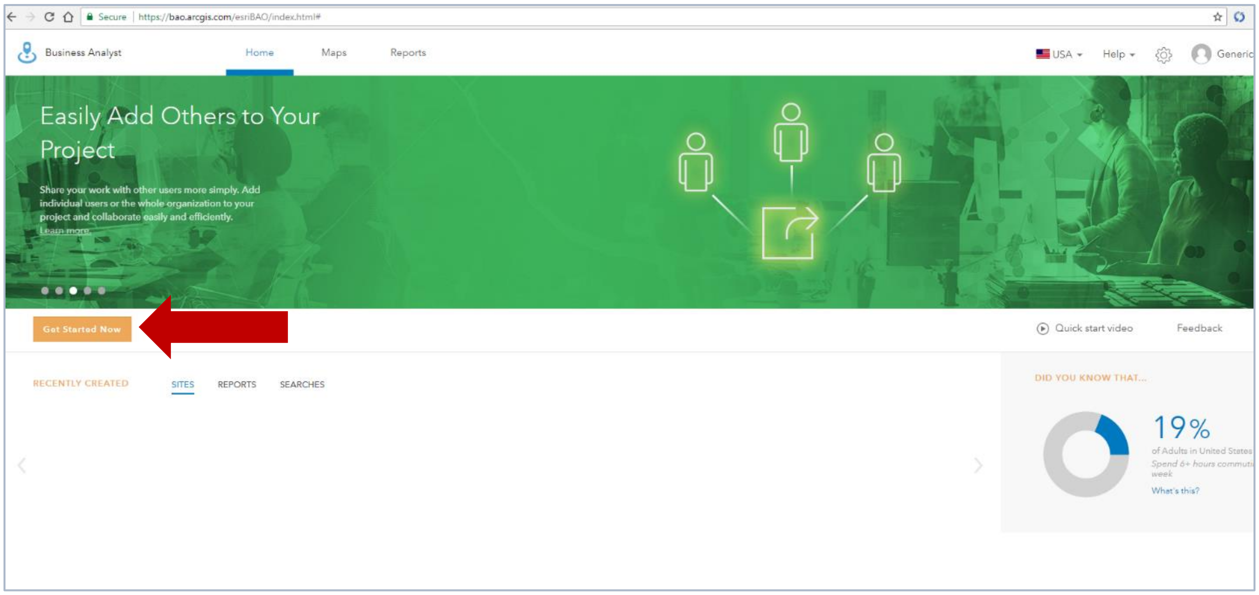

2. Create a project by clicking on **Create Project** in the center or top right hand corner of the Projects page.

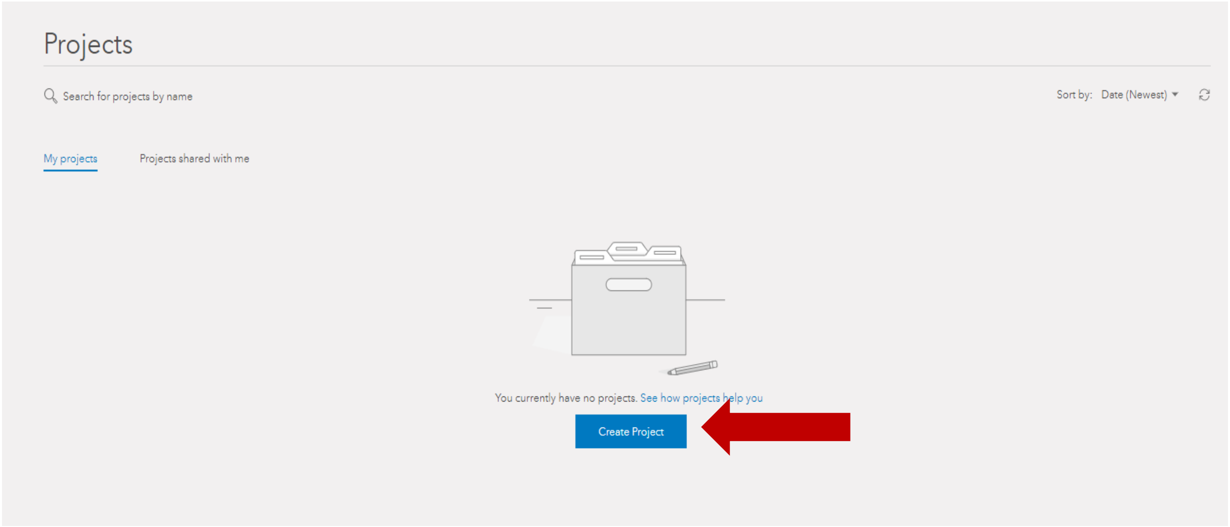

3. Name your project and select **Create**.

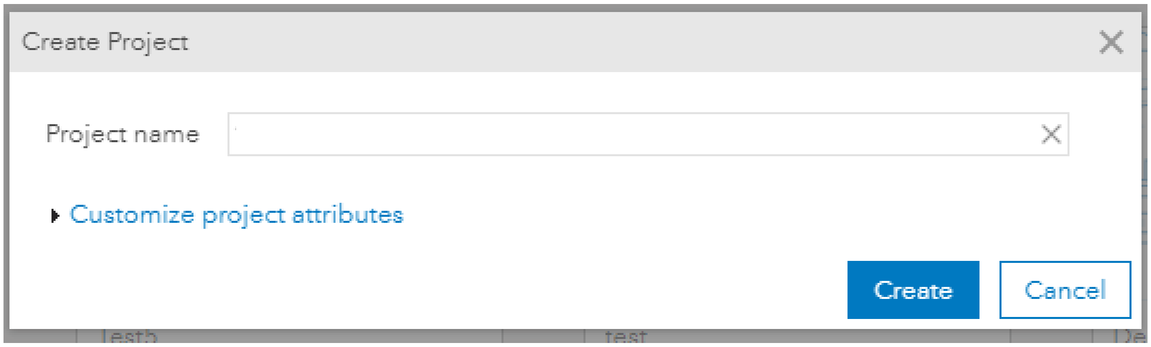

4. You will see the following image as the new project is created.

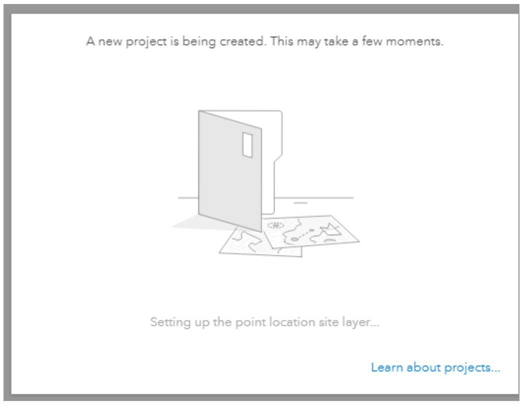

Make sure that the **Open new projects as soon as they are created** box is **<u>un</u>**checked and then select **OK**.

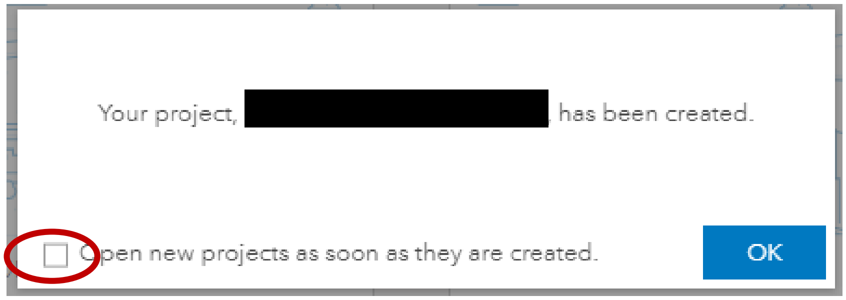

Once the project is named and created it will appear on your **Projects** page.

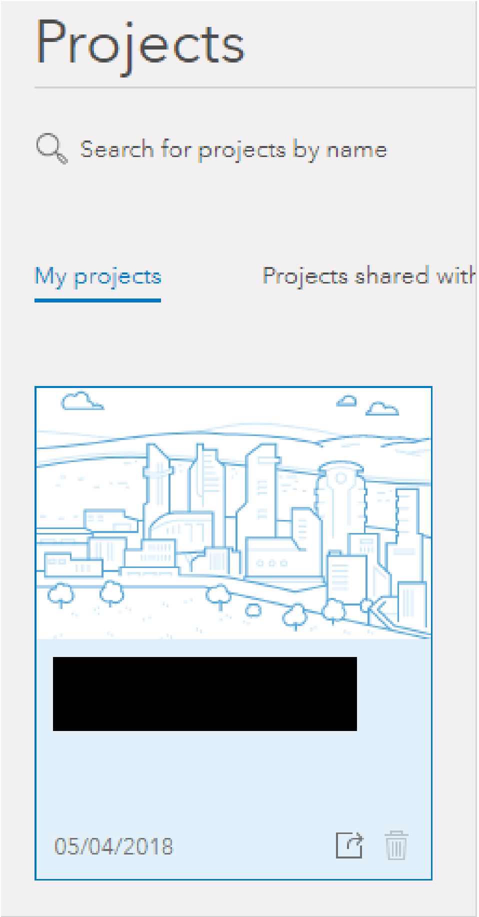

5. Use your cursor to hover over the thumbnail of your project. The thumbnail will become highlighted in grey. Click **Open**.

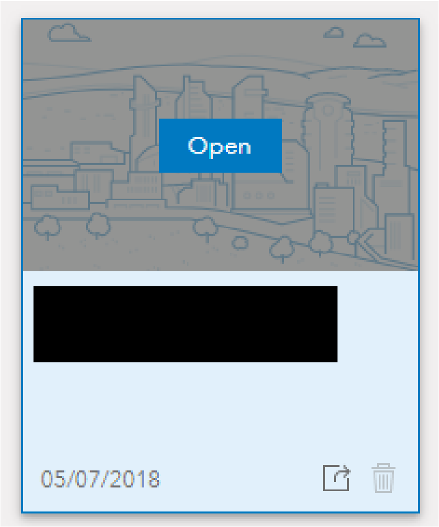

6. When the project opens your workspace will look similar to this:

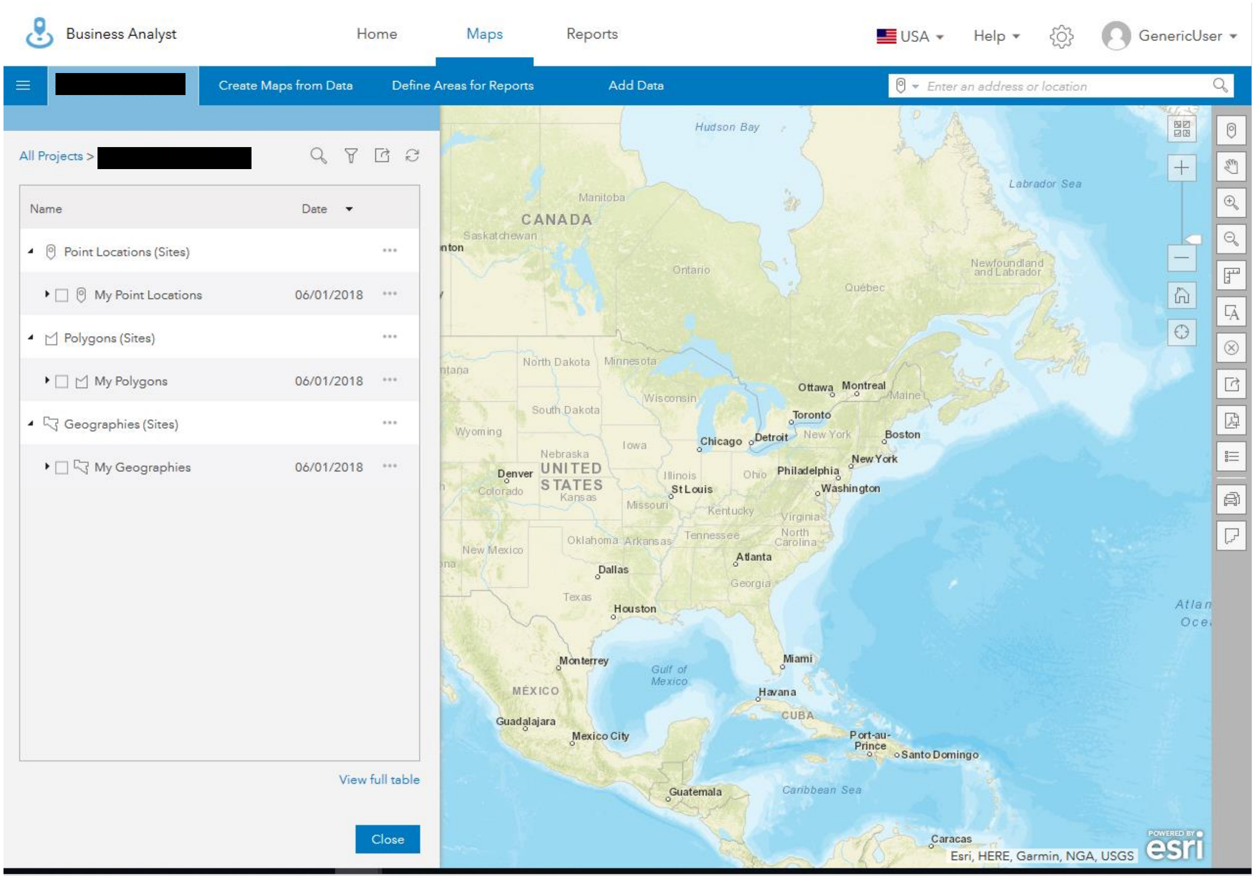

7. In the blue ribbon at the top of the screen click on the **Define Areas for Reports** tab and click on **Select Geography**.

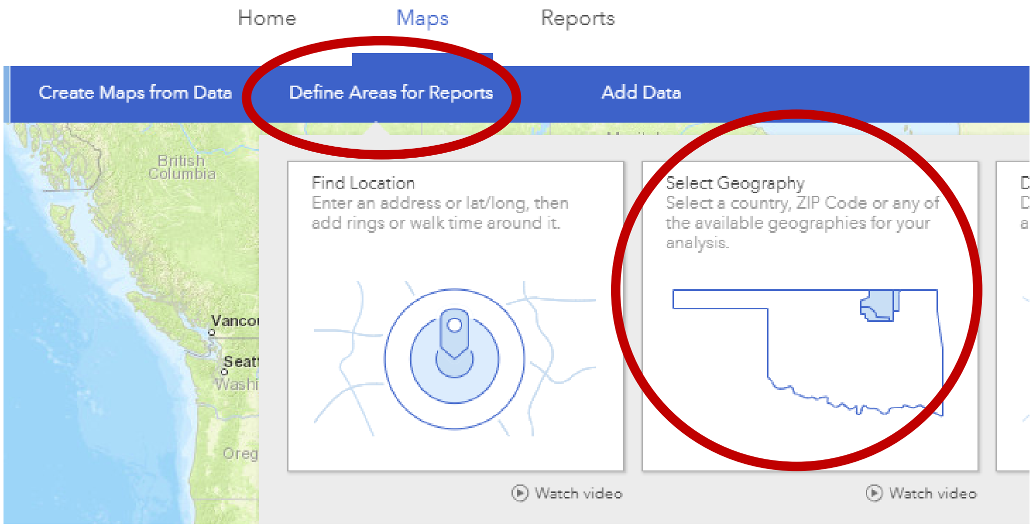

8. The Select Geography Box will open. This is where you will define the area and the geographic level for which you want to abstract data.

   Click **Select from Full list**.

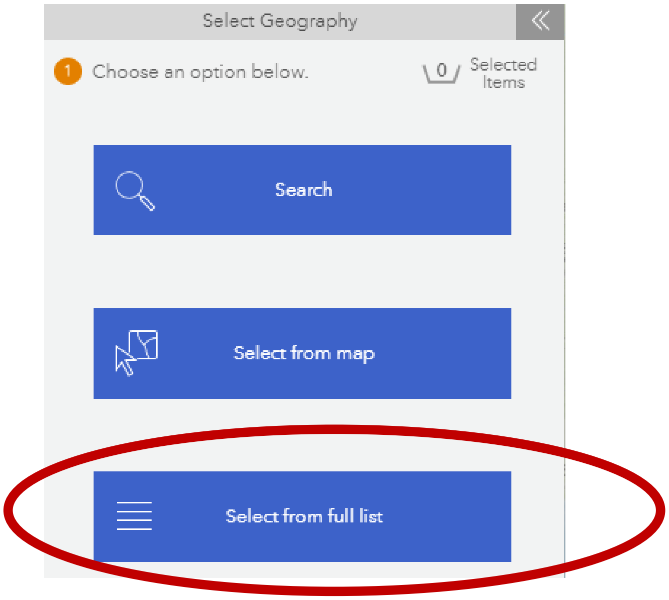

9. You will then have the option of selecting **Census Tracts** as the level of Geography.

   Click on **Census Tracts.**

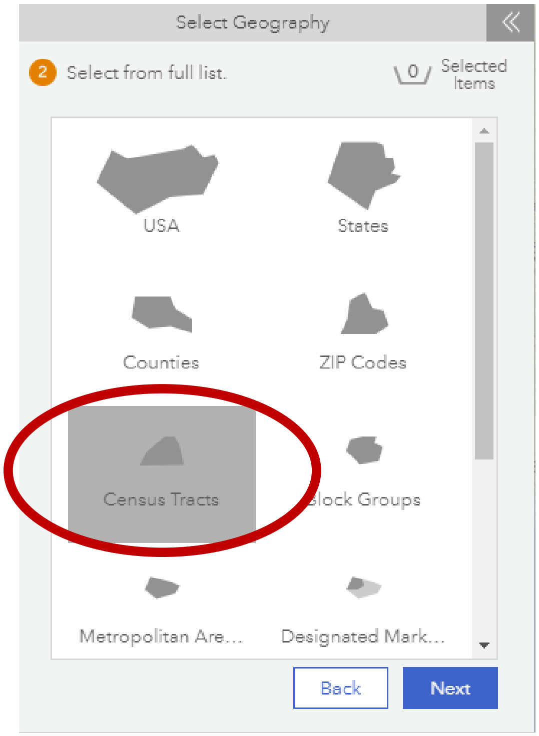

10. The Select Geography box will open on the left of your screen and should look like the following image:

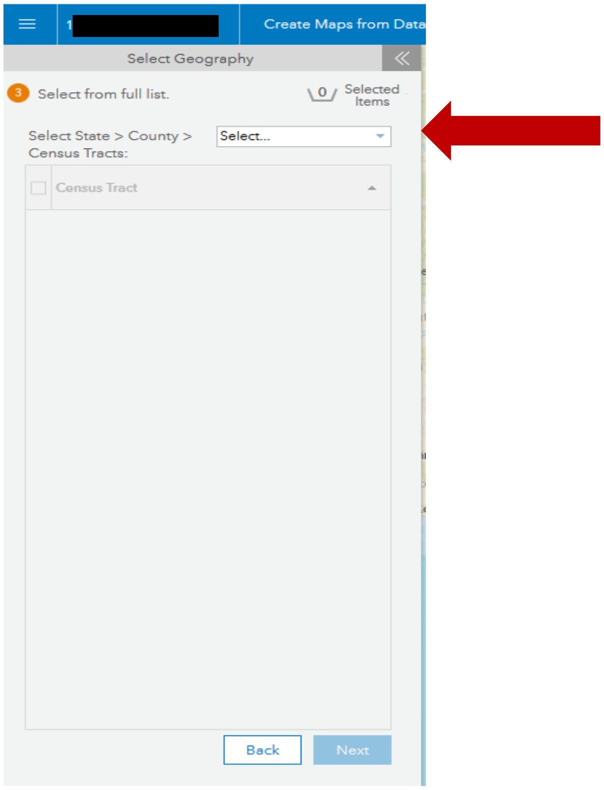

- For **Select State>County>Census Tracts**, select your state from the drop down menu.

  Note: This protocol uses example images from Alabama.

- Another drop down menu will appear underneath.

  Identify the first county on the list of counties that you created from ArcMap (see **Step 4** of **Part 4** of this protocol for more information on how to create the list of counties that contain geocoded address locations.)

  For this example, we are selecting **Jefferson County** from the dropbdox menu.

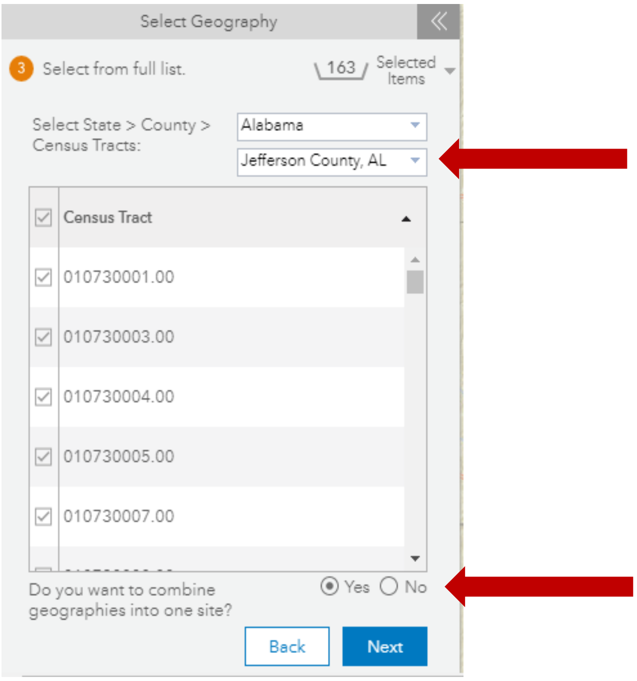

- Now that you have selected the State and the County that contain the census tracts that you want to abstract data for, the Select Geography box will contain a list of all Census Tracts in that county.

  Check the small box next to the header Census tractto select all tracts in Jefferson County.

  Note: In this example, there are 163 Census Tracts within Jefferson County, hence, the number of selected items is 163.

  Click **Next.**

11. The geographic area that you have selected (e.g., Jefferson County, Alabama) has been saved and you will see the following image:

    Click **Done.**

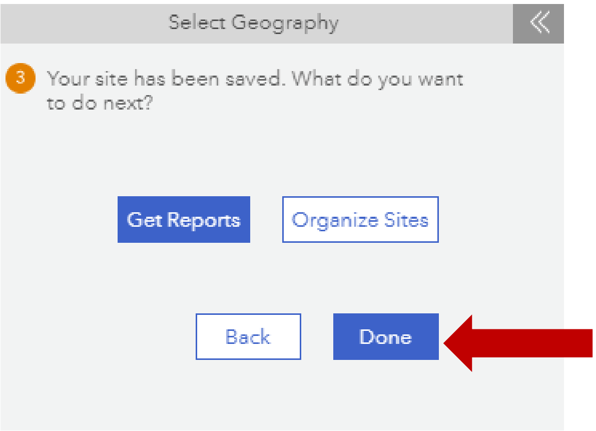

Your screen should now resemble the following image:

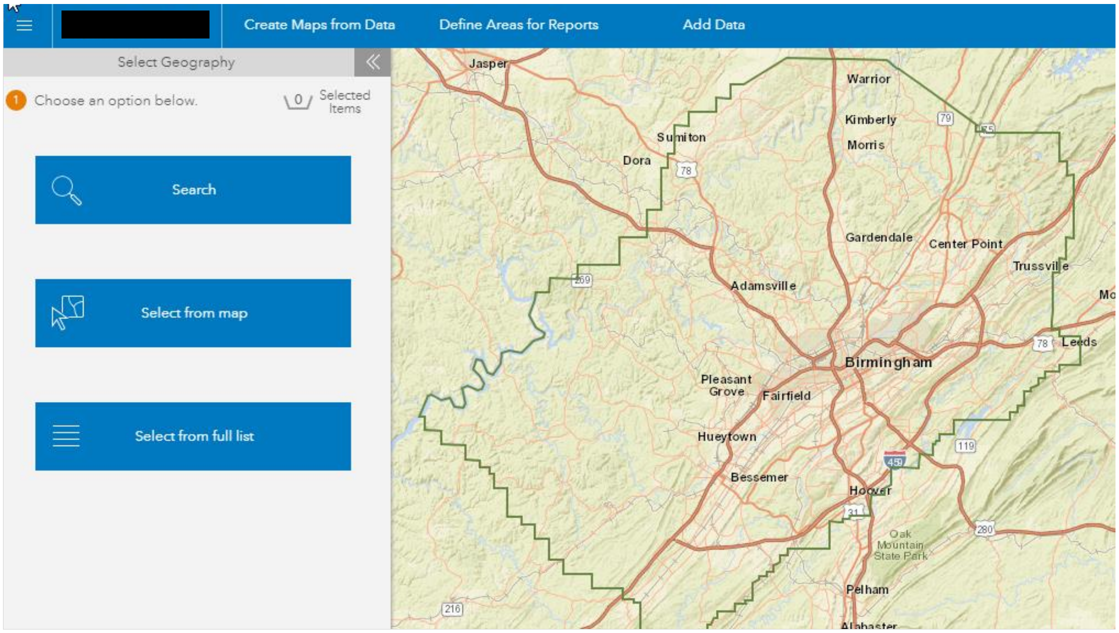

12. The next step is to designate variables for which you want to abstract data within the selected county.

    Click on the **Create Maps from Data** tab in the blue banner.

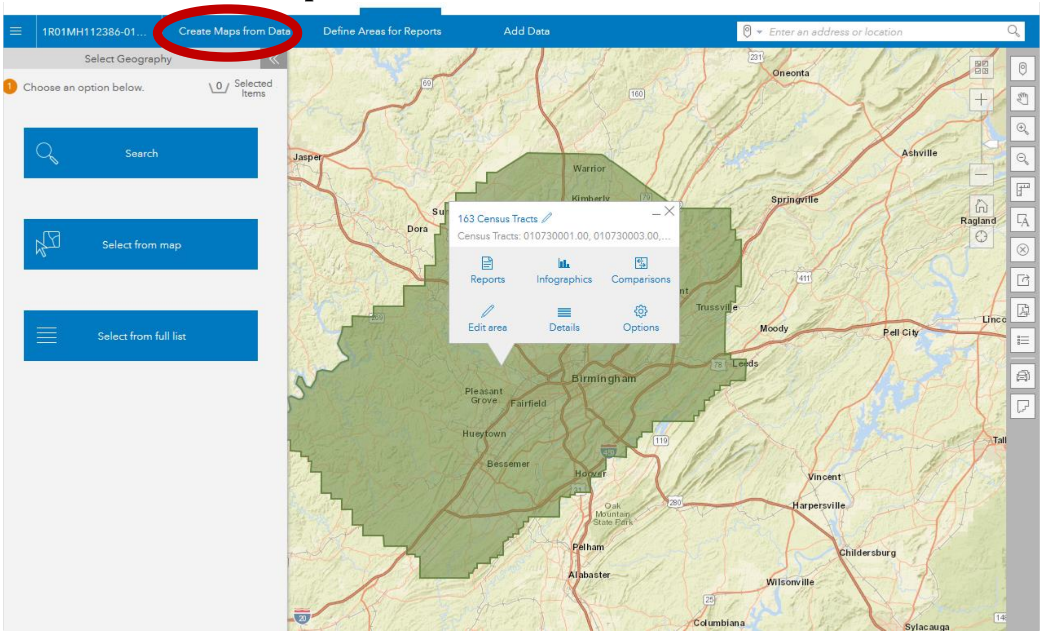

    A panel of options will open. Select **Smart Map Search.**

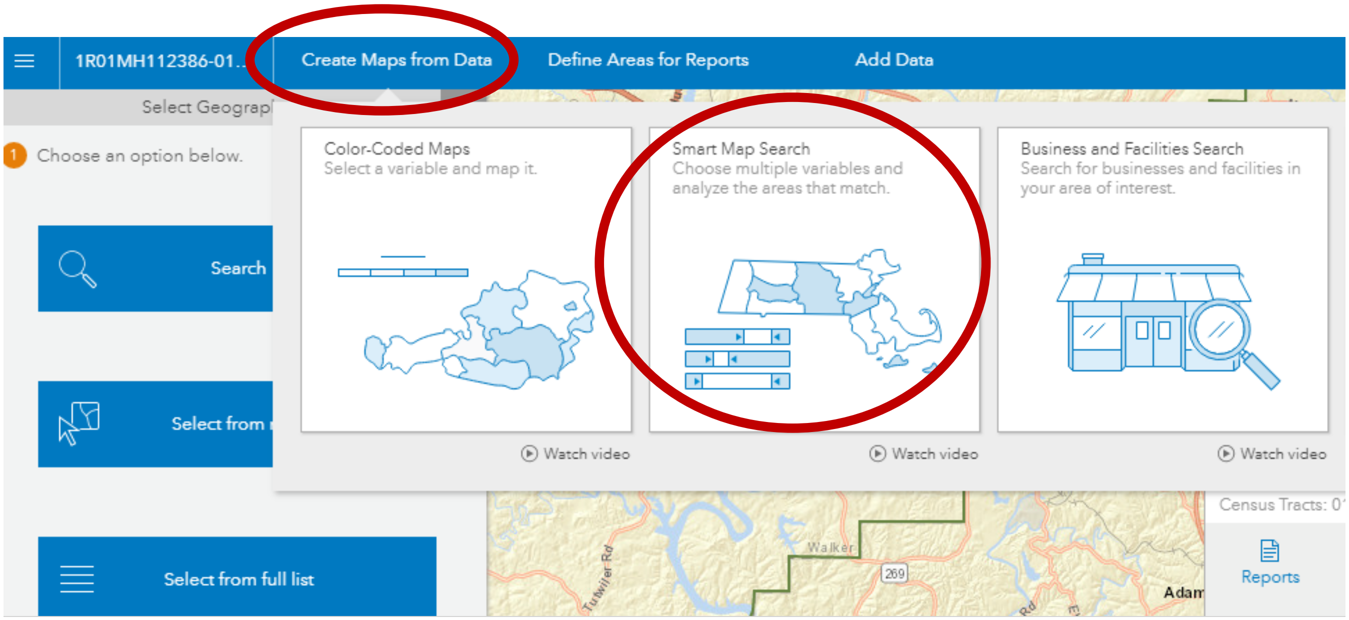

13. In the **Smart Map Search** box, click on the icon of the two buildings to open a menu of data categories.

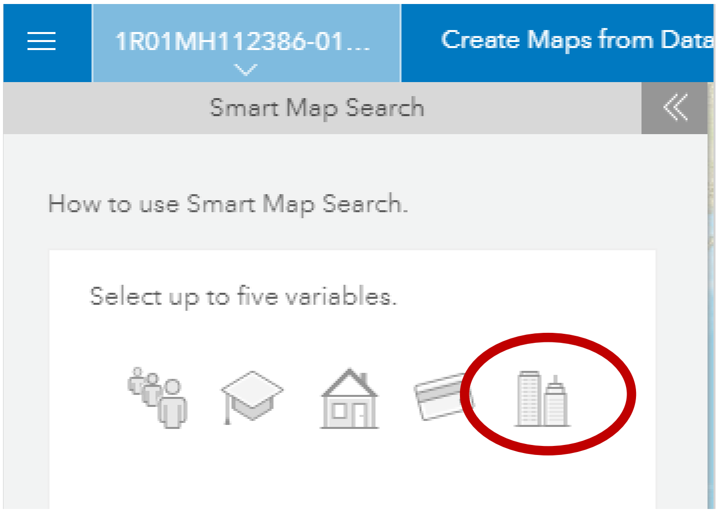

You will now see 6 categories of variables. Crime data is not in one of these categories so you will need to click **Browse all variables**.

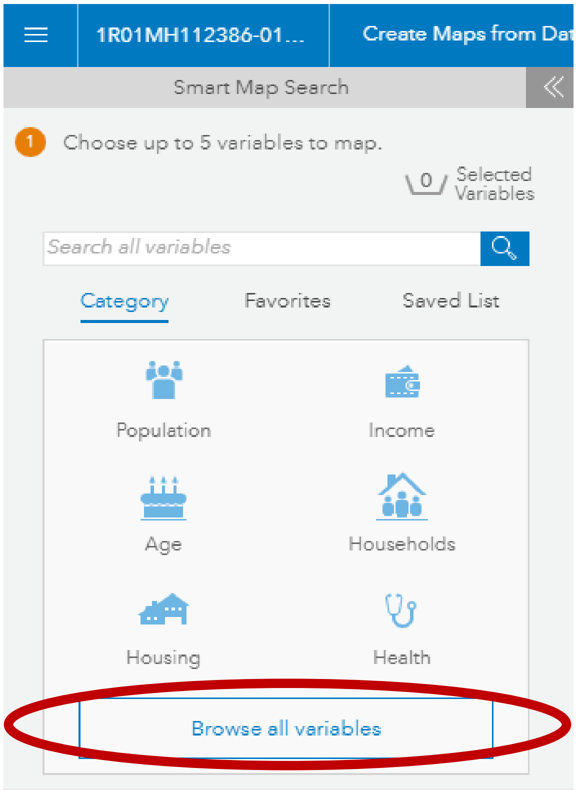

14. After clicking **Browse all Variables,** you will see the following box:

    Use the arrow on the right side of the screen to scroll to a second page that contains additional variables.

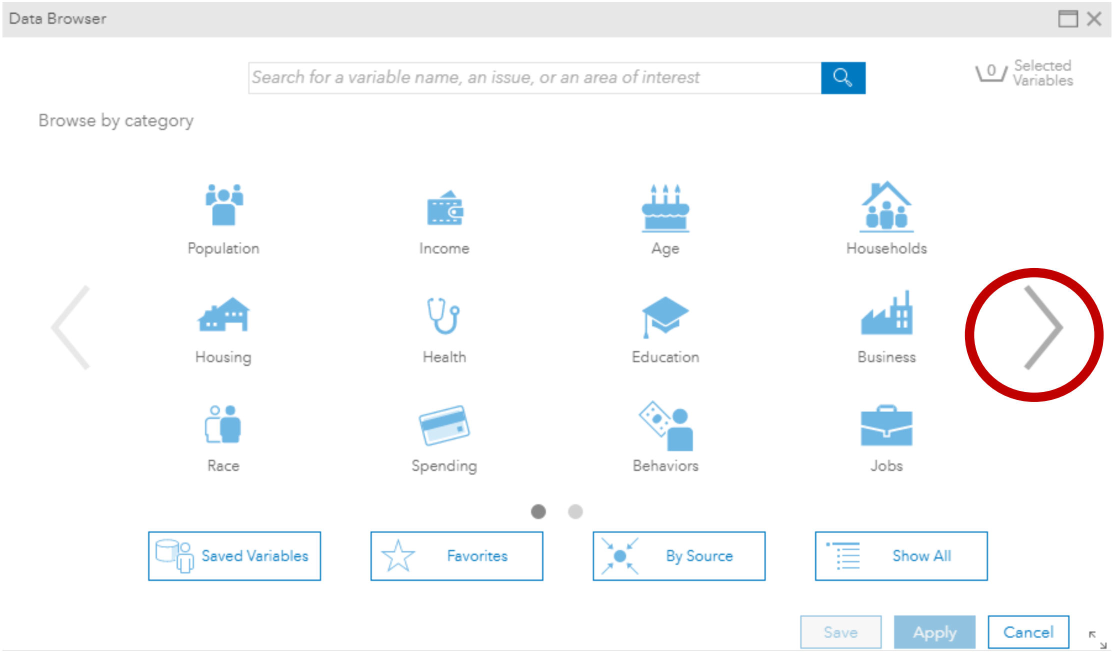

- Once you have scrolled to the next page click on the **Crime icon** (a thumbnail of a set of handcuffs)**.**

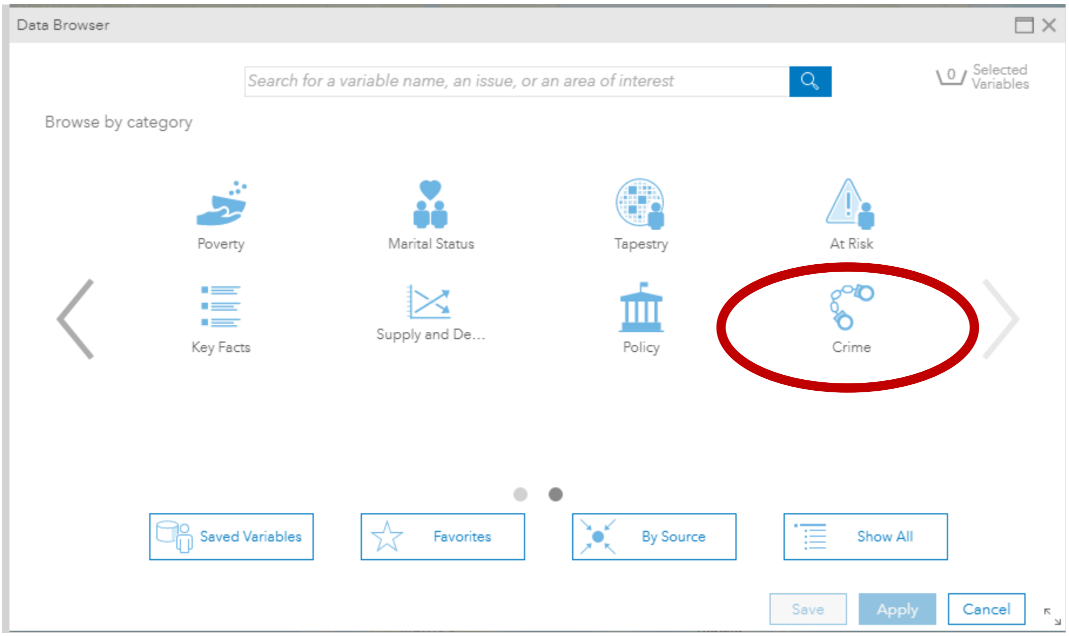

The **Data Browser** box will open.

15. Check the box for **2018 Murder Index** and **2018 Assault Index** (or other indices of interest) and then click **Apply**. **Note**. The corresponding years for Murder Index and Assault Index are subject to change depending on study Aim. For information about which year to select, please contact the MPIs.

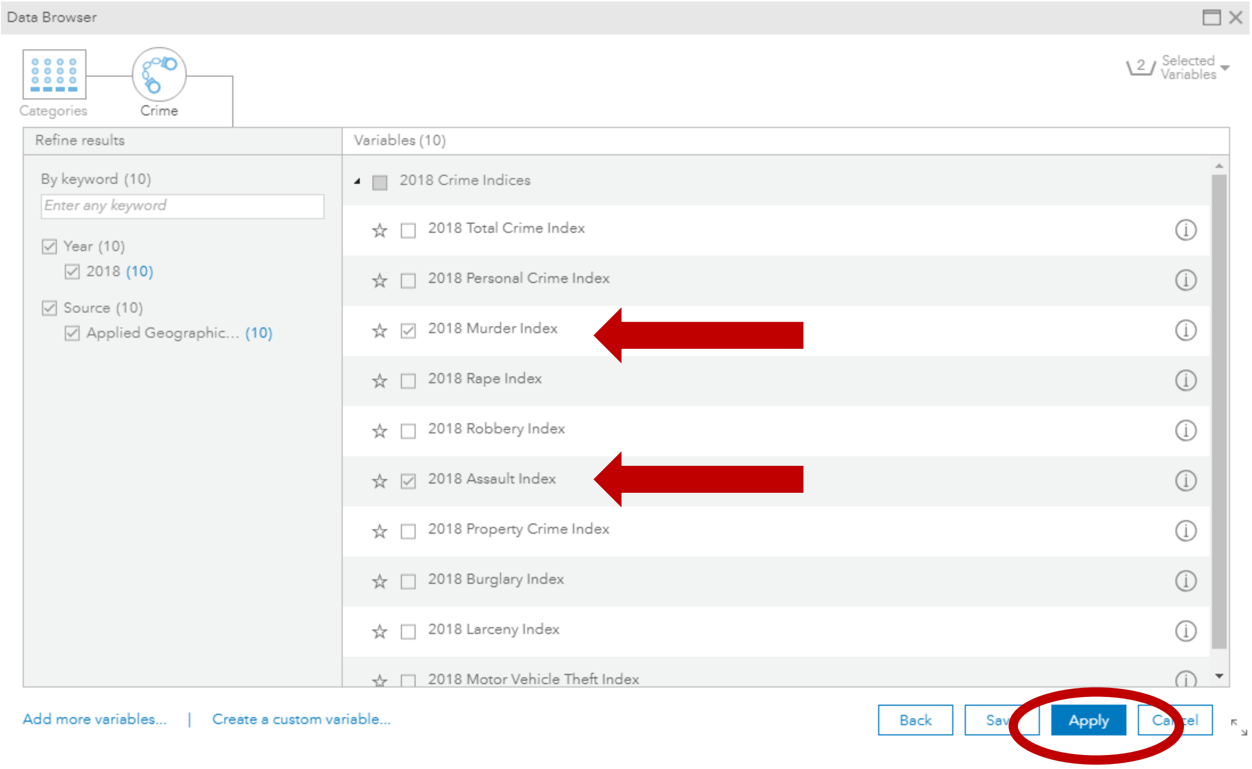

16. The **Smart Map Search** box will reopen once the 2018 Murder Index and 2018 Assault Index are retrieved. The map (depending on the location for which you are abstracting data) on your screen will resemble the following screenshot:

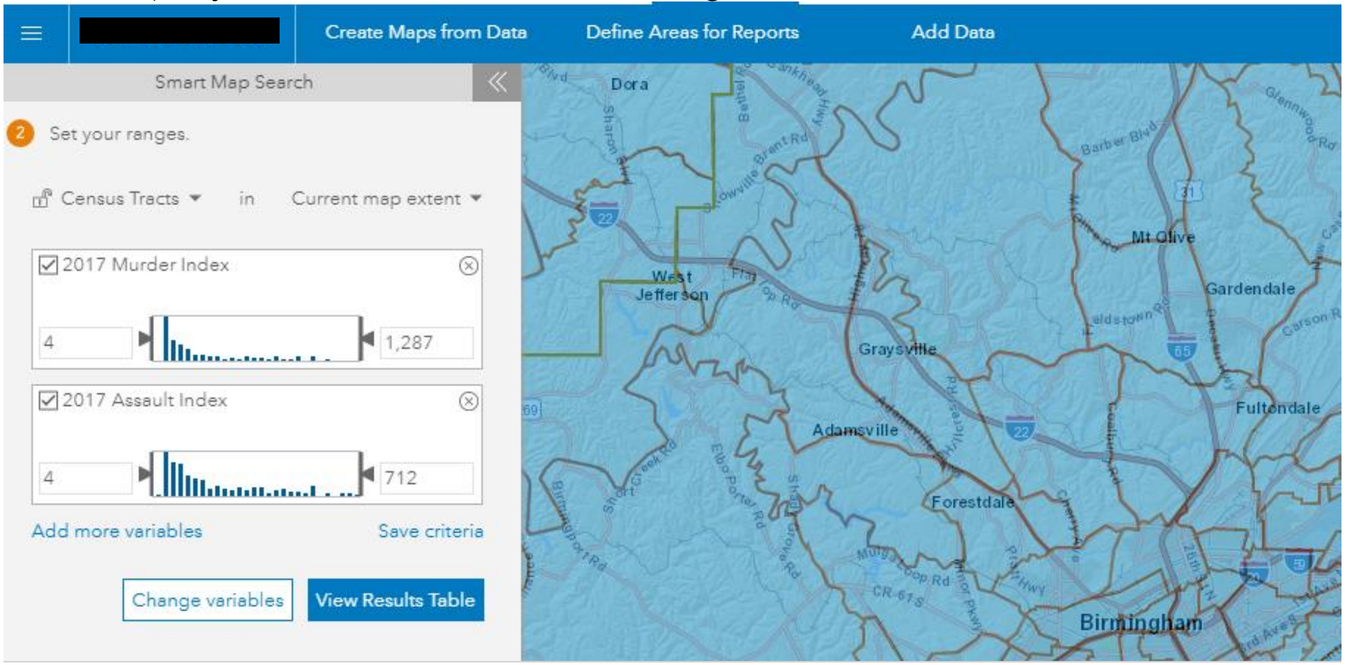

17. In the Smart Map Search Box click on the drop down menu titled **Current map extent**.

Select the number of census tracts in the county. In this example, there are 163 Census Tracts in Jefferson County, Alabama so we will select **163 Census Tracts** from the dropdown menu. (BAO uses the number of Census Tracts in each County as its default naming convention.)

Click **View Results Table.**

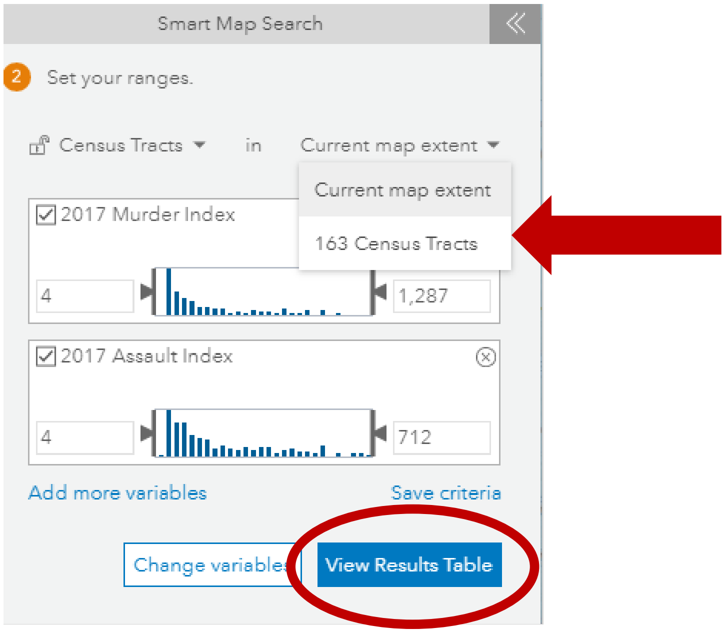

18. A list of census tracts and the corresponding 2018 Murder Rate Index and 2018 Assault Rate Index for each census tract will come up for the specified county.

    Click **Export to Excel**. This will export the Murder Index and Assault Index for each of the 163 Census Tracts for Jefferson County.

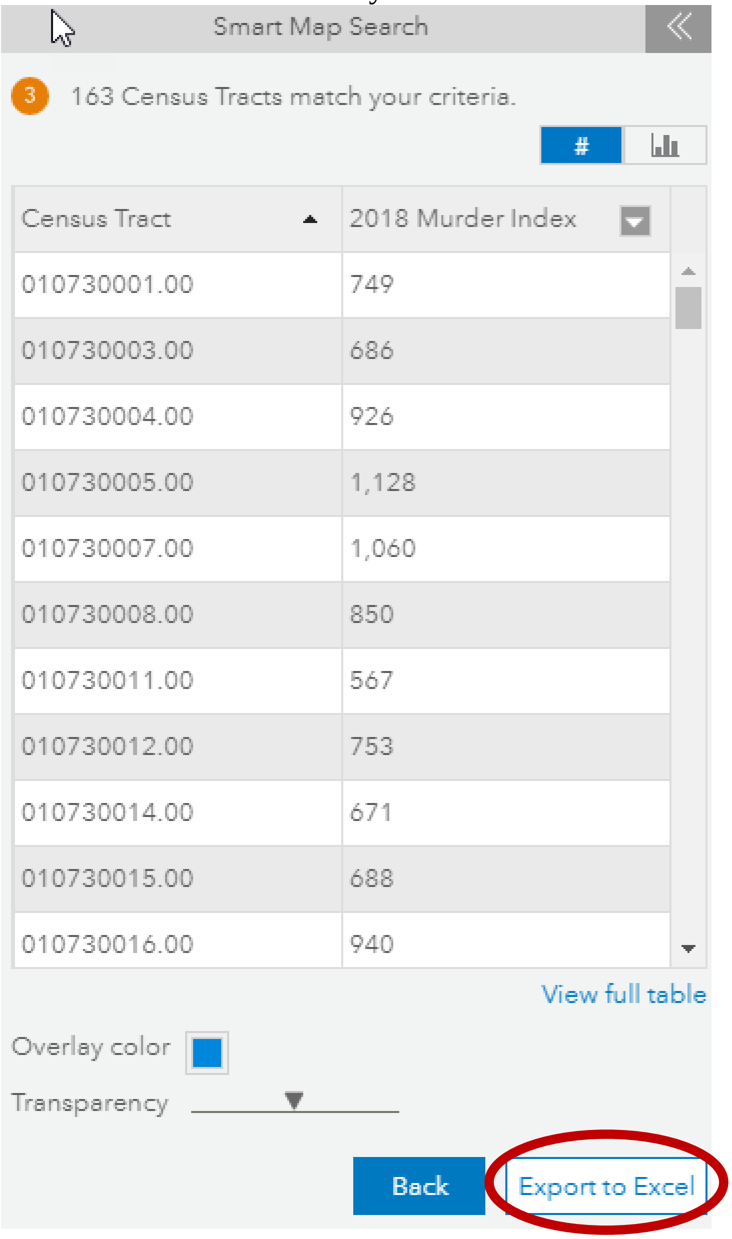

19. An Excel file with the name **Smart Map Search** will automatically download.

    Open the **Smart Map Search** Excel file.

20. The Smart Map Search Excel file will contain three columns that contain the relevant data for Jefferson County:

    1. Census Tract Number (Column A)
    2. 2018 Murder Index (Column B)
    3. 2018 Assault Index (Column C)

    Save the Smart Map Search Excel file for the county-specific crime data (e.g., as **Smart Map Search_jeffersoncnty)** to your clinic desktop computer.

21. You will now need to abstract crime data for the rest of the counties that are included in the **SelectedTracts5.10.18rf** Excel file (as described in **Part 3b** of this protocol).

    To absract crime data for the remaining counties, follow the same process that you used to abstract crime data for Jefferson County as outlined in the previous Steps 6 – 22 of this section.

22. Each county for which you abstract crime index data will download as an Excel file with the name **Smart Map Search**. To keep track of the counties for which you abstract crime data, rename the downloaded Excel file with the name of the corresponding county (e.g., **Smart Map Search_jeffersoncnty** or **Smart Map Search_autaugacnty**).

    Save each Smart Map Search Excel file that contains crime data to your clinic desktop computer. Also, create an **Archive** subfolder for crime data.

    Double check that crime data have been downloaded from BAO for all counties that are included in your **SelectedTracts5.10.18rf** Excel file and that contain at least one geocoded residential address.

Step 3: Creating a single Excel file of crime data for all counties in your state

The crime data for all counties that you pulled will need to be merged into a single Excel file. This crime Excel fill will be merged with census tract data as described in **Part 6a** of this protocol.

23. Create a new Excel file. Name the file so that it is specific to your state (e.g., **CrimeData_ALcounties_all).** Save the new Excel file to your clinic desktop computer. This file will contain the merged crime data from all counties in your state.

24. Navigate to the folder on your desktop and select and open any Smart Map Search Excel file (e.g., **Smart Map Search_jeffersoncnty** or **Smart Map Search_autaugacnty**).

    Copy the three columns of data from the Smart Map Search Excel file and **paste** the columns into the Excel file that will contain crime data for all counties in your state (e.g., into the **CrimeData_ALcounties_all** file).

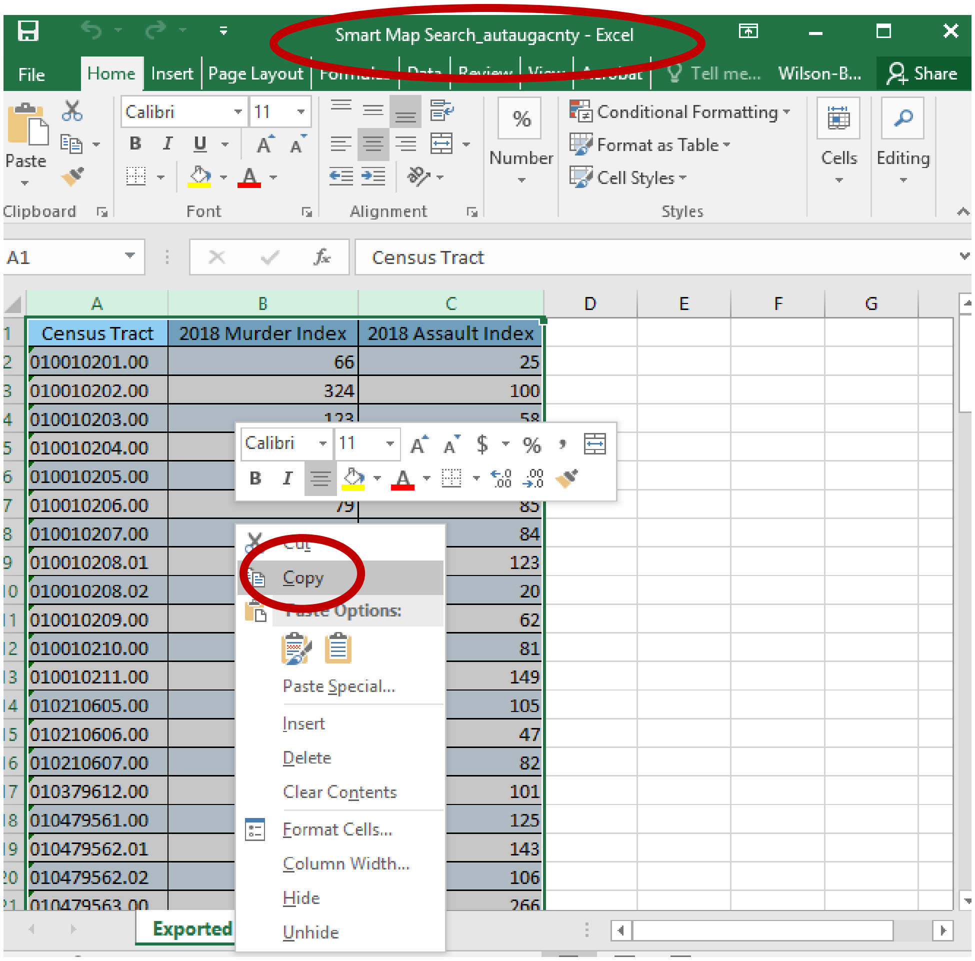

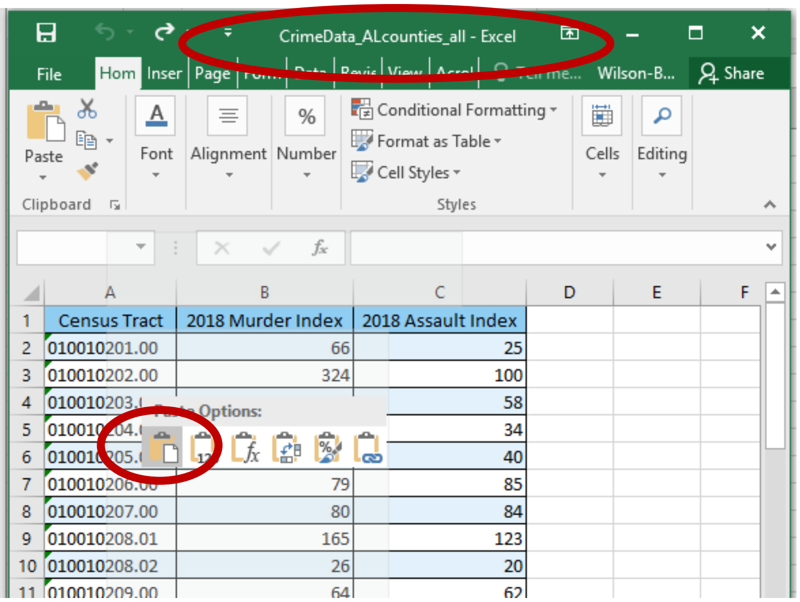

25. Open **all** of the county-specific Smart Map Search Excel files in the relevant folder on your clinic desktop computer. Starting with the first Census tract in the Excel file (i.e., do not copy the headings for the remaining counties), copy the three columns of data and add data into the Excel file that contains crime data for all counties in your state. After copying and pasting the data from a county file, please move that specific county file to your **Archive** folder.

To add data for additional counties, right click on the first empty cell in column A and select **Paste**.

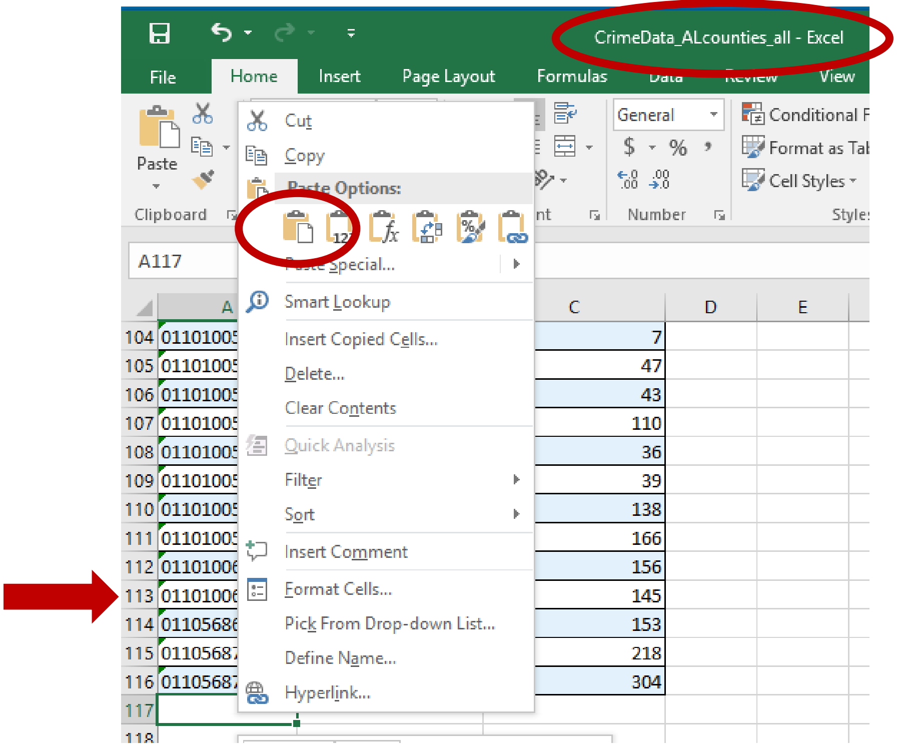

26. Once you have added the crime data for all counties in your state to a single Excel file, (e.g., CrimeData_ALcounties_all), save the file to your clinic desktop computer.

Step 4. Reformatting the Esri Census Tract Numbers

The census tract number used by Esri is different from that used by the Census Bureau and will need to be reformatted to match the census tract identifier used by the Census Bureau.

27. Open the Excel file (e.g., CrimeData_ALcounties_all) that contains crime data for all counties in your state.

    To reformat the Esri BAO Census tract numbers:

- Select all the Census tract numbers in column A by left clicking on cell A2.
- Hold and scroll down to the end of the data in Column A.
- Now that you have selected all cells in Column A that contain data scroll back to the top of the column.

Note: If you hover over the warning symbol, you will see the warning that **The number in this cell is formatted as text or preceded by an apostrophe.**

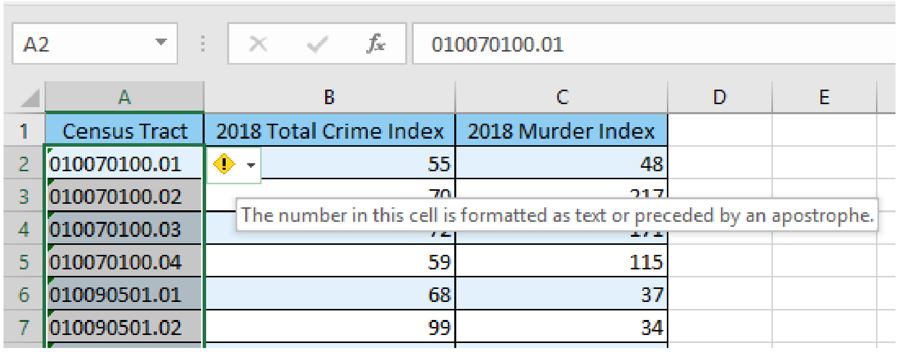

- Left click on the warning symbol to open the dropdown menu and select **Convert To Number.**

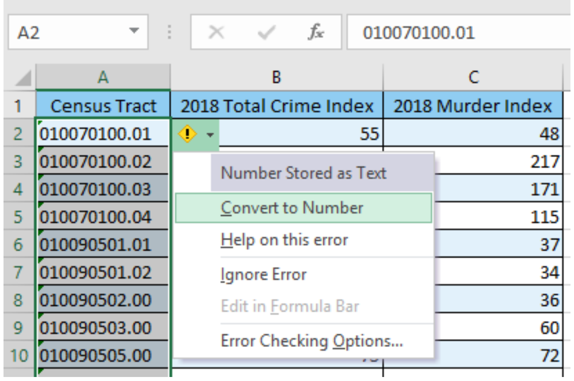

The numbers will be reformatted; however, some numbers will still have a decimal point and two decimal places that will need to be removed.

- Insert a new column in column B.
- In this new blank column select cell B2

	A	B	C	D
1	Census Tract		2018 Murder Index	2018 Assault Index
2	010730001.00		745	533
3	010730003.00		785	427
4	010730004.00		948	523
5	010730005.00		1,154	612
6	010730007.00		1,054	573
7	010730008.00		867	426
8	010730011.00		586	329
9	010730012.00		768	406

- In the function box type =A2*100

	A	B	C	D
1	Census Tract		2018 Murder Index	2018 Assault Index
2	010730001.00	=A2*100	745	533
3	010730003.00		785	427
4	010730004.00		948	523
5	010730005.00		1,154	612
6	010730007.00		1,054	573
7	010730008.00		867	426
8	010730011.00		586	329
9	010730012.00		768	406

- Hit enter then hover over the left bottom corner of the cell and double click to copy the formula to the remainder of the cells in column B.

	A	B	C	D
1	Census Tract		2018 Murder Index	2018 Assault Index
2	010730001.00	1073000100	745	533
3	010730003.00	1073000300	785	427
4	010730004.00	1073000400	948	523
5	010730005.00	1073000500	1,154	612
6	010730007.00	1073000700	1,054	573
7	010730008.00	1073000800	867	426
8	010730011.00	1073001100	586	329
9	010730012.00	1073001200	768	406

- Copy all of Column B and paste the values into Column A using **Paste Values**

Note: It is imperative that you use Paste Values or you will simply copy and paste the formulas into Column A that will result in a broken formula reference.

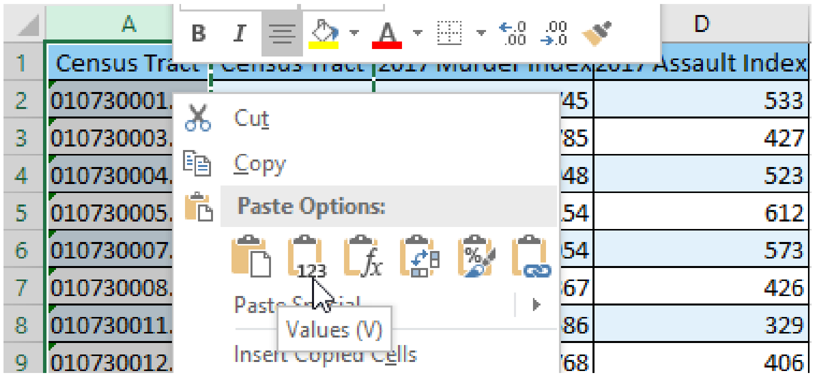

- Delete Column B. Your worksheet should now have three Columns resembling the following screenshot.

	A	B	C
1	Census Tract	2018 Murder Index	2018 Assault Index
2	010730001.00	745	533
3	010730003.00	785	427
4	010730004.00	948	523
5	010730005.00	1,154	612
6	010730007.00	1,054	573
7	010730008.00	867	426
8	010730011.00	586	329
9	010730012.00	768	406
10	010730014.00	708	358

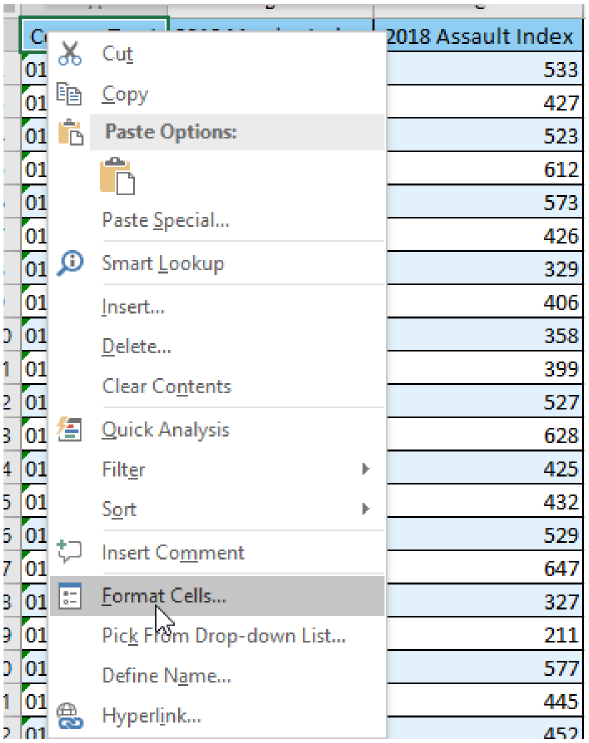

28. Rename the edited Excel file that contains crime data for all counties in your state (e.g., **CrimeData_ALcounties_all**) using the naming convention described below.

**Data File Naming Convention for Crime Data Abstraction**	
**Component**	**Description**
**Example file name**	**R01.Crime.AL.Date**
**R01**	Project Abbreviation
**Crime**	For the type of data
**AL**	State abbreviation
**Date**	The date the data were abstracted and downloaded.

- Save the file to your clinic desktop computer.

  Logout of Business Analyst.

This was the last activity needed to abstract crime data for census tracts within each county in your state. These abstracted data will be merged with the census tract data during the reformatting process in Part 6a.

Part 5a: Obtaining Neighborhood Socioeconomic Data for Census Tracts

Data Source: Latest Available American Community Survey (ACS) Five-Year Estimates from the United States Census Bureau.

Objective: To abstract data at the Census tract level for the three socioeconomic variables.

Source(s):	Example Socioeconomic Variables
U.S. Census Bureau 5-year estimates from the American Community Survey	% below the poverty line<br>% unemployed<br>% less than high school education

Completing this activity will yield neighborhood-level socioeconomic data for the participants' census tracts of residence.

Step 1. Accessing the data.census.gov Website

1. Navigate to the data.census.gov webpage using the following link: https://data.census.gov/cedsci/

Click on **Advanced Search**.

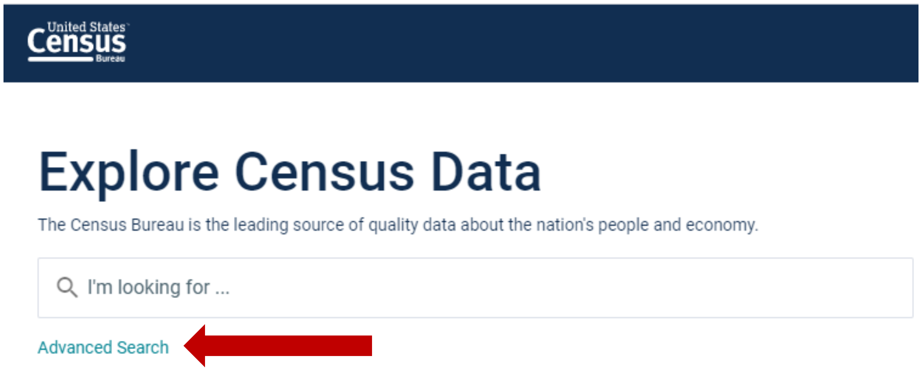

Step 2. Obtaining Data for % Below the Poverty Line and % Unemployment

2. On the Advanced Search page, enter the ID for the Table that includes poverty and unemployment data.

    - Enter the Table ID: **DP03**
    - Designate the area(s) for which you want to download the relevant poverty and unemployment data. To do this, click on **Geography → Tract → Alabama** (or **North Carolina**) → and check **All Census Tracts within Alabama** (or **All Census Tracts within North Carolina**).
    - Click **Search.**

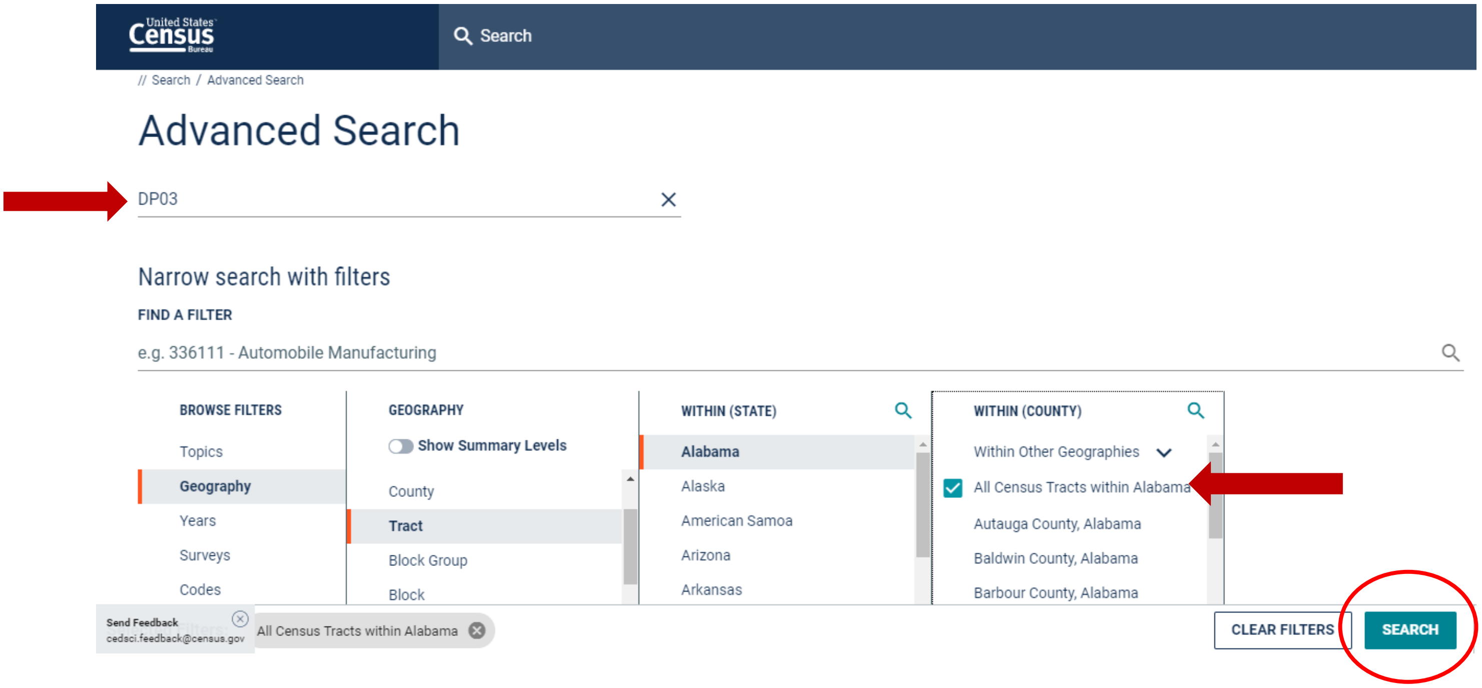

3. Under Tables, you should see **Selected Economic Characteristics.** Click on **View All Tables.**

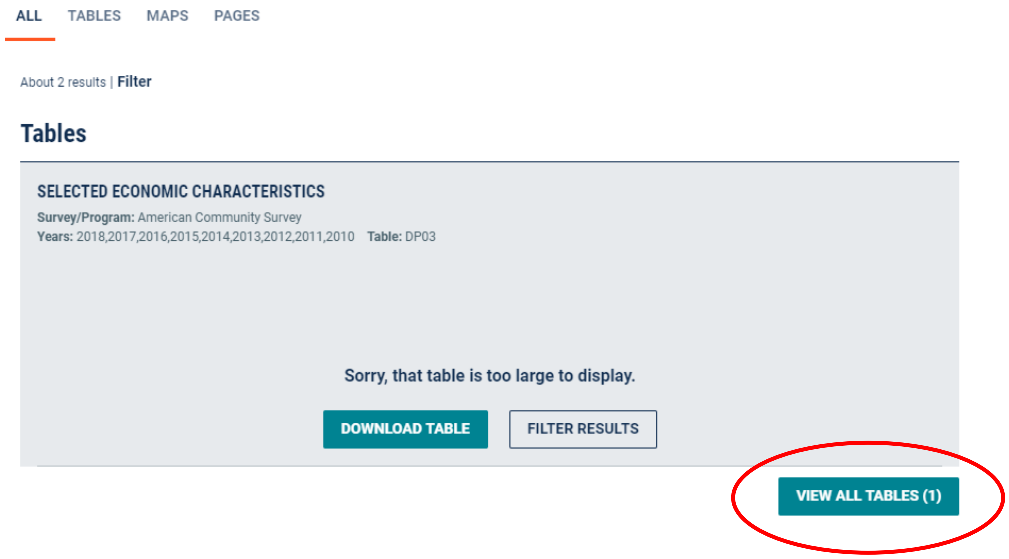

4. After selecting View All Tables, at the top of your screen, confirm that the selected table has the following characteristics:

Table Name:	**Selected Economics Characteristics**
Survey/Program:	**American Community Survey**
TableID:	**DP03**
Product:	**2018: ACS 5-Year Estimates Data Profiles.**

Click **Customize Table**.

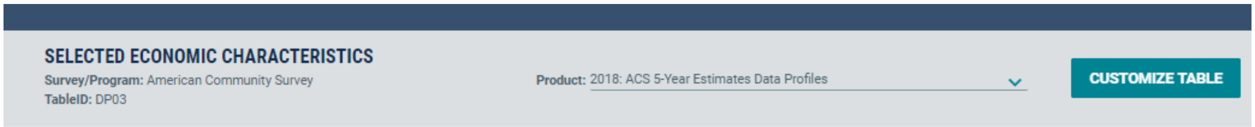

Note: It is important to ensure that you are using the most recent 5-year ACS estimates available, which will be 2018 or later.

Under **Geography**, confirm that all census tracts in your state are selected.

Click **Close**. Click **Download Table**.

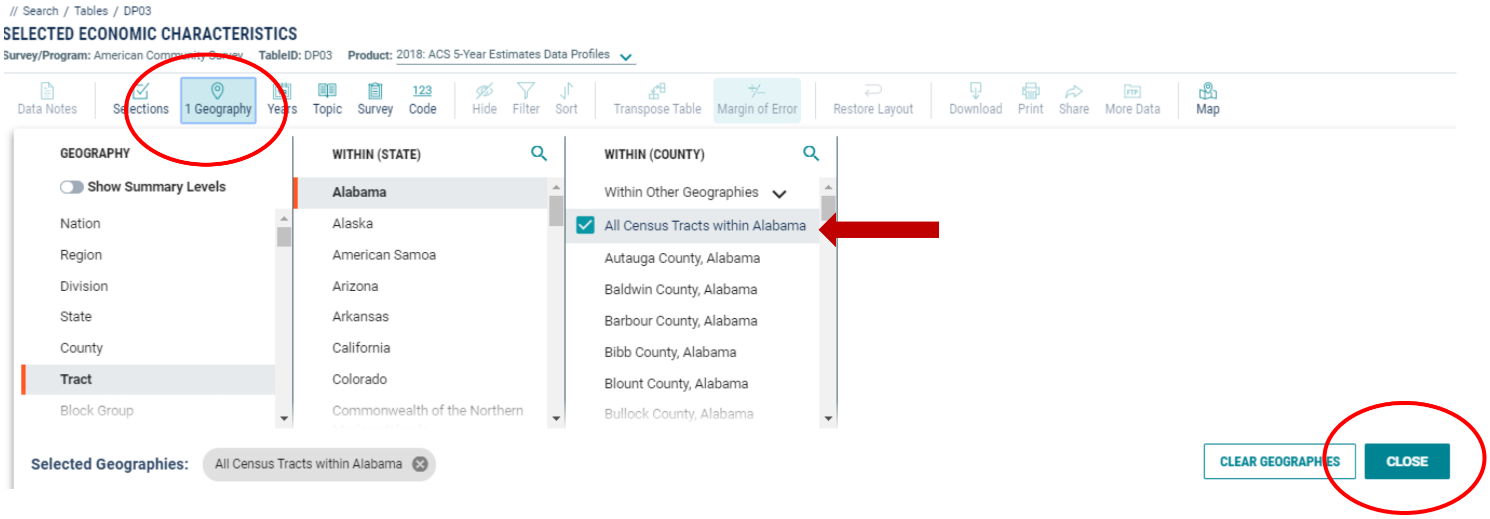

5. Make sure that the most recent year is selected for the 5-Year estimates. Download the DP03 table as a CSV file by clicking **Download**.

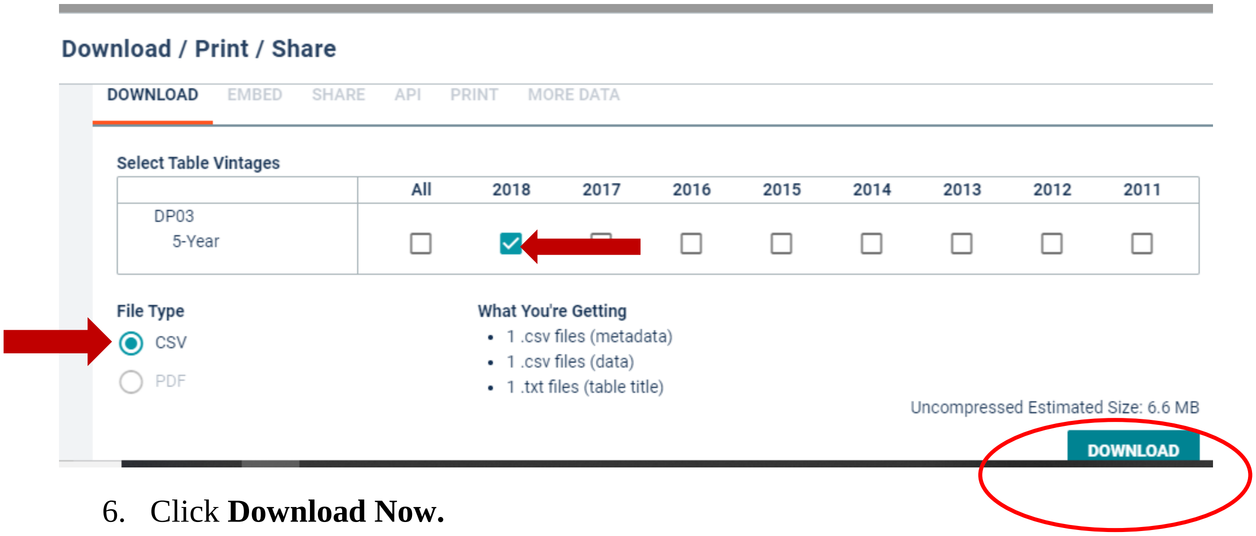

6. Click **Download Now.**

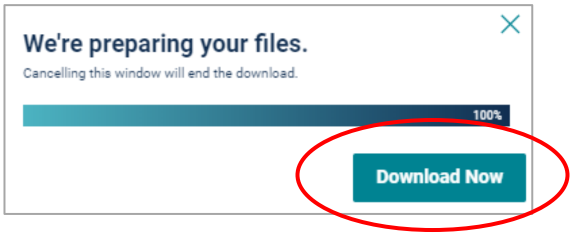

7. The downloaded file containing the data for the % poverty and % unemployment data will have the title ACSDP5Y2018.DP03_ and contain the following three files:

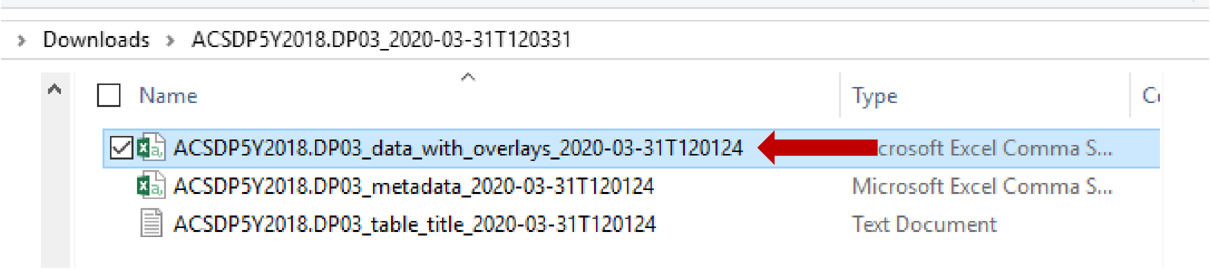

NOTE: The folder and file names will be slightly different depending on the date the download was performed and the state for which the data were abstracted.

8. Save the **downloaded** file on your clinic desktop computer. Also, create an **Archive** subfolder for socioeconomic data.

9. Unzip the file and open the **ACSDP5Y2018.DP03_data_with_overlays** file in Excel.

Rename the file so that it is specific to the socioeconomic indicator(s) and year of the ACS 5-year estimates (e.g., **PvrtyEmploy_ACS_18).** Save the renamed file on your clinic desktop computer.

Step 3. Obtaining Data for % Less than a High School Education

10. Return to the Explore Census Data homepage by clicking on the United States Census Bureau icon in the top left hand corner of your screen.

    Click **Advanced Search**.

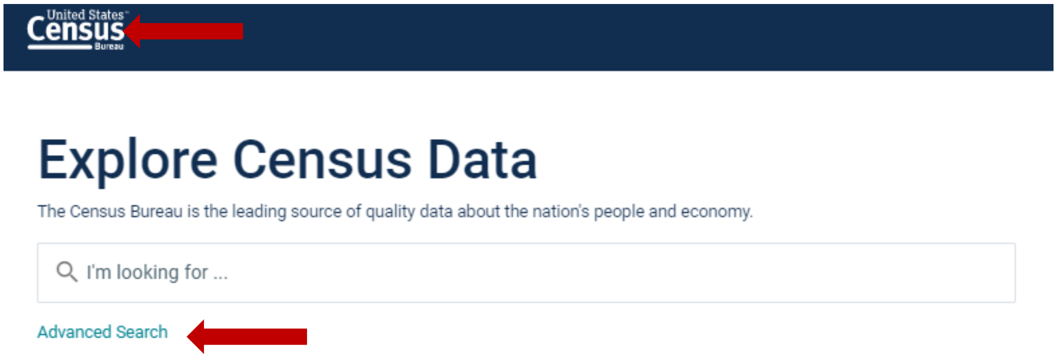

11. On the Advanced Search page, first clear all previous filters by clicking **CLEAR FILTERS**.

Then, enter the ID for the Table that includes high school education data.

- Enter the Table ID: **S1501**
- Designate the area(s) for which you want to download the relevant poverty and unemployment date. To do this, click on **Geography** → **Tract** → **Alabama** (or **North Carolina**) → and check **All Census Tracts within Alabama** (or **All Census Tracts within North Carolina**).
- Click **Search.**

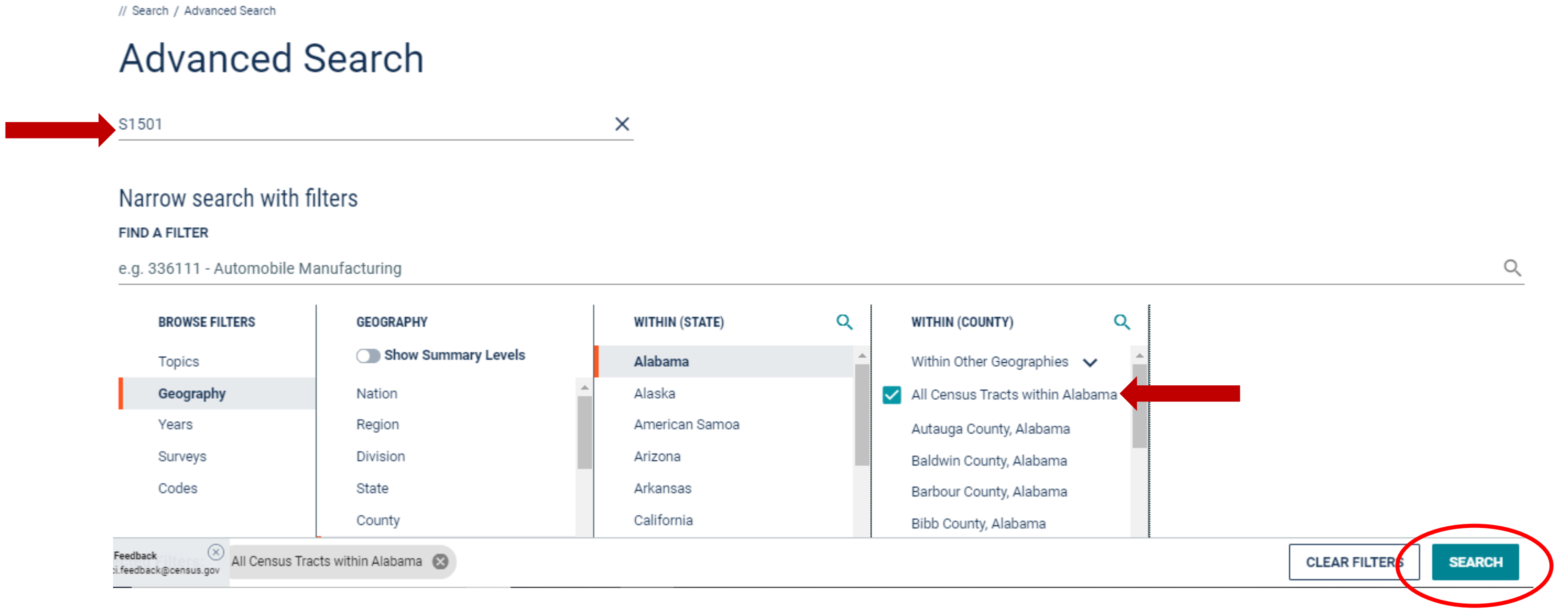

12. Under Tables, you should see **Educational Attainment.** Click on **View All Tables.**

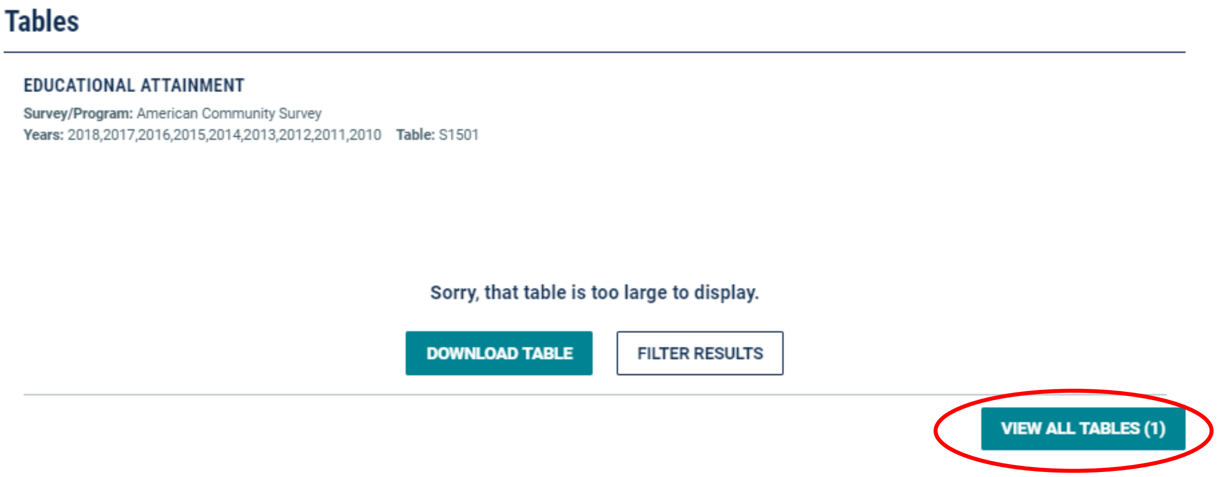

13. After selecting View All Tables, in the top of your screen, confirm that the selected table has the following characteristics:

Table Name:	**Educational Attainment**
Survey/Program:	**American Community Survey**
TableID:	**S1501**
Product:	**2018: ACS 5-Year Estimates Subjects Tables**

Click on **Customize Table** in the top right hand corner of your screen.

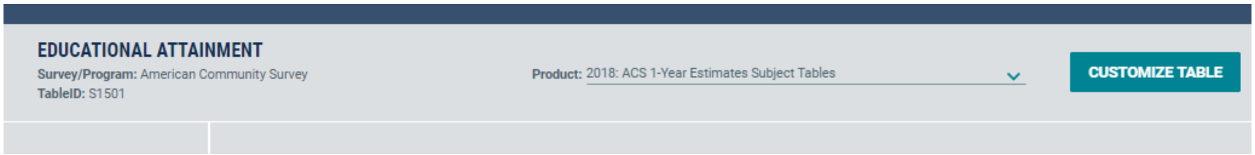

Note: It is important to ensure that you are using the most recent 5-year ACS estimates available, which will be 2018 or later.

Under **Geography**, confirm that all census tracts in your state are selected.

Click **Close**. Click **Download Table**.

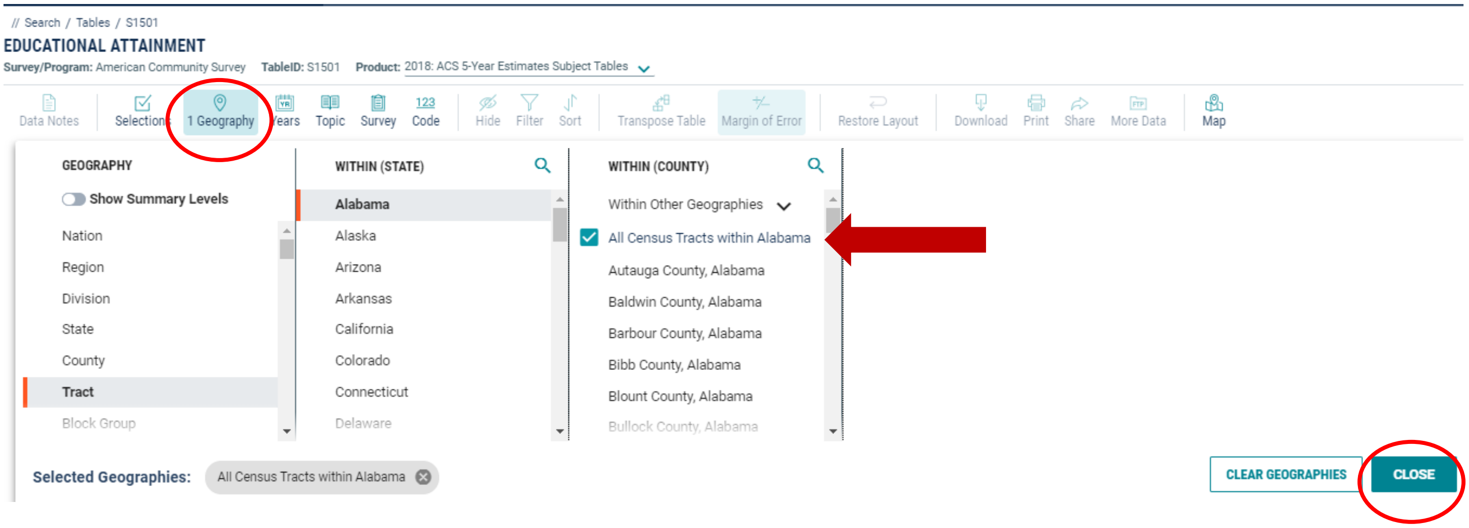

14. Make sure that the most recent year is selected for the 5-Year estimates. Download the S1501 table as a CSV file by clicking **Download**.

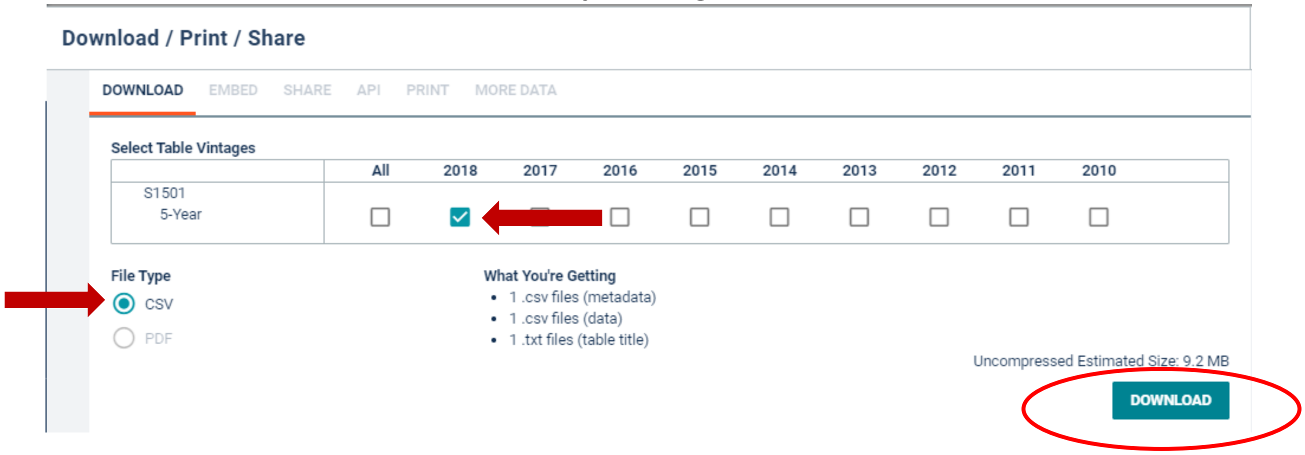

15. Click **Download Now.**

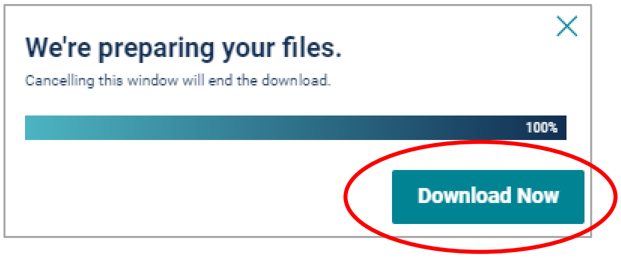

16. The downloaded file containing the data for the % less than a high school education data will have the title ACSST5Y2018.S1501_and contain the following three files:

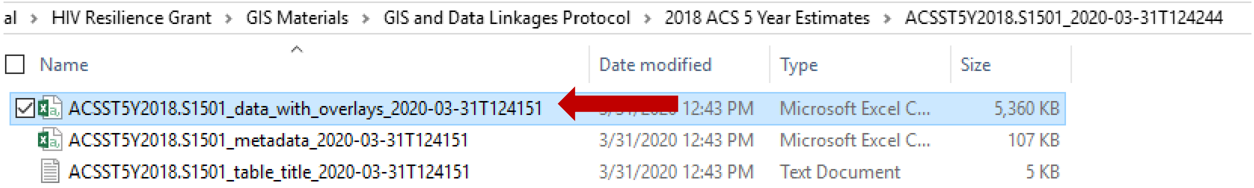

NOTE: The folder and file names will be slightly different depending on the date that the download was performed and the state for which the data were abstracted.

Save the **downloaded** file on your clinci desktop computer. Also, create an **Archive** subfolder for the additional socioeconomic data.

17. Unzip the file and open the **ACSST5Y2018.S1501_data_with_overlays** file in Excel.

    Rename the file so that it is specific to the socioeconomic indicator(s) and year of the ACS 5-year estimates (e.g., **Education_ACS_18)** and save the renamed file on your clinic desktop computer.

Part 5b. Reformatting the files that contain socioeconomic data

Reformatting the file that contains data for % Poverty and % Unemployed

10. Data for the % poverty and the % unemployed variables downloaded in the same Census Table DP03 (as described in **Step 2** of **Part 5a** of this protocol.)

Navigate to the file that contains the data for % below poverty line and % unemployed for all census tracts in your state. The file should be saved with a title similar to **PvrtyEmploy_ACS_18**.

- **Open** the file in Excel.

Understanding the data spreadsheet:

11. When the example **PvrtyEmploy_ACS_18** Excel file opens, Columns A - UD will contain data.
(This example file will contain 1,181 rows and 550 columns of data.)

To make the file easier to work with, you will delete the unnecessary data fields so that only the columns that contain data for the socioeconomic indicators of interest will be saved.

In this example, only the following four columns will be kept.

**Column:**	A	B	U	SS
**Field Header:**	GEO_ID	NAME	DP03_0005PE	DP03_0128PE
**Description of the data in this column:**	id	Geographic Area Name	Percent Estimate!!EMPLOYMENT STATUS!!Population 16 years and over!!In labor force!!Civilian labor force!!Unemployed	Percent Estimate!!PERCENTAGE OF FAMILIES AND PEOPLE WHOSE INCOME IN THE PAST 12 MONTHS IS BELOW THE POVERTY LEVEL!!All people

12. To make sure that you are deleting the correct data, in the Excel file:

- **Highlight** columns with the following field headers in yellow:
  GEO_ID (column A)
  NAME (column B)
  DP03_0005PE (column U)
  DP03_0128PE (column SS)

- **Delete** all other columns that are not highlighted.

13. Your worksheet should now contain 4 columns of data with the following field headers.
    a. GEO_ID
    b. NAME
    c. DP03_0005PE
    d. DP03_0128PE

14. Delete Row 2 (**Description of the data in this column)** from the dataset to remove the unneeded variable descriptors.

15. Reformat the values in the GEO_ID column so that the values indicate solely the specific census tract and do not include state-level information. To do this:

- Insert a new blank column to the right of the GEO_ID column.
- In cell B2, enter the formula: **=RIGHT(A2,LEN(A2)-9)** and apply the formula to all of column B.

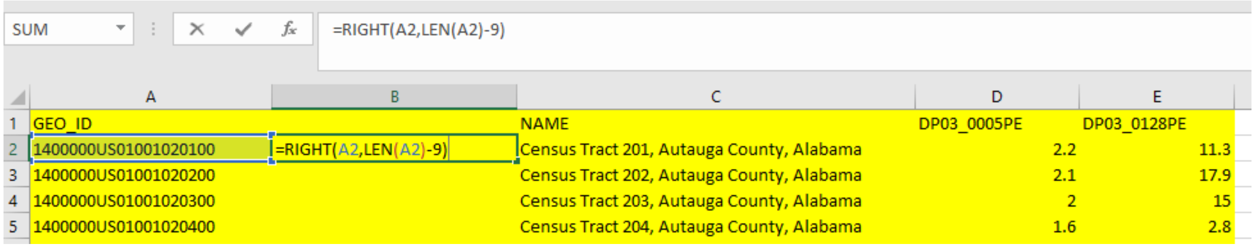

- Highlight cells B2:B1182. Copy and paste the data in these cells on top of the data in cells A2:A1182. Make sure to select **Paste Values**.

- Delete Column B.

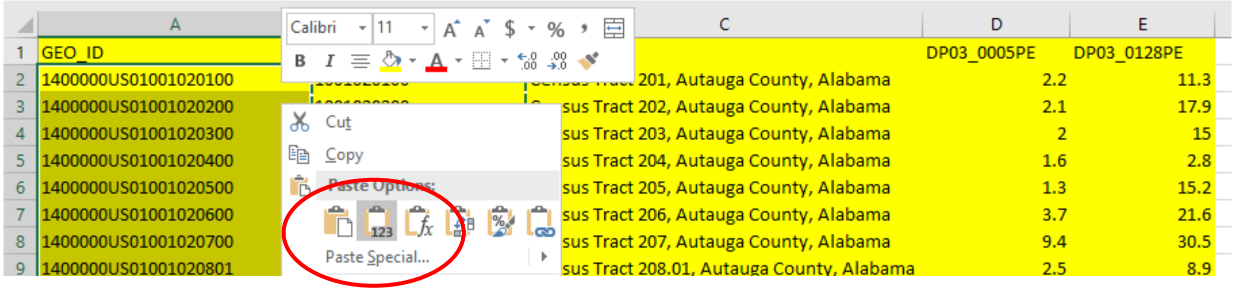

Your file should now resemble the below screenshot:

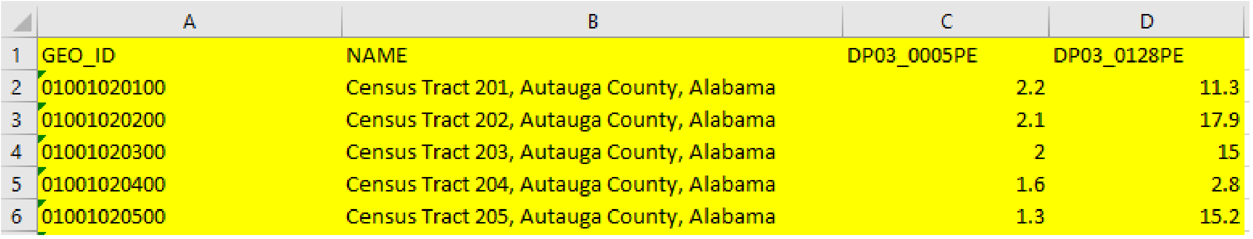

- Rename the **PvrtyEmploy_ACS_18** file to **PvrtyEmploy_ACS_18rf**.

- Save the reformatted file in the in a folder on your clinic desktop computer.
- **Close** the Excel file.

The data in this file will be merged with the other abstracted data and geocoded addresses in a final master Excel file as described in **Part 6** of this protocol.

The following files that were downloaded from data.census.gov should be archived by moving them to your **Archive** folder:

- ACSDP5Y2018.DP03_data_with_overlays_
- ACSDP5Y2018.DP03_metadata_
- ACSDP5Y2018.DP03_table_title_

Reformatting the file that contains data for % less than a high school education

16. Navigate to the Excel file that contains the data for % less than a high school education for all census tracts in your state. The file should be saved with a title similar to **Education_ACS_18**.

    - **Open** the file in Excel.

Understanding the data spreadsheet:

17. When the example Excel file opens, Columns A through ACQwill contain data. (The example file contains 1,183 rows and 770 columns of data.)

To make the file easier to work with, you will delete the unnecessary data fields so that only the columns that contain data for the socioeconomic indicators of interest will be saved.

In this example, only the following 3 columns will be kept.

**Column:**	A	B	XB
**Field Header:**	GEO_ID	NAME	S1501_C02_008E
**Description of the data in this column:**	id	Geographic Area Name	Estimate!!Percent!!Population 25 years and over!!9th to 12th grade, no diploma

18. To make sure that you are deleting the correct data, in the Excel file highlight the following columns in yellow:

    GEO_ID (column A)
    NAME (column B)

S1501_C02_008E (column XB)

- Delete all other columns that are not highlighted

Your worksheet should now only contain 3 columns of data with the following field headers:

- GEO_ID
- NAME
- S1501_C02_008E

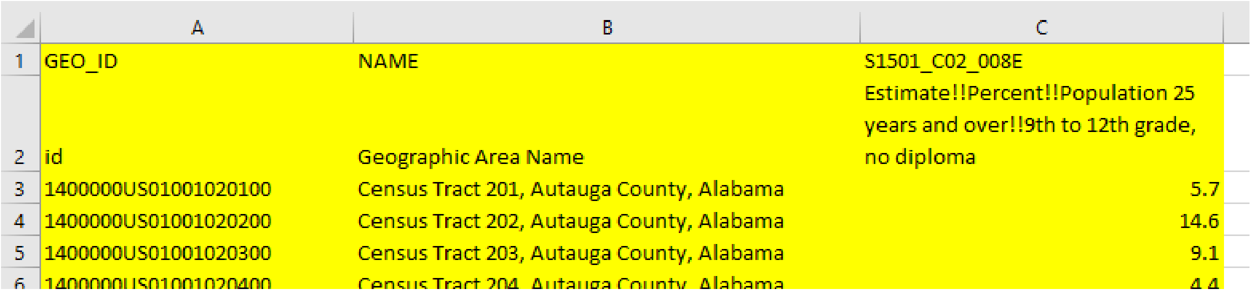

	A	B	C
1	GEO_ID	NAME	S1501_C02_008E
2	id	Geographic Area Name	Estimate!!Percent!!Population 25 years and over!!9th to 12th grade, no diploma
3	1400000US01001020100	Census Tract 201, Autauga County, Alabama	5.7
4	1400000US01001020200	Census Tract 202, Autauga County, Alabama	14.6
5	1400000US01001020300	Census Tract 203, Autauga County, Alabama	9.1
6	1400000US01001020400	Census Tract 204, Autauga County, Alabama	4.4

19. Delete Row 2 (**Description of the data in this column)** from the dataset to remove the unneeded variable descriptors.

20. Reformat the values in the GEO_ID column so that the values indicate solely the specific census tract and do not include state-level information. To do this:

- Insert a new blank column to the right of the GEO_ID column.
- In cell B2, enter the formula: **=RIGHT(A2,LEN(A2)-9)** and apply the entire formula to all of column B.

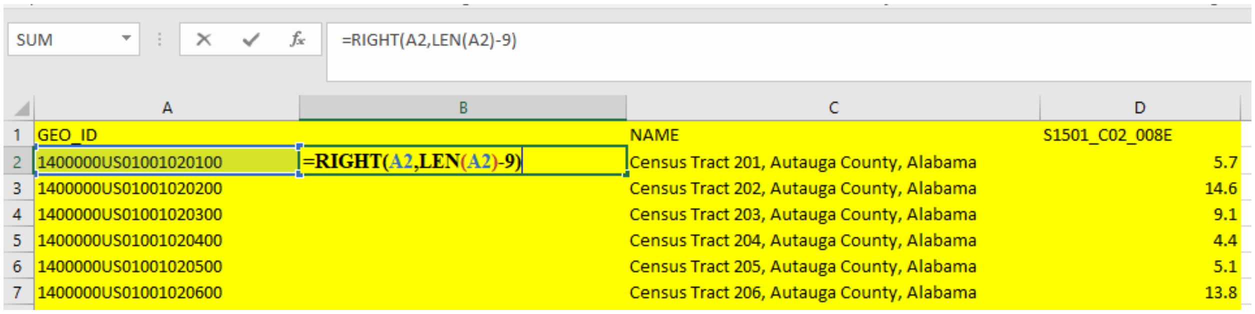

	A	B	C	D
1	GEO_ID		NAME	S1501_C02_008E
2	1400000US01001020100	=RIGHT(A2,LEN(A2)-9)	Census Tract 201, Autauga County, Alabama	5.7
3	1400000US01001020200		Census Tract 202, Autauga County, Alabama	14.6
4	1400000US01001020300		Census Tract 203, Autauga County, Alabama	9.1
5	1400000US01001020400		Census Tract 204, Autauga County, Alabama	4.4
6	1400000US01001020500		Census Tract 205, Autauga County, Alabama	5.1
7	1400000US01001020600		Census Tract 206, Autauga County, Alabama	13.8

- Highlight cells B2:B1182. Copy and paste the data in these cells on top of the data in cells A2:A1182. Make sure to select **Paste Values**.

- Delete Column B.

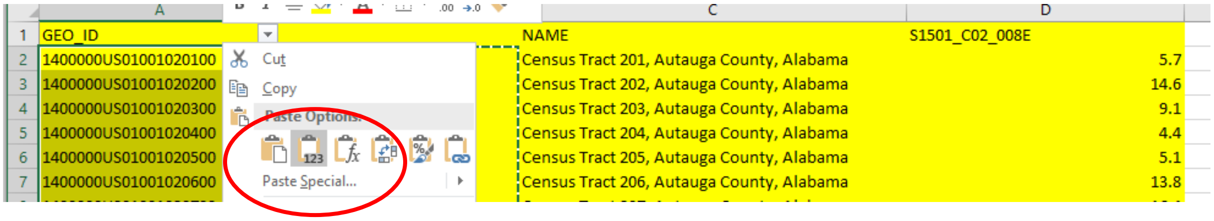

Your file should now resemble the below screenshot:

	A	B	C
1	GEO_ID	NAME	S1501_C02_008E
2	01001020100	Census Tract 201, Autauga County, Alabama	5.7
3	01001020200	Census Tract 202, Autauga County, Alabama	14.6
4	01001020300	Census Tract 203, Autauga County, Alabama	9.1
5	01001020400	Census Tract 204, Autauga County, Alabama	4.4
6	01001020500	Census Tract 205, Autauga County, Alabama	5.1
7	01001020600	Census Tract 206, Autauga County, Alabama	13.8
8	01001020700	Census Tract 207, Autauga County, Alabama	16.4

21. Rename the file **Education_ACS_18** as **Education_ACS_18rf** and save the reformatted file on your clinic desktop computer.

This completes the activities for reformatting the socioeconomic variables. In the next step, you will merge all of the data files into a master Excel file.

Part 6a. Adding abstracted and geocoded data into the Master Excel file

Objective: The objective is to prepare the de-identifiable Master Excel File that will be transferred to relevant collaborating sites. For this first section, you will insert the participants' geocoded data (i.e., census tracts, socioeconomic, and crime) into the file. Completing this activity will yield worksheet tabs that are ready to be joined with participants' data in Part 6b.

You will now have data in Excel files with the following names (or similar) that you will add into a single Master file.

1. SelectedTracts5.10.18rf
2. PvrtyEmploy_ACS_18rf
3. Education_ACS_18rf
4. Excel files that contain crime data per county

Step 1. Adding geocoded address data that are linked to census tracts to the Master file

1. Open the **SelectedTracts5.10.18rf** file that contains the census tract information for geocoded patient address locations.

2. Left Click on the top of Column B to highlight the column.

3. You will now sort the data in ascending order by <u>GEOID.</u>
   - In the top right hand corner, left click on Sort & Filter.
   - Click on Sort Smallest to Largest.
   - When the Sort Warning dialog box opens, select **Expand the Selection** and click **Sort.**
   - Save but do not close the document.

GEOID

	A	B	C	D	E	F
1	REDCap_ID	GEOID	NAME_1	NAMELSAD	GEOID10	NAMELSAD10
2	172-10	1001020300	203	Census Tract 203	1001	Autauga County
3	172-11	1003010500	105	Census Tract 105	1003	Baldwin County
4	172-12	1003010705	107.05	Census Tract 107.05	1003	Baldwin County
5	172-13	1003010800	108	Census Tract 108	1003	Baldwin County
6	172-14	1003011202	112.02	Census Tract 112.02	1003	Baldwin County
7	172-15	1003011501	115.01	Census Tract 115.01	1003	Baldwin County
8	172-16	1005950700	9507	Census Tract 9507	1005	Barbour County
9	172-17	1007010004	100.04	Census Tract 100.04	1007	Bibb County
10	172-18	1009050101	501.01	Census Tract 501.01	1009	Blount County
11	172-19	1011952200	9522	Census Tract 9522	1011	Bullock County
12						
13						

NOTE: If you are collecting participant address information more than one time points during your study, you will have geocoded address information for each enrollment and follow up study visit that a patient attends, such that your file may look similar to the following:

	A	B	C	D	E	F
1	REDCAPID	GEOID	NAME_1	NAMELSAD	GEOID10	NAMELSAD10
2	172-10	1001020300	203	Census Tract 203	1001	Autauga County
3	172-11	1003010500	105	Census Tract 105	1003	Baldwin County
4	172-11	1003010500	105	Census Tract 105	1003	Baldwin County
5	172-12	1003010705	107.05	Census Tract 107.05	1003	Baldwin County
6	172-12	1003010705	107.05	Census Tract 107.05	1003	Baldwin County
7	172-12	1007010004	100.04	Census Tract 100.04	1007	Bibb County
8	172-13	1003010800	108	Census Tract 108	1003	Baldwin County
9	172-13	1003010800	108	Census Tract 108	1003	Baldwin County
10	172-13	1009050101	501.01	Census Tract 501.01	1009	Blount County
11	172-13	1009050101	501.01	Census Tract 501.01	1009	Blount County
12	172-14	1003011202	112.02	Census Tract 112.02	1003	Baldwin County

4. You will now copy and paste Columns A-F of the **SelectedTracts5.10.18rf** file into the Master Excel file.
   - Place your cursor over the A at the top of Column A.
   - Left click to highlight the column.
   - Holding the cursor down, scroll to the right until you come to column F. (This will highlight each of the 6 columns so that you can copy and paste them.)
   - Right click on F at the top of column F. A dropdown menu will open.
   - Scroll down to Copy and left click.

- Right click on cell AI in the **Joined Data REDCap_ID** worksheet in the Master Excel sheet to open the dropdown menu.
- Scroll down to Paste options.
- Select the first option to Keeps Source Formatting.

The six columns from **SelectCT5.10.18rf** have been pasted into the Master Excel file.

- **Close** the **SelectCT5.10.18rf** Excel file.
- **Save** and keep open Master Excel file.

Step 2. Adding data for % less than a high school education to the Master file

You will now add the data for % less than a high school education from the **Education_ACS_18rf** Excel file to the ACS worksheet in the **Master** Excel file.

5. Open **the Education_ACS_18rf** file that contains the census tract-level data for the socioeconomic indicator % less than a high school education.

6. **Save** but do not close the document.

7. You will now sort the data in ascending order by GEOD_ID.
    - Left click on the top of Column A to highlight the column.
    - In the top right hand corner **left click** on **Sort & Filter.** Click on **Sort Smallest to Largest**.
    - When the Sort Warning dialog box opens, select **Expand the Selection** and click **Sort.**
    - **Save** but do not close the document.

8. You will now copy and paste Columns A-C from the **Education_ACS_18rf** into the Master Excel file.
    - Place your cursor over the A at the top of Column A.
    - Left click to highlight the column.
    - Holding the cursor down, scroll to the right until you come to Column C. (This will highlight each of the 3 columns so that you can copy and paste them.)
    - Right click on C at the top of Column C.
    - A Dropdown menu will open. Scroll down to Copy and left click.
    - Left Click on the ACS Tab in the worksheet Master Excel file that will hold all of the ACS data.
    - Right click on cell A1 in the ACS worksheet in the Master Excel file.
    - Right click to open the dropdown menu.
    - Scroll down to Paste Options.
    - Select the first option that Keeps Source Formatting.

The 3 columns from **Education_ACS_18rf** have been pasted into the Master file.

- Close the Education_ACS_ file.

- Save but keep open the Master Excel file.

Step 3. Adding data for % poverty and % unemployed to the Master file

You will now add the data for % below the poverty line and % unemployed from the **PvrtyEmploy_ACS_18rf** Excel file to the ACS worksheet in the Master Excel file.

9. Open **the PvrtyEmploy_ACS_18rf** file that contains the census tract-level data for the socioeconomic indicators % Poverty and % Unemployed.

10. Save but do not close the document.

11. You will now sort the data in ascending order by GEOD_ID.
    - Left click on the top of Column A to highlight the column.
    - In the top right hand corner **left click** on **Sort & Filter.** Click on **Sort Smallest to Largest**.
    - When the Sort Warning dialog box opens select Expand the Selection and click **Sort.**
    - **Save** but do not close the document.

12. You will now copy and paste Columns A-D into the Master Excel file.
    - Place your cursor over the A at the top of Column A.
    - Left click to highlight the column.
    - Holding the cursor down, scroll to the right until you come to Column D. (This will highlight each of the 4 columns so that you can copy and paste them.)
    - Right click on A at the top of Column A.
    - A Dropdown menu will open. Scroll down to Copy and left click
    - Left click on the ACS worksheet in the Master Excel file that will contain the data for % poverty and % unemployment.
    - Right click on cell D1 in the ACS worksheet in the Master Excel sheet.
    - Right click to open the dropdown menu.
    - Scroll down to Paste Options.
    - Select the first option that **Keeps Source Formatting.**

The 4 columns of data from **Poverty_ACS_18rf** have been pasted into the **Master** Excel file. The ACS worksheet in the Master Excel file should now have data in Columns A through G.

13. Delete the data in Column D and Column E because these data are the same as the data for GEO_ID and GEO.display-label in Column A and Column B.

    NOTE: In the ACS tab in the Master Excel file, make sure that all data for the GEOIDs are stored as <u>numbers</u> and are not stored as text.

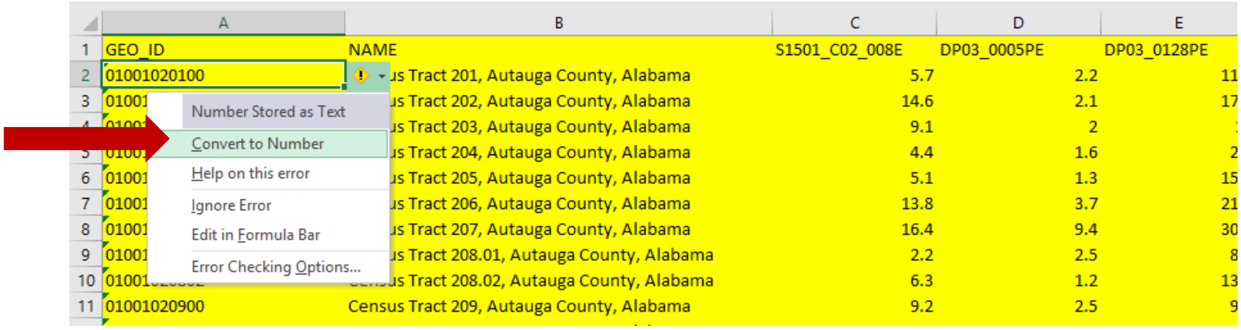

- Close the **Poverty_ACS** file.
- Save but keep open R01_GISData_Master Excel file.

Step 4. Adding crime data to the Master file

You will now add the crime data from the example **R01.Crime.AL.Date** Excel file to the Crime worksheet in the Master Excel file.

14. Open the file that contains the census tract-level data for the relevant crime indices.

15. Left Click on the top of Column A to highlight the column.

16. In Column A, sort the data in ascending order by Census Tract.
    - Left click on the top of Column A to highlight the column.
    - In the top right hand corner left click on Sort & Filter. Click on Sort Smallest to Largest.
    - When the Sort Warning dialog box opens, select Expand the Selection and click Sort.
    - Save but do not close the document.

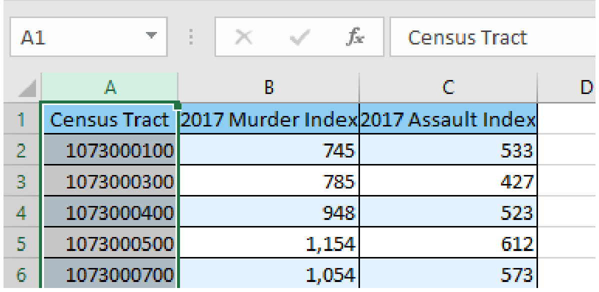

17. You will now copy and paste Columns A-C from the **R01.Crime.AL.Date** worksheet into the Master Excel file.

    - Place your cursor over the A at the top of Column A.
    - Left click to highlight the column.
    - Holding the cursor down, scroll to the right until you come to Column C (This will highlight each of the 3 columns so that you can copy and paste them.)
    - Right click on C at the top of Column C.
    - A Dropdown menu will open. Scroll down to Copy and left click.

- Left click on the Crime tab to open the worksheet that will contain the crime data.
- Right click on cell A1 in the Crime worksheet in the Master Excel file.
- Right click to open the dropdown menu. Scroll down to Paste Options
- Select the first option that Keeps Source Formatting

The 3 columns from **R01.Crime.AL.Date** Excel file have been pasted into the Master file.

- **Close** the **R01.Crime.AL.Date** file**.**

- **Save** but keep open the Master Excel file.

Part 6b. Linking data by Census tractin the Master file

Objective: The objective is to join data to the appropriate census tracts in which participants reside. You will use the built-in Excel Macro to join these data. Completing this activity will yield a file that will be ready to have coded census tracts; creating coded census tracts will be completed in Part 7.

The Master Excel file has a built in Macro that will join each data variable to the appropriate census tract. When performing any data manipualtion, you will use this Master file.

18. To join the data that you have added to the Master Excel file, left click on the **JoinedDataRedCapID** tab.

    - Click on View in the ribbon at the top of the R01Master Excel file.

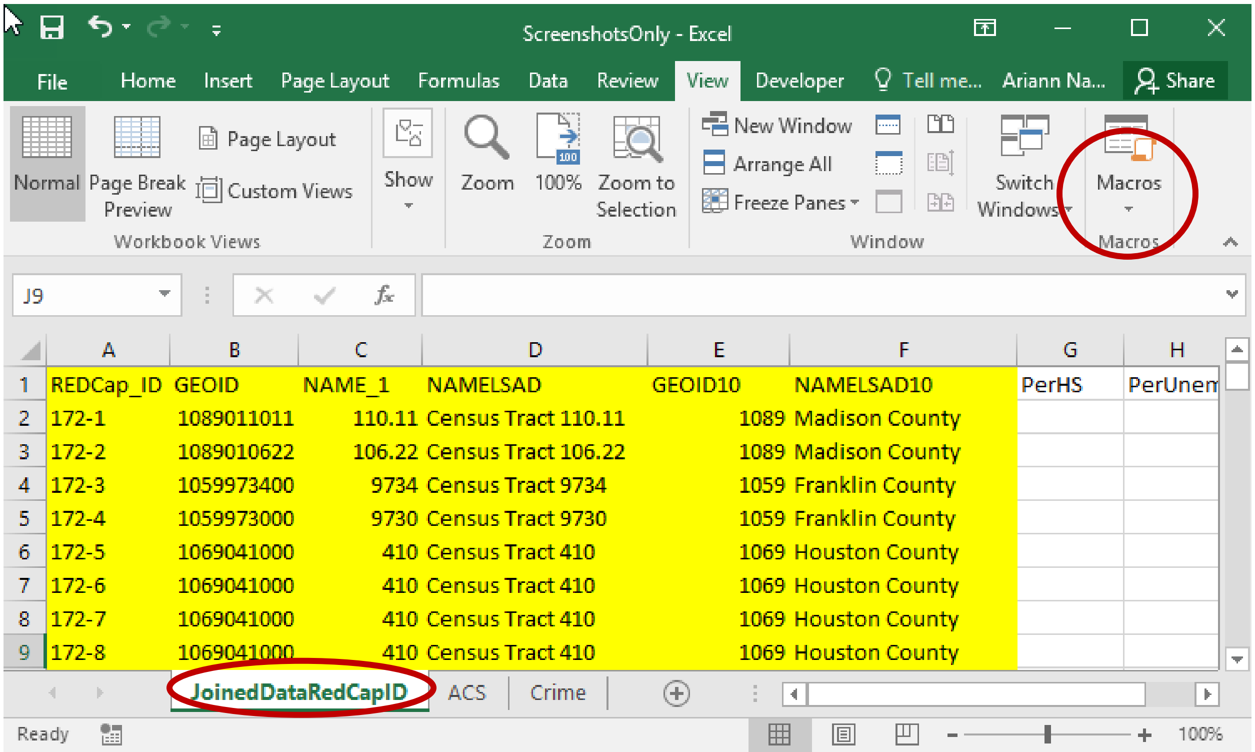

    - Select cell G2 by placing the cursor in the cell and left clicking.
    - Left click on the Macro icon in the top right hand corner of the file.
    - Left click to select **View Macros.**

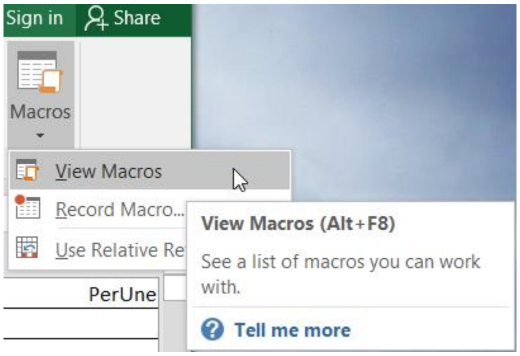

19. The Macro Dialog Box will open.
    - Left click on **ACS Matcher** to select it.
    - Click **Run.**

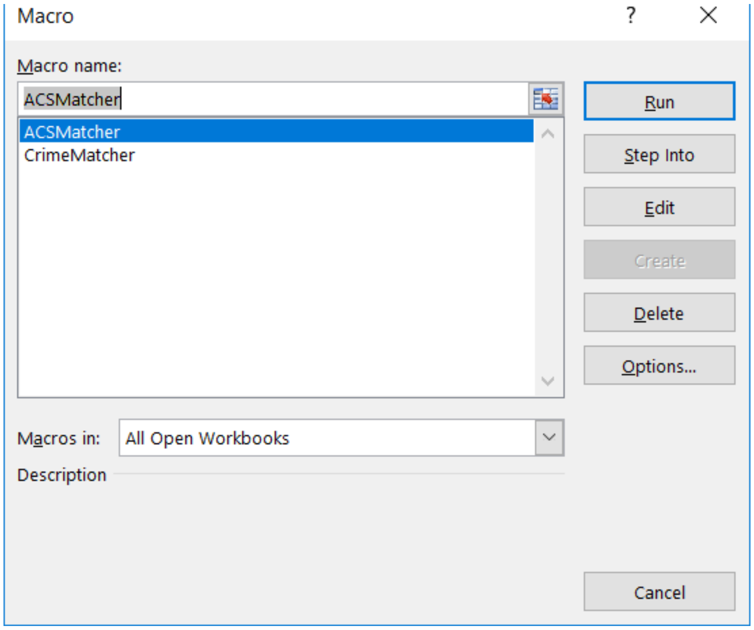

20. Each cell in Column G - I will autopopulate with the correct data for that variable that matches with the Census tractId in each row.

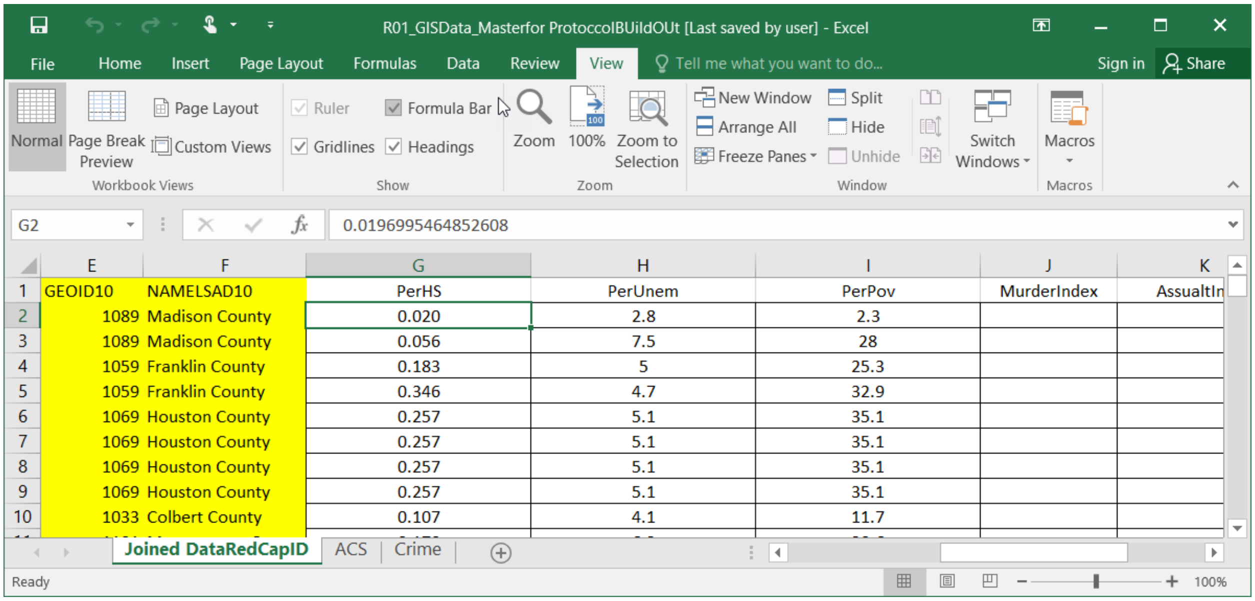

21. You will now use the CrimeMatcher Macro to match the crime data to the appropriate Census tractin each row.
- Select cell J2 by placing the cursor in the cell and left clicking.
- Left click on the Macro icon in the top right hand corner of the document.
- Left click to select **View Macros.**
- The Macro Dialog Box will open.
- Left click on **CrimeMatcher** to select it.
- Click **Run.**

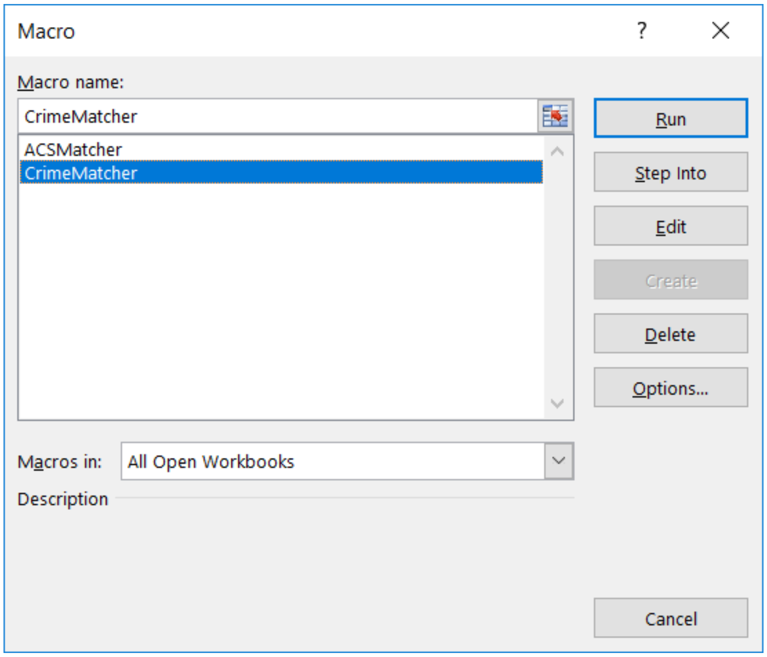

Cells in Column J and K will autopoulate with the data variable that matches the Census tractin each row.

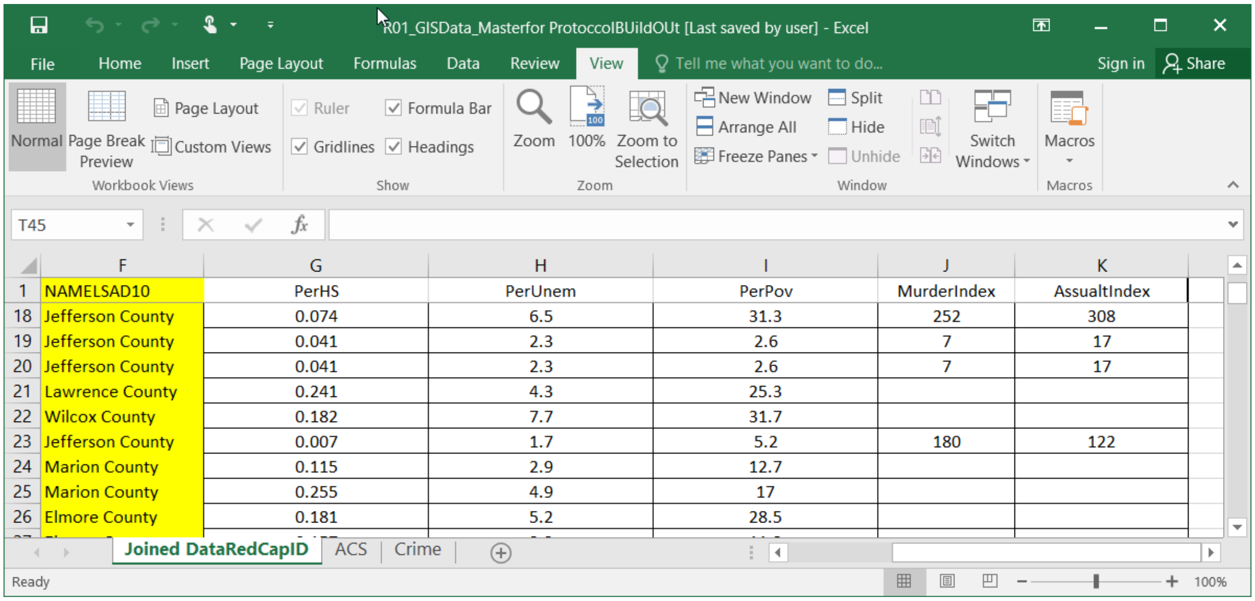

	F	G	H	I	J	K
1	NAMELSAD10	PerHS	PerUnem	PerPov	MurderIndex	AssualtIndex
18	Jefferson County	0.074	6.5	31.3	252	308
19	Jefferson County	0.041	2.3	2.6	7	17
20	Jefferson County	0.041	2.3	2.6	7	17
21	Lawrence County	0.241	4.3	25.3		
22	Wilcox County	0.182	7.7	31.7		
23	Jefferson County	0.007	1.7	5.2	180	122
24	Marion County	0.115	2.9	12.7		
25	Marion County	0.255	4.9	17		
26	Elmore County	0.181	5.2	28.5		

22. Save the R01Master file but do not close the file.

Part 7. Deleting Census Tract Identifiers and Creating Coded Identifiers

1. Open a new Worksheet in the Master Excel file and name the sheet so that it is specific to your study and indicates that the sheet will included the coded census tracts. Coded census tracts will be represented by a "Dummy Variable" (i.e., "DV").

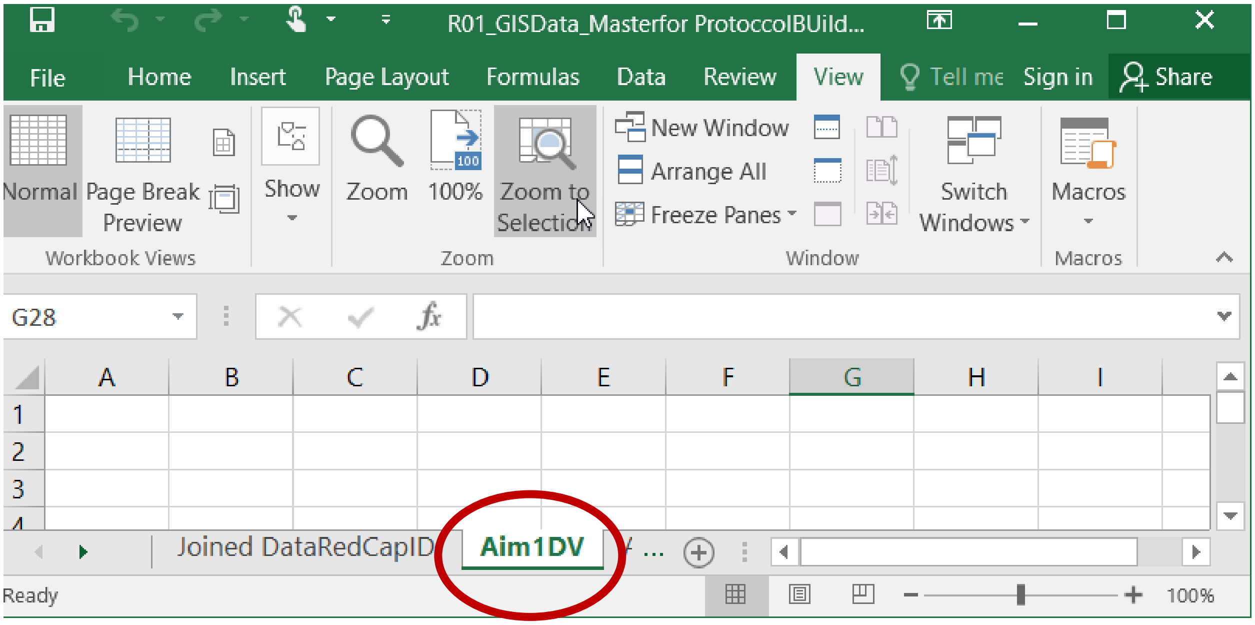

2. You will now copy the contents of the **Joined DataRedCapID** worksheet to the newly created worksheet.

   - Open the **Joined DataRedCapID** tab.
   - Left click the top corner of the data cells to select the contents of the worksheet.
   - Right click and select Copy from the dropdown menu.

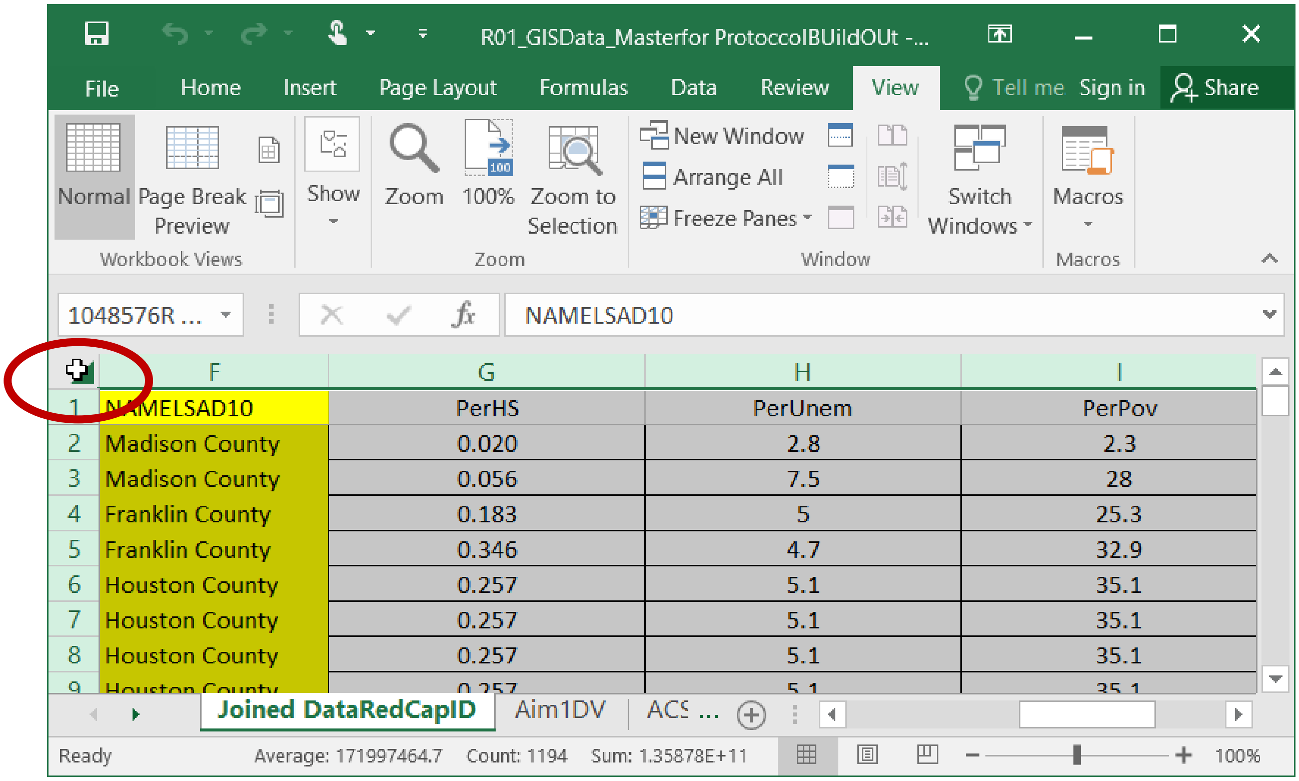

- Open the newly created worksheet and right click on cell A1.
  Note. This worksheet tab name will be study-specific. In this example protocol, the newly created worksheet that will contain coded census tracts is titled Aim1DV.

- From the drop down menu select **Paste.**

Step 1. Deleteing census tractand county identifiers and assigning random numbers to create coded census tracts

3. You will now assign a coded identifier to the census tracts by creating a column of dummy variables, removing all census tract identifiers from the worksheet, and replacing the original identfiers with the newly created dummy variables.

- In the Aim1DV worksheet, right click the top of column C to highlight the column and open the drop down menu.
- Left click **Insert** from the drop down menu.
- Insert three columns so that there are now three blank columns in columns C, D and E.
- Type “DV” (dummy variable) as the field header name for column C.
- Type “DVRANK” (ranked dummy variable) as the field header name for column D.
- Type “DVF” (dummy variable final) as the field header name for column E.
- Save the file.

4. Using Excel's Rand and Rank functions you will now create a column of unique random numbers that will replace the census tractIDs

   - Select cell **C2.**
   - Type **<u>=RAND()</u>** into the Function box.

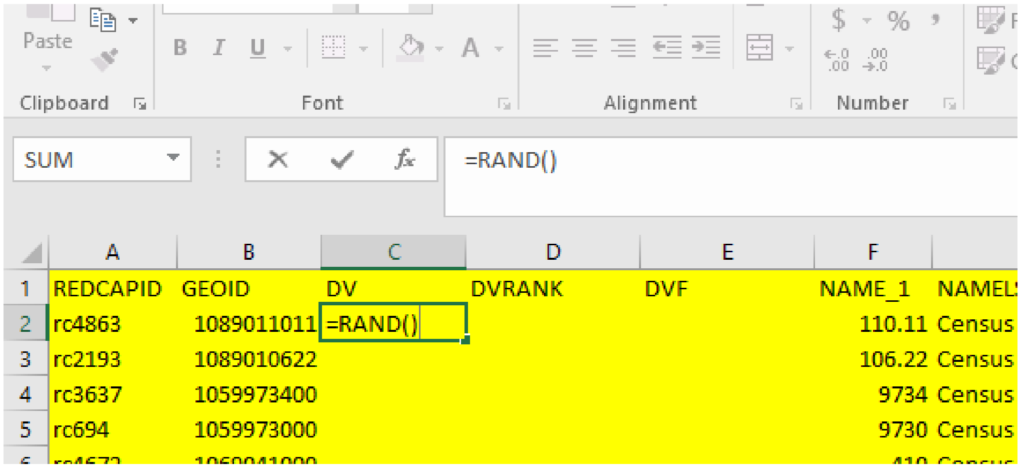

   - Press **Enter**.

     Note: Your random values will not match the screenshots in this section.

   - Left-click on cell C2 and position the cursor on the bottom right hand corner of the cell. Be sure that the cursor has changed into a + shape shown below.

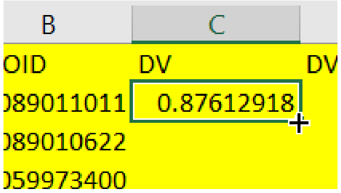

   - Double-left-click. This will automatically fill in the entire column.
   - In Cell D2 type **<u>=RANK(C2,C:C)</u>** in the function box and press enter.

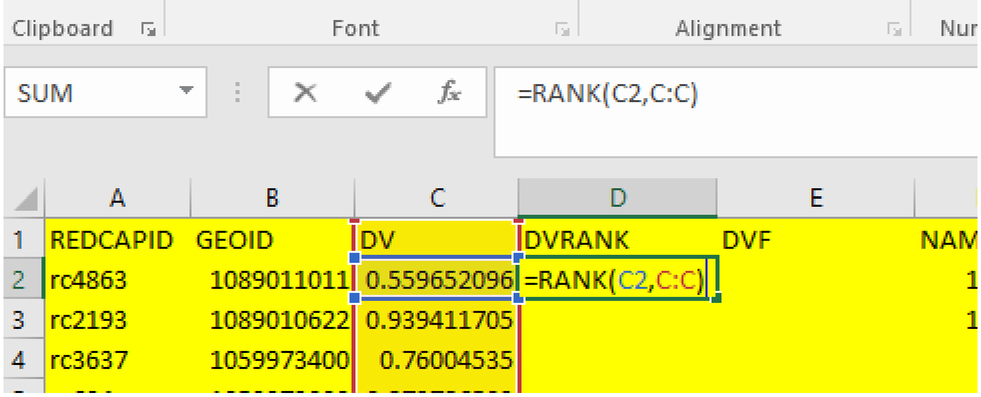

- Left-click on cell D2 and position your cursor on the bottom right hand corner of the cell. Be sure that the cursor has changed into a + shape. Double-left-click. This will automatically fill in the entire column.

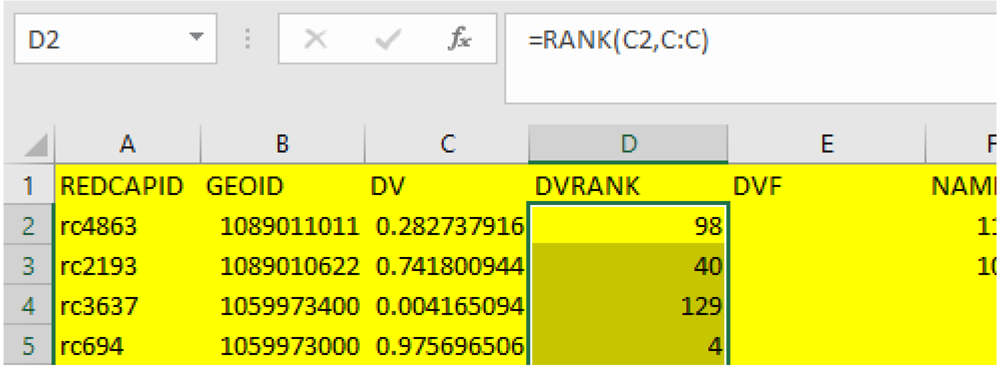

D2 =RANK(C2,C:C)

	A	B	C	D	E	F
1	REDCAPID	GEOID	DV	DVRANK	DVF	NAM[illegible]
2	rc4863	1089011011	0.282737916	98		1[illegible]
3	rc2193	1089010622	0.741800944	40		1[illegible]
4	rc3637	1059973400	0.004165094	129		
5	rc694	1059973000	0.975696506	4		

- We will need to ensure that the final coded census tracts are unique to each clinic site and that there are no duplicates between sites. (In this example, we are working with data from two unique clinic sites.) To do this:

  - In Cell E2, <u>Site 1</u> should type **=IF(B2="","",(CONCATENATE("x",D2)))**[6]

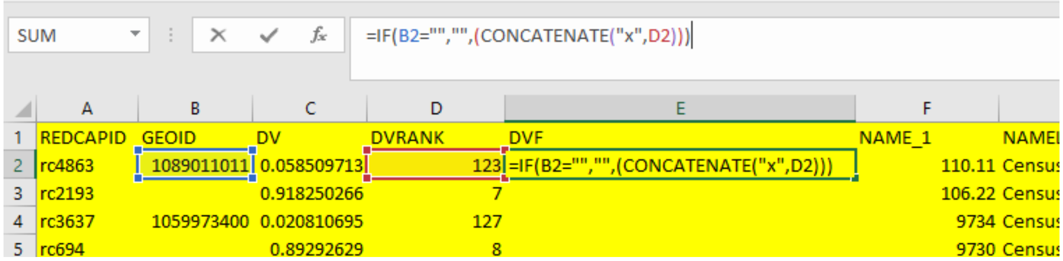

SUM =IF(B2="","",(CONCATENATE("x",D2)))

	A	B	C	D	E	F	
1	REDCAPID	GEOID	DV	DVRANK	DVF	NAME_1	NAMEI
2	rc4863	1089011011	0.058509713	123	=IF(B2="","",(CONCATENATE("x",D2)))		110.11 Censu[illegible]
3	rc2193		0.918250266	7			106.22 Censu[illegible]
4	rc3637	1059973400	0.020810695	127			9734 Censu[illegible]
5	rc694		0.89292629	8			9730 Censu[illegible]

  - In Cell E2, <u>Site 2</u> should type **=IF(B2="","",(CONCATENATE("b",D2)))**[8]

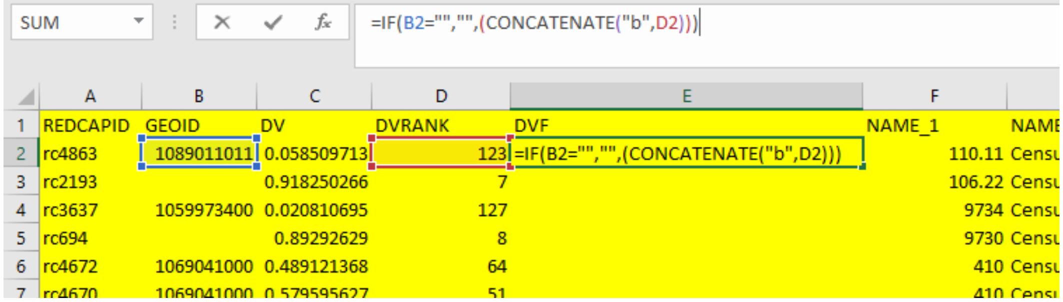

SUM =IF(B2="","",(CONCATENATE("b",D2)))

	A	B	C	D	E	F	
1	REDCAPID	GEOID	DV	DVRANK	DVF	NAME_1	NAME
2	rc4863	1089011011	0.058509713	123	=IF(B2="","",(CONCATENATE("b",D2)))		110.11 Censu
3	rc2193		0.918250266	7			106.22 Censu
4	rc3637	1059973400	0.020810695	127			9734 Censu
5	rc694		0.89292629	8			9730 Censu
6	rc4672	1069041000	0.489121368	64			410 Censu
7	rc4670	1069041000	0.579595627	51			410 Censu

- Left-click on cell E2 and position your cursor on the bottom right hand corner of the cell. Be sure that the cursor has changed into a + shape. Double-left-click. This will automatically fill in the entire column.
- Save the Master Excel file but do not close the file.

---

[6] Study IDs without addresses (e.g., when a patients' address is a P.O. Box or is outside of the study state) will not be able to be geocoded or linked to census tracts. Applying this formula will ensure that Study IDs without addresses (i.e., for which there are no data for the GEOID variable) do not have any data for the DVF variable.

5. You will now copy and paste the contents of worksheet AIM1DV to a new worksheet.

    - Add another sheet to the excel book.
    - Name this new worksheet so that it is specific to your study and indicates it is the final data that will be sent to the external coordinating institution for analysis (e.g., Aim1Final).
    - Click on the top-left corner to select the entire worksheet. Right click and copy.
    - On the new sheet, right-click cell A1. On the drop-down menu, select **Paste Values (**see the screenshot below).

Note: It is extremely important that you use **Paste Values** when copying the data from the **Aim1DV** worksheet to the **Aim1Final** worksheet. This will paste the data values rather than the formulas into the Aim1Final worksheet.

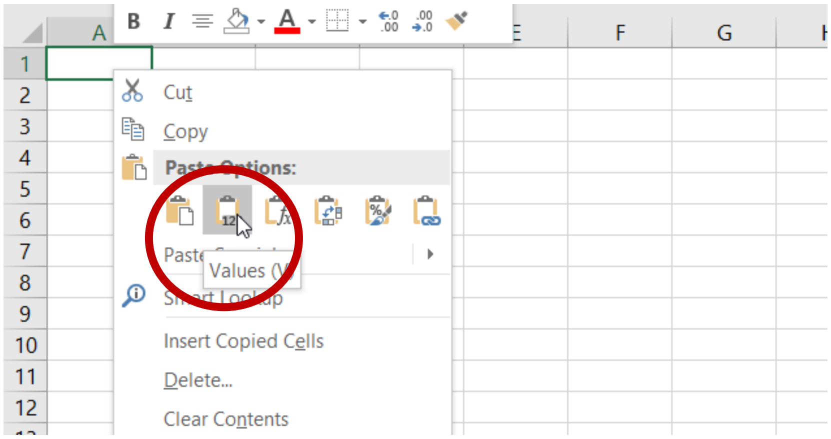

6. In the **Aim1Final** worksheet:
   - Delete the columns with the "DV" (dummy variable) and "DVRANK" (ranked dummy variable) data.
   - Select Columns B and C.
   - Select "Conditional Formatting" in the ribbon at the top. Click on "Hightlight Cells Rules" and then click "Duplicate Values."

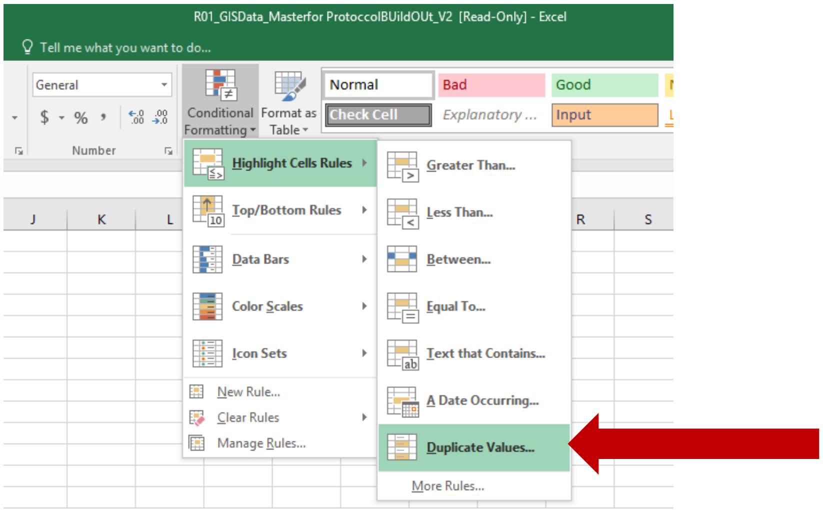

   - The Duplicate Values dialogue box will open.
   - Select "OK" on the dialogue box.

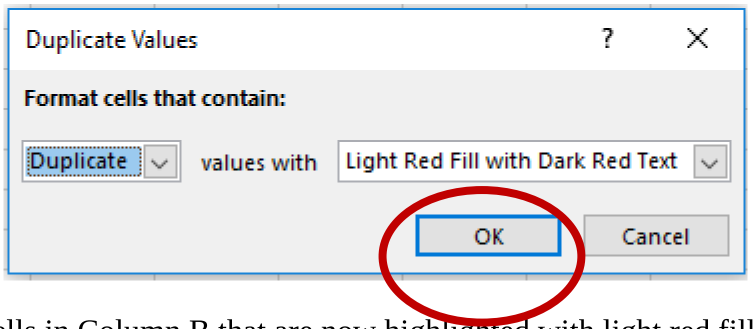

7. There will be cells in Column B that are now highlighted with light red fill and dark red text as seen in the screenshot below.

	A	B	C	D	E	F	G
1	REDCAP_ID	GEOID	DVF	NAME_1	NAMELSAD	GEOID10	NAMELSAD10
2	172-1	1089011011	128	110.11	Census Tract 110.11	1089	Madison County
3	172-2	1089010622	78	106.22	Census Tract 106.22	1089	Madison County
4	172-3	1059973400	113	9734	Census Tract 9734	1059	Franklin County
5	172-4	1059973000	87	9730	Census Tract 9730	1059	Franklin County
6	172-5	1069041000	42	58	Census Tract 410	1069	Houston County
7	172-6	1069041000	32	103	Census Tract 410	1069	Houston County
8	172-7	1069041000	29	30	Census Tract 410	1069	Houston County
9	172-8	1069041000	17	49	Census Tract 410	1069	Houston County
10	172-9	1033020704	89	44	Census Tract 207.04	1033	Colbert County
11	172-10	1101003200	58	64	Census Tract 32	1101	Montgomery County
12	172-11	1101003200	103	32	Census Tract 32	1101	Montgomery County
13	172-12	1119011500	30	115	Census Tract 115	1119	Sumter County
14	172-13	1103000100	49	1	Census Tract 1	1103	Morgan County
15	172-14	1113030402	44	304.02	Census Tract 304.02	1113	Russell County
16	172-15	1107050300	5	503	Census Tract 503	1107	Pickens County
17	172-16	1127020300	125	203	Census Tract 203	1127	Walker County
18	172-17	1073004902	79	49.02	Census Tract 49.02	1073	Jefferson County
19	172-18	1073012905	4	129.05	Census Tract 129.05	1073	Jefferson County
20	172-19	1073012905	111	129.05	Census Tract 129.05	1073	Jefferson County
21	172-20	1079979400	80	9794	Census Tract 9794	1079	Lawrence County
22	172-21	1131034800		348	Census Tract 348	1131	Wilcox County

Note: Column B contains the original geographic identifiers for each census tract. The cells highlighted in red in this column are duplicate geographic identifiers. These duplicates are census tracts that occur more than once. This happens because multiple patients will reside within the same census tract.

Duplicate census tracts MUST have the same coded census tract identifier.

8. After completeing the previous steps 1 - 6 you will have created a unique dummy variable to use as the coded identifier for each census tract. Duplicate GeoIDs need to have the same coded dummy variable. Duplicate GeoIDs will be grouped together. To assign the same coded dummy variable to duplicate GeoIDs, select the corresponding cell for that GeoID from Column C. From the bottom-right corner of the cell, click and drag down the column to the end of the GeoID grouping. Do this for each grouping of duplicate GeoIDs.

	A	B	C	D	E	F	G
1	REDCAP_ID	GEOID	DVF	NAME_1	NAMELSAD	GEOID10	NAMELSAD10
2	172-1	1089011011	128	110.11	Census Tract 110.11	1089	Madison County
3	172-2	1089010622	78	106.22	Census Tract 106.22	1089	Madison County
4	172-3	1059973400	113	9734	Census Tract 9734	1059	Franklin County
5	172-4	1059973000	87	9730	Census Tract 9730	1059	Franklin County
6	172-5	1069041000	42	58	Census Tract 410	1069	Houston County
7	172-6	1069041000	42	103	Census Tract 410	1069	Houston County
8	172-7	1069041000	42	30	Census Tract 410	1069	Houston County
9	172-8	1069041000	42	49	Census Tract 410	1069	Houston County
10	172-9	1033020704	89	44	Census Tract 207.04	1033	Colbert County
11	172-10	1101003200	58	64	Census Tract 32	1101	Montgomery County
12	172-11	1101003200	103	32	Census Tract 32	1101	Montgomery County

9. In the following steps you will check that duplicate GeoIDs are assigned the same dummy variable.
   - Scroll down through column B and identify the duplicate values (i.e., the cells that are highlighted in red.)
   - Copy the dummy variable of the first duplicate and paste it into the cells in column C next to each duplicate GeoID

In the example below the duplicate GeoID in Column B is 1069041000 (there are four duplicates GeoIDs in that group in rows 6 – 9.)

The first dummy variable for this GeoID is 58.

5	172-4	1059973000	89	9730	Census Tract 9730
6	172-5	1069041000	58	58	Census Tract 410
7	172-6	1069041000	103	103	Census Tract 410
8	172-7	1069041000	30	30	Census Tract 410
9	172-8	1069041000	49	49	Census Tract 410
10	172-9	1033020704	44	44	Census Tract 207.04
11	172-10	1101003200	64	64	Census Tract 32

- Copy “58” and paste it into the three cells below so that each cell containing GeoID 1069041000 contains the dummy variable 58.

5	172-4	1059973000	89	9730	Census Tract 9730
6	172-5	1069041000	58	58	Census Tract 410
7	172-6	1069041000	58	103	Census Tract 410
8	172-7	1069041000	58	30	Census Tract 410
9	172-8	1069041000	58	49	Census Tract 410
10	172-9	1033020704	44	44	Census Tract 207.04
11	172-10	1101003200	64	64	Census Tract 32

- Go through all duplicate values in Column B and manually copy and paste the dummy variable into to each adjacent cell.

- Save and close the Master Excel file. The data are ready for transfer to the coordinating instution.
