# Supplementary material for: Characterizing the neighborhood risk environment in multisite clinic-based cohort studies: A practical geocoding and data linkages protocol for protected health information
Source: PLoS One. 2022 Dec 29;17(12):e0278672. doi: 10.1371/journal.pone.0278672 (PMC9799318; doi:10.1371/journal.pone.0278672)
Supplement: S3 File — (PDF) [file pone.0278672.s003.pdf]

Supporting Information File 3 (S3)

Supplementary material for the manuscript:

Characterizing the Neighborhood Risk Environment in Multisite Clinic-Based Cohort Studies: A Practical Geocoding and Data Linkages Protocol for Protected Health Information

Ariann Nassel[1¶], Marta G. Wilson-Barthes[2¶*], Chanelle J. Howe[2], Sonia Napravnik[3], Michael J. Mugavero[4], Deana Agil[3], Akilah J. Dulin[5]

[¶] Ariann Nassel and Marta G. Wilson-Barthes are co-first authors; both first authors contributed equally to this manuscript.

[*] Corresponding author
Email: marta_wilson-barthes@brown.edu (MWB)

[1] Lister Hill Center for Health Policy, School of Public Health, University of Alabama at Birmingham, Birmingham, Alabama, United States of America.
[2] Center for Epidemiologic Research, Department of Epidemiology, Brown University School of Public Health, Providence, Rhode Island, United States of America.
[3] Division of Infectious Diseases, Department of Medicine, School of Medicine, Department of Epidemiology, Gillings School of Global Public Health, University of North Carolina at Chapel Hill, Chapel Hill, North Carolina, United States of America.
[4] Division of Infectious Diseases, Department of Medicine, Center for AIDS Research, University of Alabama at Birmingham, Birmingham, Alabama, United States of America.
[5] Center for Health Promotion and Health Equity, Department of Behavioral and Social Sciences, Brown University School of Public Health, Providence, Rhode Island, United States of America.

Supporting Information File 3 (S3)

Description: This supplementary material outlines the steps that can be used to create an Index of Neighborhood Disadvantage using socioeconomic data ascertained from US Census Bureau American Community Survey (ACS) Five-Year Estimates. The Neighborhood Disadvantage Index (NDI) represents a summation of the census-tract level z-scores for each socioeconomic variable that have been normed to the national level.

Steps to construct the Neighborhood Disadvantage Index (NDI)

Step 1: Create a Z-score for each sample variable that is normed to the United States (US). To do this, subtract each US variable's mean from its sample variable counterpart's mean and then divide by the US variable counterpart's standard deviation:

$$Z = (x - \mu)/\sigma$$

where x is the mean of the sample variable ascertained for the relevant census tract from American Community Survey Five-Year Estimates; μ is the mean of the sample variable ascertained for all US Census Tracts from American Community Survey Five-Year Estimates; and σ is the standard deviation (SD) of the sample variable ascertained for all US Census Tracts from American Community Survey Five-Year Estimates.

Step 2: Sum the Z-score variables for each socioeconomic variable to create the Neighborhood Disadvantage Index.

Step 3: Assign the appropriate risk level to the index data using categories appropriate for the sample distribution (e.g., binary split, tertiles).

Example NDI calculation

**Example faux data**		
Variable	Mean (SD) for census tract containing at least one geocoded patient address	Mean (SD) for all US census tracts ascertained from ACS 5-Year Estimates
Percent less than a high school education	17.7 (5.8)	21.3 (7.2)
Percent unemployed	3.1 (1.9)	9.5 (3.8)
Percent below the poverty line	15.8 (4.2)	10.5 (2.1)

Step 1. Create a Z-score for each sample variable that is normed to the United States (US).

ZPerHS = (17.7 – 21.3) / 7.2 = -0.50

ZPerUnem = (3.1 – 9.5) / 3.8 = -1.68

ZPerPov = (15.8 – 10.5) / 2.1 = 2.52

Step 2: Sum the Z-score variables for each socioeconomic indicator to create the Neighborhood Disadvantage Index.

NDI = (-0.50) + (-1.68) + (2.52) = 1.34
